# Genetic Diversity of the Collection of Far Eastern *Actinidia* spp. Revealed by RAD Sequencing Technology

**DOI:** 10.3390/plants14010007

**Published:** 2024-12-24

**Authors:** Natalia Slobodova, Maria Gladysheva-Azgari, Fedor Sharko, Kristina Petrova, Eugenia Boulygina, Svetlana Tsygankova, Irina Mitrofanova

**Affiliations:** 1 N.V. Tsitsin Main Botanical Garden, Russian Academy of Sciences, 127276 Moscow, Russia; 2 Faculty of Biology and Biotechnology, HSE University, 101000 Moscow, Russia; 3 National Research Center “Kurchatov Institute”, 1st Akademika Kurchatova Square, 123182 Moscow, Russia

**Keywords:** plant genomics, hardy kiwi, RAD sequencing in plants, SNP markers in plants

## Abstract

More than ten species of the *Actinidia* Lindl. genus bear edible fruits rich in biologically active compounds, which are essential and beneficial for human health. The most popular cultivars today are the large-fruited *Actinidia* species, *A. deliciosa* and *A. chinensis*, commonly known as kiwi. However, small-fruited kiwi cultivars are gaining prominence due to their high nutritional value, superior cold resistance, and suitability for temperate climates. In Russia, these are represented by Far Eastern species: *A. arguta*, *A. kolomikta*, and *A. polygama*. Despite increasing consumer interest, Russian *Actinidia* cultivars remain little studied, with fragmented genetic data available for breeding purposes. Our objective was to analyze the *Actinidia* collection at the Federal Horticultural Center for Breeding, Agrotechnology, and Nursery and the N.V. Tsitsin Main Botanical Garden (MBG RAS, Moscow), which includes samples from four species, *A. kolomikta*, *A. arguta*, *A. polygama*, *A. purpurea*, interspecific hybrids, and derived varieties, using RAD sequencing. We assessed the genetic variability of all species, identified population groups within *A. kolomikta* and *A. arguta* based on origin, determined ploidy levels across the collection, and identified a set of SNP markers associated with valuable agronomic traits.

## 1. Introduction

The genus *Actinidia* Lindl. belongs to the *Actinidiaceae* family within the order Ericales and comprises approximately 55 species of perennial, deciduous, or semi-deciduous climbing lianas [1]. The primary center of origin is considered to be East Asia. Most species diversity is found in present-day China, extending to the Russian Far East, the Korean Peninsula, and Japan [2]. Several *Actinidia* species have been utilized in traditional Chinese medicine for thousands of years [3] due to their wide range of biologically active compounds (BACs) that treat various ailments.

About fifteen species of *Actinidia* produce edible fruits [4] that are notable for their high content of valuable nutrients, vitamins, antioxidants, and other bioactive compounds essential to the human diet, which makes them of significant commercial interest. Moreover, *Actinidia* is one of the few plants with a very short domestication history, with the first representatives of this genus being cultivated in the early 20th century. The most well-known species to consumers are the large-fruited *A. deliciosa* C.F. Liang & A.R. Ferguson and *A. chinensis* Planch., commonly referred to as kiwi. In 1904, kiwi seeds were first introduced to New Zealand [5], and since then, the industry has rapidly developed, with the fruits now being widely distributed and easily available throughout the year. Today, they are grown in many countries, including Italy, China, New Zealand, Chile, France, Greece, and Japan [6]. However, these species are not frost-hardy and are cultivated only in temperate subtropical regions. In colder areas, they require special care to survive winter temperatures below 0 °C.

Small-fruited actinidias are also rich in valuable nutrients, characterized by high levels of organic acids and vitamins, and are low in calories [7,8,9]. Unlike the hairy large fruits, they have a thin, tender skin. The species *A. arguta* Miq. and *A. kolomikta* Maxim. are frost-hardy, making them suitable for cultivation in countries with harsher winters [1]. According to some authors [10], these plants can withstand temperatures as low as −38 °C. Recently, the economic significance of small-fruited kiwi has been increasing worldwide, with annual production reaching about 1600 tons, mainly in the USA, Chile, China, and some European countries [11,12], including regions with cold climates (such as Northern European countries).

In the Russian Far East, five species of *Actinidia* grow: *A. arguta* (Siebold et Zucc.) Planch. ex Miq., *A. polygama* (Siebold et Zucc.) Miq., *A. giraldii* Diels, *A. kolomikta* (Rupr. et Maxim.) Maxim., and *A. purpurea* Rehder [13]. Many taxonomists note the complexity of *Actinidia* taxonomy and the difficulty in determining the phylogenetic relationships among species due to the variability in morphological traits, the blurriness of species boundaries, and the presence of hybrids [14,15,16]. The taxonomic status of *A. giraldii* and *A. purpurea* remains uncertain. In some classifications, *A. giraldii* is considered a synonym of *A. arguta* or its variant var. *giraldii* (Diels) Vorosch., while *A. purpurea* has also been proposed as a variety of *A. arguta*. The most extensive collection in Russia is the *Actinidia* collection curated by E. I. Kolbasina at the Moscow branch of VIR. This collection includes both expedition samples from the Russian Far East and cultivars developed from them through domestic and international breeding programs [17]. Currently, the collection is managed by the Main Botanical Garden of the Russian Academy of Sciences, where biotechnological and genetic research continues [18].

*Actinidia* is an intriguing and underexplored genus both botanically and genetically. Species of the genus have a heterogeneous ploidy structure, with natural occurrences of di-, tri-, tetra-, hexa-, and even octoploids. As more specimens are studied, it becomes apparent that polyploidy is a common feature rather than an exception among actinidias. The basic chromosome number of *Actinidia* is 29, and there is growing evidence [16] suggesting *Actinidia* species are cryptic polyploids or rediploidized paleopolyploids [19]. However, the relationship between the ploidy and agronomic traits of crops is more complex and less straightforward than previously thought. For many crops, significant trait improvements have been observed with increased ploidy [20], though polyploidy also has several drawbacks. Crossbreeding taxa with different ploidy levels can be challenging, and triploid offspring are often sterile, while those with higher ploidy levels may be only partially fertile. For example, in *A. chinensis* [21], it has been shown that polyploid plants can produce numerically unreduced gametes, and such offspring do not always exhibit the expected ploidy.

The collection of Far Eastern *Actinidia* assembled by E. I. Kolbasina represents a unique gene pool of frost-hardy small-fruited kiwi. The description and analysis of the genetic structure of this collection are crucial for biodiversity conservation, further breeding work, and the creation of new cultivars with improved qualities for the market, providing fruits rich in valuable nutrients. Previous studies based on this collection have focused on morphogenetic processes, developing and refining clonal micropropagation techniques, and selecting optimal conditions for the long-term storage of *Actinidia* meristems in an in vitro genetic bank [18]. For some species and cultivars, genome polymorphism has been studied using multilocus RAPD analysis [22]. In our work, we used SNP markers for analyzing the genetic structure of the collection, which offer high-resolution capabilities for the detailed characterization, assessment of variability, and genotyping of existing cultivars. We applied restriction site-associated DNA sequencing (RAD sequencing), a modern genotype analysis method with the advantages of next-generation sequencing (NGS) technology, suitable for population studies at a relatively low cost [23]. Several modifications of this method have been developed to date, including ddRAD sequencing, which allows for the multiplexing of large sample sets by incorporating a four-index sequence step [24].

The aim of this study was to provide a genetic characterization of the collection of Far Eastern *Actinidia* species and cultivars successfully introduced in the Moscow region. This collection serves as the basis for active breeding efforts, and a genetic description of the available samples is necessary to develop new cultivars with promising traits for large-scale cultivation, characterized by enhanced properties.

## 2. Results

### 2.1. Sequencing Data Analysis

Sequencing was conducted on 94 samples of Far Eastern *Actinidia*. The total volume of sequencing data amounted to 678,733,750 paired reads, averaging 7,220,571 reads per sample (ranging from 258,252 to 12,216,737). In total, 530,566,172 reads (78.17%) were mapped to the reference genome of *A. eriantha* after quality filtering. The mapping percentage per sample ranged from 61.58% to 83.56%. The total number of variable loci was 1,084,265. After filtering based on DP and GQ criteria, 46,453 variable loci, each present in at least 95% of the samples, were retained for downstream analyses.

### 2.2. Ploidy Level

The ploidy level of *Actinidia* plants varies significantly. Recent studies indicate the necessity of verifying the ploidy of individual samples rather than assuming it based on taxonomy [25], as ploidy levels in *A. arguta* range from 2× to 10×. The ploidy of the plants in this collection is unknown, so we attempted to determine it for all 94 samples. Using allele heterozygosity depth distributions from genetic analysis, as previously described [25], we showed that all *A. kolomikta* and *A. polygama* samples in this collection are diploid (Supplementary Figure S1). Among *A. arguta*, 17 plants are likely tetraploid, and 7 plants and *A. purpurea* were classified as diploid. Hybrid *A. arguta* × *A. purpurea* plants were found to be either tetraploid (six samples) or diploid (three samples), and *A. arguta* var. *giraldii* variants were classified as diploid.

### 2.3. Genetic Structure of the Far Eastern Actinidia Collection

**Figure 1:**
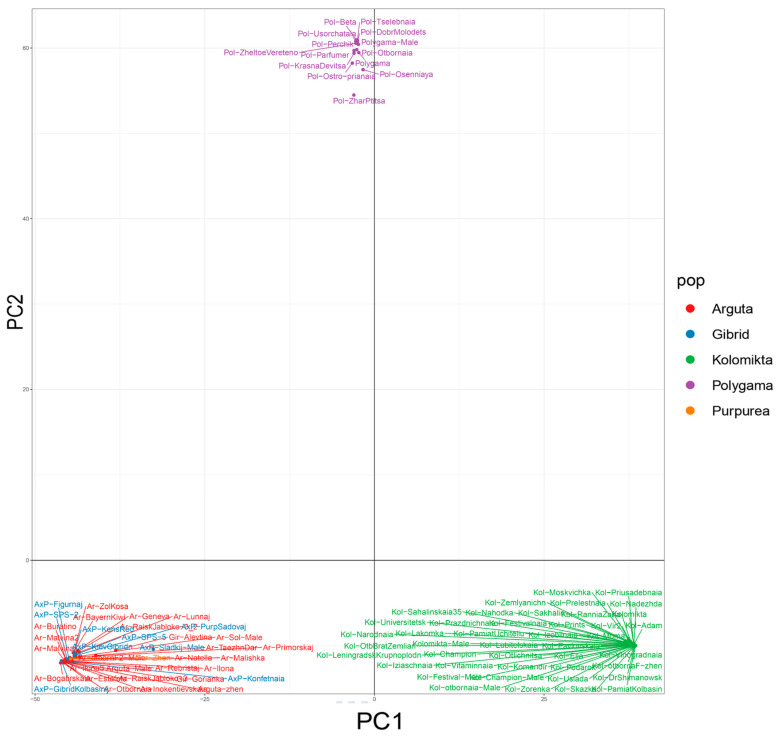
PCA of 94 *Actinidia* varieties. Groups are marked with different colors. PCA analysis revealed three distinct species-specific groups. The first and second clusters consisted of *A. kolomikta* and *A. polygama*, while the third cluster included all *A. arguta* cultivars, variants, and hybrids and *A. purpurea*.

Principal Component Analysis (PCA) was used to assess the genetic diversity structure within the *Actinidia* collection, and the results are shown in Figure 1.

**Figure 2:**
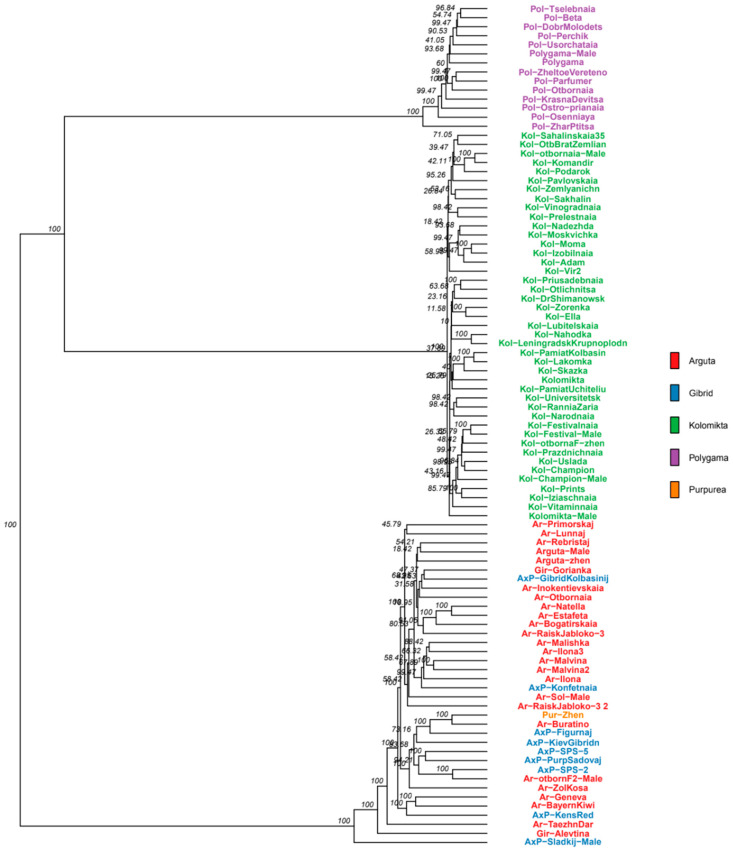
Dendrogram of 94 *Actinidia* varieties constructed using the UPGMA method based on 82,777 SNPs obtained via RAD sequencing. Branch nodes display bootstrap indices (in %). Groups are marked with different colors..

The genetic relationships among the 94 *Actinidia* samples were evaluated using a phylogenetic tree. We performed cluster analysis based on Euclidean distances, using a filtered SNP dataset obtained from RAD sequencing and applying the UPGMA method, as illustrated in Figure 2.

The resulting dendrogram reflects a population structure similar to that obtained through PCA, confirming a clear differentiation among the three species and their cultivar derivatives. The *A. kolomikta* and *A. polygama* species appeared more closely related, unlike *A. arguta*, whose representatives formed a separate cluster.

Fixation indices (Fsts) were determined for the three *Actinidia* species we studied, as well as *A. purpurea* (Table 1). The fixation index (Fst) assesses how distinct populations are from each other, with high values indicating significant differentiation. In our case, the Fst values between the three species were high, while a low fixation index (0.018) was observed between *A. arguta* and the taxonomically ambiguous *A. purpurea*.

**Figure 3:**
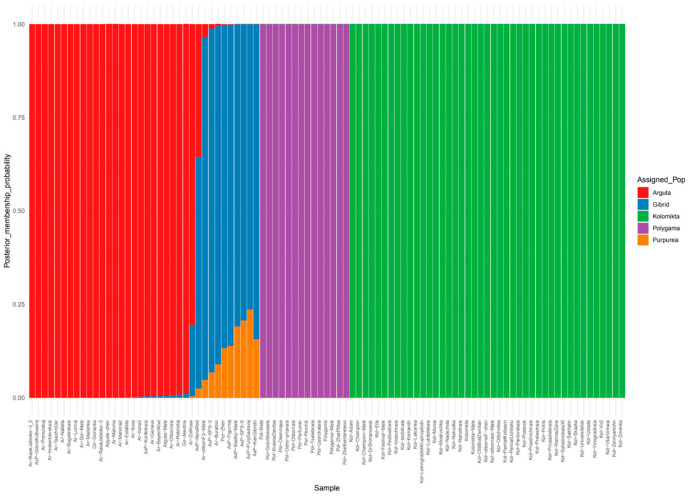
Barplot describing the intraspecific genetic structure of the Far Eastern Actinidia collection using the discriminant analysis of principal component method. The population was divided into five clusters (K = 5) based on the most informative K value using the Bayesian information criterion.

We also analyzed the genetic structure of the *Actinidia* samples using the discriminant analysis of principal components. The optimal number of subgroups best explaining the genetic structure of each species is considered the smallest K value before an increase in BIC (Supplementary Figure S2). Thus, the value 5 was chosen for this population (Figure 3).

The *A. kolomikta* and *A. polygama* populations formed distinct groups, while the *A. arguta* population divided into several groups. All *A. arguta* representatives, including two samples of *A. arguta* var. *giraldii* marked in red, were found to have a genotype without impurities or with an insignificant amount of them.

The other two groups were the *A. purpurea* and *A. arguta* × *A. purpurea* hybrid samples, which formed a mixed group on the barplot, consisting of admixed genotypes.

### 2.4. Intraspecific Genetic Structure of the Far Eastern Actinidia Collection

The genetic diversity within the three *Actinidia* species populations was quantified using various criteria, including observed heterozygosity (Ho), genetic diversity within populations (Hs), and the inbreeding coefficient (FIS). The results are presented in Table 2.

Among the three populations studied, the highest heterozygosity and genetic diversity were observed in the *A. arguta* population, while *A. kolomikta* had the highest FIS values.

A Bayesian information criterion (BIC) analysis for investigating intraspecific genetic structure revealed two subpopulations within *A. kolomikta* and *A. arguta*. The *A. polygama* population appeared homogeneous and did not split into groups based on K-means analysis (Supplementary Figures S3–S5).

**Figure 4:**
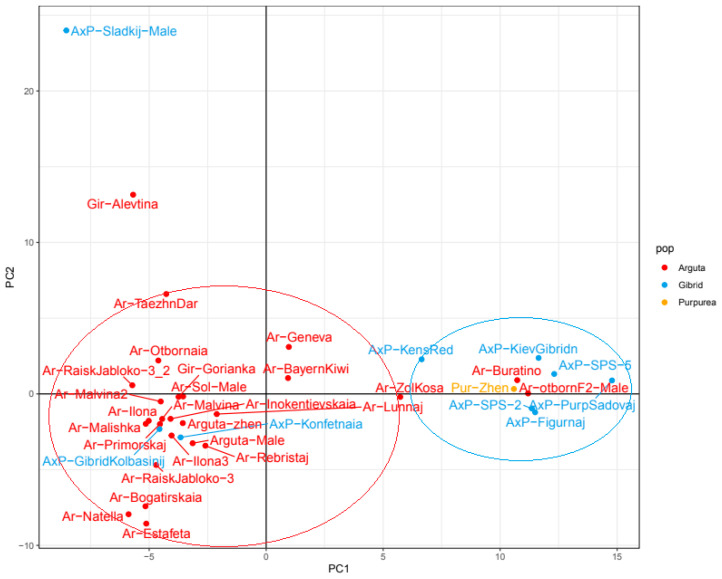
PCA of *A. arguta* group cultivars. The red oval highlights the group of *A. arguta* cultivars, and the blue oval highlights the group of hybrids *A. arguta* × *A. purpurea* and *A. purpurea*.

A further PCA analysis of *A. kolomikta* and *A. arguta* populations showed that *A. arguta* also split into two main groups (Figure 4). One group was composed mainly of *A. arguta* variants, while the other consisted of *A. arguta* × *A. purpurea* hybrids and *A. purpurea* itself.

**Figure 5:**
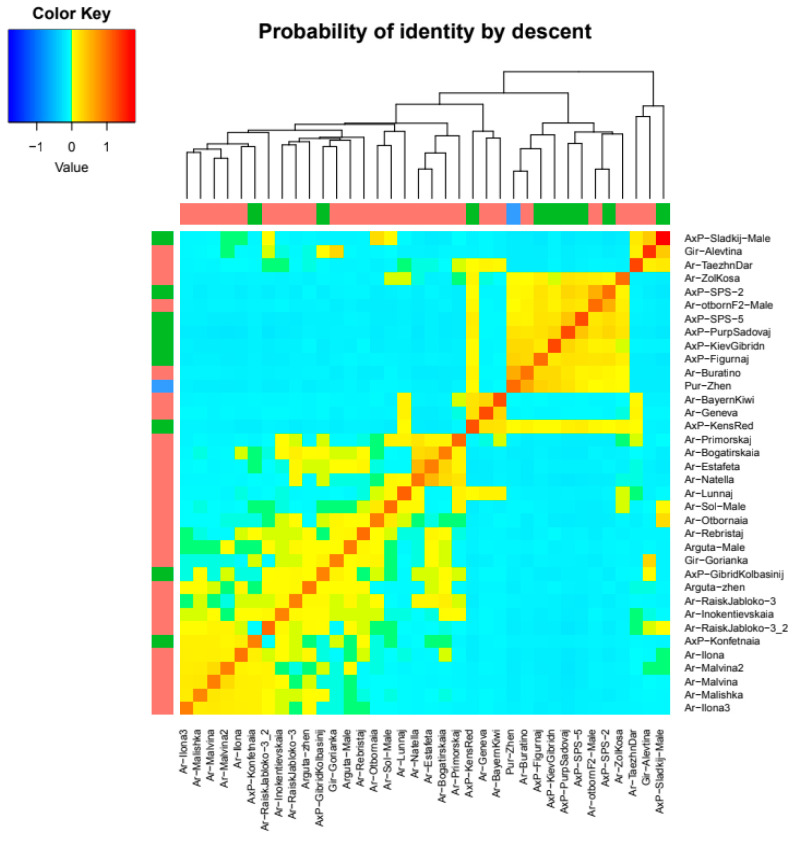
Heat map illustrating genetic relatedness within the *A. arguta* population, derived from paired identity-by-descent (IBD) coefficient values. Warmer colors (red) indicate closer genetic relationships, while cooler colors (blue) represent more distant genetic connections.

**Figure 6:**
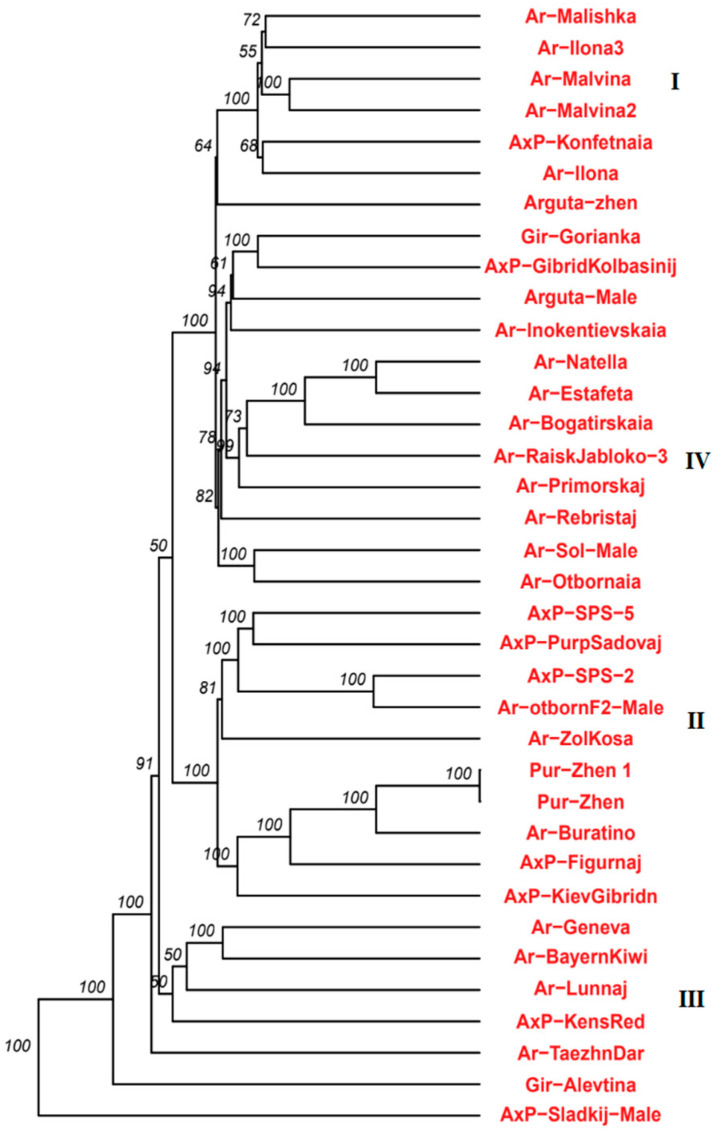
Cladogram showing relationships within the *A. arguta* population. The numbers indicate groups within the population.

Based on the IBD coefficient, which evaluates the number of shared identity-by-descent alleles in pairwise sample comparisons from the *A. arguta* population, heat maps and a cladogram illustrating intrapopulation relationships were constructed (Figure 5 and Figure 6, Supplementary Table S1).

The subpopulation of hybrid samples and *A. purpurea* formed a distinct group marked as II on the cladogram. In the group containing primarily *A. arguta* samples (marked as III), a separate cluster comprised three foreign-bred cultivars: *A. arguta* Bayern Kiwi (Germany), *A. arguta* Geneva (USA), and the hybrid cultivar Ken’s Red (New Zealand). Samples obtained from free pollination from one female plant are united under the common name “family”. On the cladogram, representatives of two close “families” formed a separate cluster (designated by number I). *A. arguta* Malishka, *A. arguta* Malvina, and *A. arguta* Malvina 2 belong to “family” 55, while *A. arguta* Ilona and *A. arguta* Ilona 3 are “family” 66. Three heterogeneous samples, *A. arguta* Taezhnii Dar, the hybrid *A. arguta x A. purpurea* Sladkij, and *A. arguta* var. *giraldii* Alevtina, formed basal branches in the species clade based on both IBD coefficient analysis and phylogenetic data.

**Figure 7:**
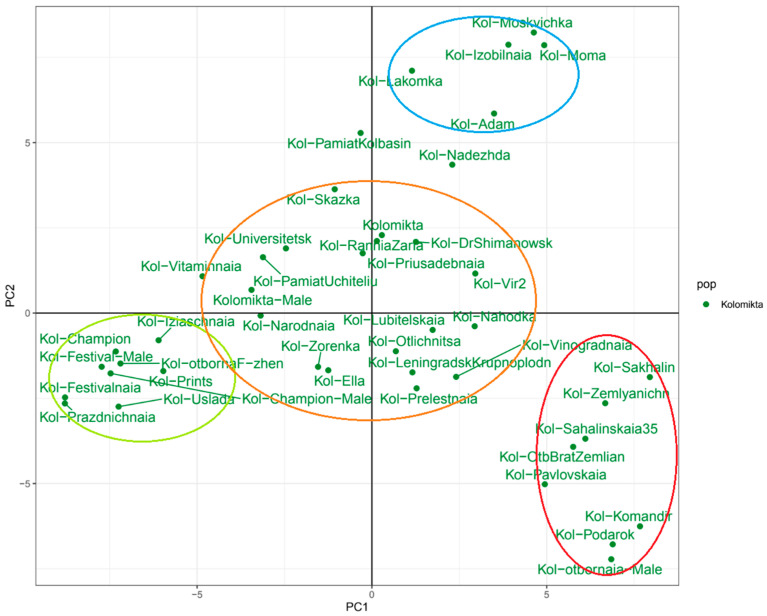
PCA of *A. kolomikta* varieties. The four groups into which this population is divided are highlighted in color.

The PCA results indicated that the *A. kolomikta* population was divided into four distinct groups (Figure 7). The blue group consisted of samples from the open pollination of a single female plant, characterized by non-dropping fruits, while the rest of the *A. kolomikta* representatives had fruit that dropped upon ripening. The green group represented variants developed in the Moscow region, noted for their high ascorbic acid content (Supplementary Table S2). The red group comprised samples introduced to the Moscow region from various areas: the Leningrad and Sakhalin regions, Primorsky Krai, and Dnipropetrovsk Oblast, Ukraine. The most numerous group (orange) contained samples bred both in the Moscow region and sourced from other regions.

**Figure 8:**
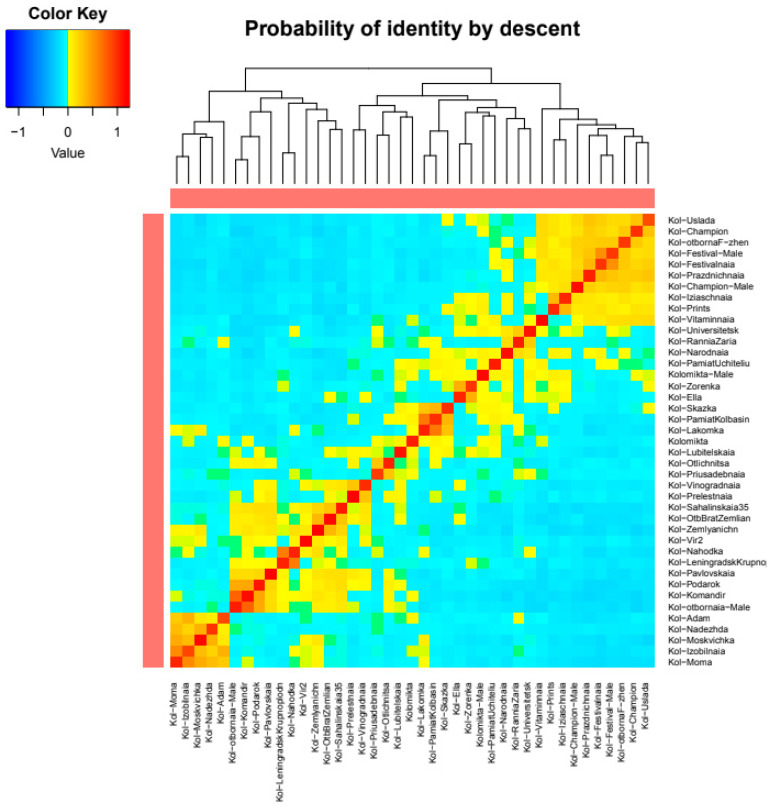
Heat map illustrating genetic relatedness within the *A. kolomikta* population, derived from paired identity-by-descent (IBD) coefficient values. Warmer colors (red) indicate closer genetic relationships, while cooler colors (blue) represent more distant genetic connections.

An IBD-based heat map for *A. kolomikta* samples (Supplementary Table S3) is presented in Figure 8. The grouping largely aligns with the PCA results. The samples *A. kolomikta* Moma, *A. kolomikta* Moskvichka, *A. kolomikta* Nadezhda, and *A. kolomikta* Izobilnaja, obtained from free pollination from one mother plant and with differing non-dropping fruits, show the highest similarity, indicating a relatively homogeneous natural pollinator population and low genetic diversity. Varieties bred in the Moscow region exhibited a high degree of relatedness and shared identity-by-descent alleles based on IBD analysis.

### 2.5. A. kolomikta Population with Non-Dropping Fruits

All *A. kolomikta* representatives had fruit that dropped upon ripening, but in the process of creating a collection four samples were discovered, called *A. kolomikta* Moma, *A. kolomikta* Moskvichka, *A. kolomikta* Nadezhda, *A. kolomikta* Izobilnaja, obtained from the natural population from free pollination from one mother plant and with differing non-dropping fruits. This trait seems interesting to study, and we decided to analyze the genomes of these two subpopulations of *Actinidia kolomikta*.

In this way, subpopulations with dropping and non-dropping fruits were examined using discriminant analysis of principal components (DAPC), which identified 73 SNPs that distinguish these two groups (Supplementary Table S4). The summarized results for the areas of SNP location are presented in Table 3. It was found that some genes contain several SNPs. For instance, the gene encoding E3 ubiquitin-protein ligase BIG BROTHER-like had nine SNPs, the genes for probable leucine-rich repeat receptor-like protein kinase IMK3 and LIM domain-containing protein WLIM2b-like each had three SNPs, and the gene for cysteine-rich receptor-like protein kinase 44 contained four SNPs. Additionally, single SNPs were identified in genes coding for acetylornithine deacetylase-like protein, FAR1-RELATED SEQUENCE 5-like protein, probable protein S-acyltransferase 19, ABC transporter B family member 19, and ethylene-responsive transcription factor 13-like.

## 3. Discussion

Molecular markers are essential for studying genetic diversity and population structure, identifying cultivars, developing targeted breeding strategies, finding genomic associations, and addressing various genetic and breeding challenges in plants. The development of next-generation sequencing technologies has made single-nucleotide polymorphisms (SNPs) highly useful as markers, as their analysis offers many advantages in genotyping due to their high resolution. Genomic sequencing methods of DNA fragments associated with restriction sites (RAD tags) are widely used for SNP discovery in plants because of the simplified library preparation technology and cost-effectiveness of sequencing [26,27,28,29].

Collections of *Actinidia* in various countries are gathered and studied using molecular markers [25,30,31,32]. We analyzed the genetic diversity and structure of the Far Eastern *Actinidia* collection at the N.V. Tsitsin Main Botanical Garden. SNP marker analysis revealed a clear division into species-specific groups. According to our phylogenetic analysis, *A. kolomikta* and *A. polygama* cluster together, in contrast to *A. arguta*. This aligns with the results obtained from the GBS analysis of the American germplasm collection [25], while RAPD analysis results [22] showed *A. polygama* and *A. arguta* clustering together, with *A. kolomikta* forming a separate branch.

The studied collection samples were divided into three subpopulations corresponding to the three species: *A. arguta*, *A. kolomikta*, and *A. polygama*. *A. giraldii* did not form a separate cluster and grouped with the *A. arguta* cultivars, suggesting that it may be a variety of *A. arguta*, which is consistent with the data of other authors [33]. *A. purpurea* and its hybrids formed a distinct group within the *A. arguta* cluster, with the species sample of *A. purpurea* indicating a hybrid nature. Previously, *A. purpurea* was described as a separate species [22], but later it was considered as a variety of *A. arguta*. It is difficult to determine its taxonomic status, since despite the closeness of these two species, there are quite a few characteristics that separate them. Previous studies based on RAPD analysis, the same as in our study, showed *A. purpurea* forming a separate clade on the dendrogram [22]. It is possible that multiple hybridization events occurred between *A. arguta* and *A. purpurea* in natural populations, leading to many transitional forms and the eventual loss of the *A. purpurea* species.

The standard fixation index scale, established by Hartl and Clark and Frankham et al. [34,35], defines Fst values of <0.05 as indicating negligible differentiation, Fst = 0.05–0.15 as moderate genetic differentiation, and Fst > 0.15 as substantial genetic differentiation. In our case, a low fixation index (0.018) was observed between *A. arguta* and the taxonomically ambiguous *A. purpurea*, indicating minimal differentiation between them.

The *A. arguta* population was characterized by the highest heterozygosity (Ho) and genetic diversity coefficients compared to *A. kolomikta* and *A. polygama*, suggesting heterogeneity within the population. Recent studies of a large population of *A. arguta* in China have noted a division into two major subgroups, Northern and Southern, based on their origin. As the two groups diverged further, *A. arguta* formed more phylogeographic subgroups, in which the Northern group showed higher genetic differentiation than the Southern group [36]. *A. kolomikta* had the highest inbreeding coefficient (FIS) among the three species, explained by the presence of samples from several “families” or offspring from the open pollination of a single mother plant.

The *A. arguta* group demonstrated the most genetic diversity, with several subpopulations of *A. arguta* and its hybrids evident in the sample, as reflected by different analysis methods. PCA analysis split the *A. arguta* population into two groups: one comprising hybrid samples and the species sample of *A. purpurea*, and the other consisting of *A. arguta* samples. The IBD-based cladogram identified distinct “families” within the *A. arguta* group characterized by a common ancestry. Some sample placements in specific clusters were unexpected, such as *A. arguta* Buratino and *A. arguta* Otbornaja F2 appearing among the hybrid group. This could be due to these samples being male plants, making their identification challenging without fruit, potentially resulting in misidentification. Developing genetic markers to distinguish these two groups could prevent such issues in the future.

The hybrid group of *A. arguta* and *A. purpurea* is of particular interest. Studying hybrid forms is crucial for developing cultivars with red and purple fruits containing valuable bioactive compounds such as cyanidin and delphinidin. Cyanidin derivatives have been found in all anthocyanin-containing *Actinidia* species, while delphinidin derivatives have only been identified in two taxa: *A. arguta* var. *purpurea* and *A. melanandra* [37]. Moreover, *A. arguta* plants are highly frost-resistant, so hybrid forms combining frost tolerance with valuable bioactive compounds have significant breeding potential. The hybrid forms in our sample produce fruits in various shades of red, purple, and violet, are highly productive, and have excellent organoleptic properties, making them highly valuable for future breeding efforts.

The *A. kolomikta* population, as shown in the phylogenetic tree and IBD analysis, separated into groups corresponding to their origins. The study also included representatives that belonged to “families” with the best agronomic traits. These plants formed common clusters on the phylogenetic tree, with some families displaying unique characteristics, such as a high ascorbic acid content, large fruit size, and non-dropping fruits. Some *A. kolomikta* samples were found to have an exceptionally high vitamin C content in their fruits (1000–2000 mg per 100 g of raw mass) [38].

Among the promising families, one stood out for having non-dropping fruits, a trait unusual for *A. kolomikta*. Fruit drop is a universal process where various plant organs are shed during normal development or in response to tissue damage and stress. This occurs through cell separation in regions known as abscission zones (AZs). Ethylene is a key regulator of organ abscission. Genes encoding ethylene-responsive transcription factors (ERFs) are involved in the ethylene signaling pathway and play a crucial role in plant responses to biotic and abiotic stresses [39]. Literature reports indicate that the overexpression of ERF family genes accelerates cell separation in the AZ, promoting fruit abscission in transgenic tomato lines and *Acacia catechu*. In our study, an SNP was identified in the gene encoding ethylene-responsive transcription factor 13-like. Cell separation in the AZ involves the degradation of pectic polysaccharides, catalyzed by enzymes such as polygalacturonase (PG), pectin methylesterase (PME), pectin lyase (PL), and β-galactosidase (BGLA), making these genes crucial for organ abscission [40]. However, we did not find SNPs in these genes that differentiated the plants with dropping versus non-dropping fruits.

Organ abscission is often associated with aging. Certain proteins in the S-acyltransferase (PAT) group have been shown to play roles in leaf senescence control [41]. Three classes of PAT positively influence brassinosteroid (BR) hormone signaling, which is essential for cell growth and division. PAT19, PAT20, and PAT22 are constitutively expressed, with their protein products linked to the plasma membrane and trans-Golgi network/early endosomes [41]. When comparing populations with dropping and non-dropping fruits, we identified an SNP in the gene encoding probable protein S-acyltransferase 19.

SNPs were also found in genes associated with plant responses to various stresses. For instance, the acetylornithine deacetylase-like protein gene is linked to salt stress in plants [42,43], while the protein FAR1-RELATED SEQUENCE 5-like is involved in abiotic stress response [44]. Genes from the ABC transporter B family play crucial roles in the transmembrane distribution of various molecules, aiding in the adaptation to rapidly changing conditions like water scarcity, heavy metal stress, and pathogen attacks [45].

Leucine-rich repeat receptor-like protein kinase (LRR-RK) genes participate in a wide range of signaling pathways and have been linked to plant responses to biotic and abiotic stress [46,47]. The LRR-RK subfamily is crucial for regulating cell division, organ morphology, inflorescence composition [48], and coordinating cell elongation [49].

LIM domain-containing protein WLIM2b-like has been shown to play a role in fiber elongation and secondary cell wall synthesis [50]. In our study, we found three SNPs in the gene encoding LIM domain-containing protein WLIM2b-like. Additionally, nine SNPs were identified in the gene encoding E3 ubiquitin-protein ligase BIG BROTHER-like (BB), a central organ size regulator. Literature reports suggest an inverse correlation between BB expression and organ size [51]. BB limits the cell proliferation phase, causing cells to divide more slowly and elongate later. It also participates in cell wall formation [52].

In our research, we attempted to determine the ploidy levels of the collection using bioinformatic tools. *Actinidia* species exhibit varying ploidy levels [53,54], which are critical for breeding as differences in ploidy levels among parental forms can complicate successful crossbreeding and hybrid fertility. Polyploid plants are often associated with a higher biomass and superior crop traits, leading to the search for germplasm with higher ploidy levels in agricultural crops [55,56]. However, the relationship between ploidy and agronomic traits may be more complex than it appears [20,57]. Our data suggest that most *A. arguta* samples likely have a tetraploid chromosome set, while seven plants and *A. purpurea* are diploid. Most hybrids were also tetraploid, although two *A. arguta* var. *giraldii* variants were classified as diploid. Assessing the impact of ploidy on the agronomic traits of *Actinidia* in our collection is currently not feasible due to the lack of phenotypic data for some samples. The ploidy data we obtained are preliminary and will be further verified through flow cytometry.

Our work characterized the genotypes in the Far Eastern *Actinidia* collection at the Federal Scientific Center for Horticulture using SNP markers obtained through RAD sequencing. The results of the whole-genome study revealed the population structure of the collection, establishing that *A. kolomikta* and *A. polygama* are more closely related to each other than to *A. arguta*. Specific variants of *A. kolomikta* and *A. arguta* from our collection exhibit high genetic potential, making them suitable for use in breeding crosses to enrich the genetic pool of cultivars and create forms with new traits and trait combinations. One family of *A. kolomikta* is particularly notable for a valuable agricultural trait: non-dropping fruits. We identified several SNPs that distinguish the non-dropping fruit group, which may be linked to the fruit abscission process in *Actinidia*. In addition to *A. arguta*, our collection includes several hybrid forms of *A. purpurea* × *A. arguta*, which are rich in valuable bioactive compounds and, according to our analysis, form a distinct group within the *A. arguta* population.

## 4. Materials and Methods

### 4.1. Plant Material and DNA Extraction

In total, 94 samples of Far Eastern *Actinidia* (*Actinidia* Lindl., family *Actinidiaceae* Van Tiegh.) were collected and analyzed. The analysis included 24 samples of *A. arguta* (Siebold ex Zucc.) Planch. ex Miq., 43 samples of *A. kolomikta* (Rupr. ex Maxim.) Maxim., and 15 samples of *A. polygama* (Siebold ex Zucc.) Maxim. Additionally, 9 hybrid genotypes of *A. arguta x A. purpurea*, as well as 2 variants each of *A. arguta* var. *giraldii* and *A. arguta* var. *purpurea*, were included. A detailed description of all collection samples is provided in Supplementary Table S2.

Young leaves from 2–3 vines of each plant were collected, pooled, and used for DNA extraction following the Lo Piccolo method [58] with slight modifications [59]. The quality and quantity of DNA were assessed spectrophotometrically using a Nanodrop 1000 device (Thermo Scientific, Waltham, MA, USA) and a Qubit fluorometer (Thermo Scientific, Waltham, MA, USA) with the Qubit™ dsDNA BR Assay Kit.

### 4.2. Preparation of RAD Libraries and Sequencing

Genomic DNA from the 94 *Actinidia* samples was digested with the restriction endonucleases MspI and PstI. Adapters, complementary to the restriction sites and containing unique barcodes, were simultaneously ligated at 30 °C for 3 h. The samples were then equimolarly pooled into sets of 12 with various barcode combinations and purified using Agencourt AMPure XP magnetic beads (Beckman Coulter, Brea, CA, USA). The target fragment distribution, ranging from 300 to 700 nucleotides, was collected using a BluePippin device (Sage Science Inc, Beverly, MA, USA). PCR was performed to enrich the libraries and attach external TruSeq indices, followed by purification with AMPure XP beads (Agencourt, Bourgogne-Franche-Comté region, France) [60]. The final libraries were checked using a Qubit 2.0 fluorometer (Invitrogen, Carlsbad, CA, USA) and a DNA bioanalyzer (Agilent Technologies, Santa Clara, CA, USA). The ddRAD libraries were sequenced using an Illumina NovaSeq6000 S1 flow cell (Illumina, San Diego, CA, USA) with paired-end reads (2 × 150 bp) at the National Research Center “Kurchatov Institute” (Moscow, Russia).

### 4.3. Data Analysis

The demultiplexing of reads for the 94 samples was performed using the process_radtags program (Version 2.64) [61], with restriction parameters for MspI and PstI. The quality of the nucleotide sequences was evaluated using FASTQC (https://www.bioinformatics.babraham.ac.uk/projects/fastqc/) (accessed on 1 March 2023: Version 0.12.0). Further filtering of the raw reads for quality and sequence length was performed using Trim Galore (https://www.bioinformatics.babraham.ac.uk/projects/trim_galore/) (accessed on 19 November 2019: Version 0.6.5). The reads were aligned to the *Actinidia eriantha* reference genome (https://www.ncbi.nlm.nih.gov/datasets/genome/GCF_019202715.1/), (accessed on 13 July 2021) and variable loci were identified using bowtie2 [62] and gstacks [61] with default settings. Loci were filtered based on DP > 10 and GQ > 20 parameters using the R package vcfR [63], removing poorly covered and oversaturated loci based on normal distribution metrics. We also filtered out SNPs that were found in less than 90% of samples. Sample clustering was performed by constructing dendrograms using the UPGMA (Unweighted Pair Group Method with Arithmetic Mean) methods with the poppr package [64]. Principal Component Analysis (PCA) was conducted in R using the dartR package [65], which also calculated Fst and IBD statistics. Discriminant analysis of principal components (DAPC) was carried out using the adegenet package [66]. The population structure analysis was performed based on discriminant analysis of principal components, and visualization was achieved using Complot from the adegenet package [67]. To select the optimal parameter K, which describes the number of subpopulations in the overall population, the Bayesian information criterion was used, using the find.cluster function, which implements the clustering procedure used in discriminant analysis of principal components (DAPC) and calculates the statistical measure of goodness of fit (BIC), which allows choosing the optimal K. We annotated the SNPs and searched for associated genes using SNPeff v4.3 [68]. For this purpose, we developed a new database based on the SNPeff annotation of the *Actinidia eriantha* genome (GCF_019202715.1). To determine ploidy, we analyzed allele balance distributions based on coverage from the vcf file, considering only heterozygous variants. The highest allele frequency peaks at a ratio of 1/2 corresponded to diploids, while 1/3 and 2/3 indicated triploids.

## Figures and Tables

**Table 1:** Genetic differentiation among *Actinidia* species based on the Fst statistics.

	*A. arguta*	*A. kolomikta*	*A. polygama*	*A. purpurea*
*A. arguta*	0			
*A. kolomikta*	0.838	0		
*A. polygama*	0.806	0.878	0	
*A. purpurea*	0.018	0.890	0.873	0

**Table 2:** Population parameters characterizing the genetic diversity among and within the three *Actinidia* species studied.

	Ho	Hs	FIS
*A. arguta*	0.0553	0.0631	0.1244
*A. kolomikta*	0.0266	0.0318	0.1642
*A. polygama*	0.0272	0.0311	0.1268

**Table 3:** Distribution of SNPs distinguishing samples of *Actinidia kolomikta* with non-dropping fruits by genome region.

Area of SNP Location	% SNP
intron_varian	16.44%
intragenic_variant	8.22%
missense_variant	5.48%
3_prime_UTR_variant	1.37%
stop_gained	1.37%
downstream_gene_variant	19.18%
upstream_gene_variant	19.18%
synonymous_variant	28.77%

## Data Availability

The raw sequence data were deposited into the National Center for Biotechnology Information (NCBI) repository, and the accession number is PRJNA1188808. The genotype file in vcf format is available at the link https://doi.org/10.6084/m9.figshare.28014884 (accessed on 12 December 2024).

## Supplementary Materials

The following supporting information can be downloaded at: https://www.mdpi.com/article/10.3390/plants14010007/s1, Figure S1: Ploidy-specific distributions of allele depth ratios across heterozygous loci. Diploid (single peak) and tetraploid (three local peaks) genomes are distinguishable based on their distinct patterns of peaks in these plots. Table S1: Samples of *Actinidia* spp. taken for analysis. For each accession, the following information is provided: (1) sample ID; (2) accession name; (3) sample gender; (4) estimated ploidy; (5) ascorbic acid content; (6) sample origin. Figure S2: Bayesian information criterion (BIC) for the number of subgroups within *Actinidia* spp. The optimum number of subgroups that best explains the genetic structure within each species is considered to be the lowest value of K (i.e., minimum K) followed by an increase in BIC value. The value of four was chosen for the analyzed population. Figure S3: Bayesian information criterion (BIC) for the number of subgroups within *Actinidia arguta*. Figure S4: Bayesian information criterion (BIC) for the number of subgroups within *Actinidia kolomikta*. Figure S5: Bayesian information criterion (BIC) for the number of subgroups within *Actinidia polygama*. Table S2: Common allele values of identity by descent (IBD) for all *A. arguta* samples. Table S3: Common allele values of identity by descent (IBD) for all A. *kolomikta* samples. Table S4: List of SNPs distinguishing groups of actinidia with deciduous and non-deciduous fruits.
