# Supplementary material for: Genetic Diversity of the Collection of Far Eastern Actinidia spp. Revealed by RAD Sequencing Technology
Source: Plants (Basel). 2024 Dec 24;14(1):7. doi: 10.3390/plants14010007 (PMC11723124; doi:10.3390/plants14010007)
Supplement: Supplementary file 1 [file plants-14-00007-s001.zip › plants-3356746-supplementary/Supplementary/Figure S1.pdf]

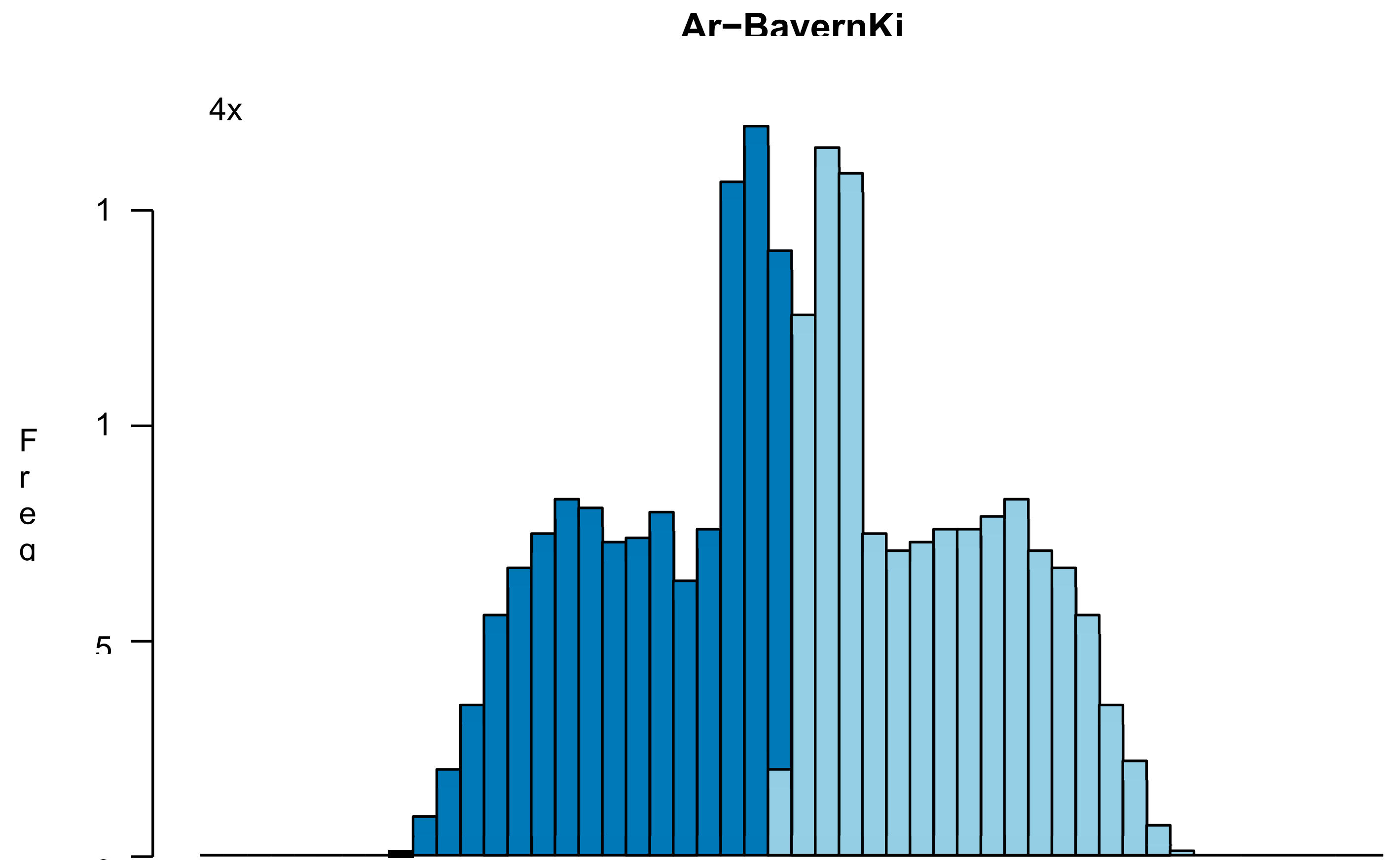
Ar−BavernKi
4x
1
1
F
r
e
a
5

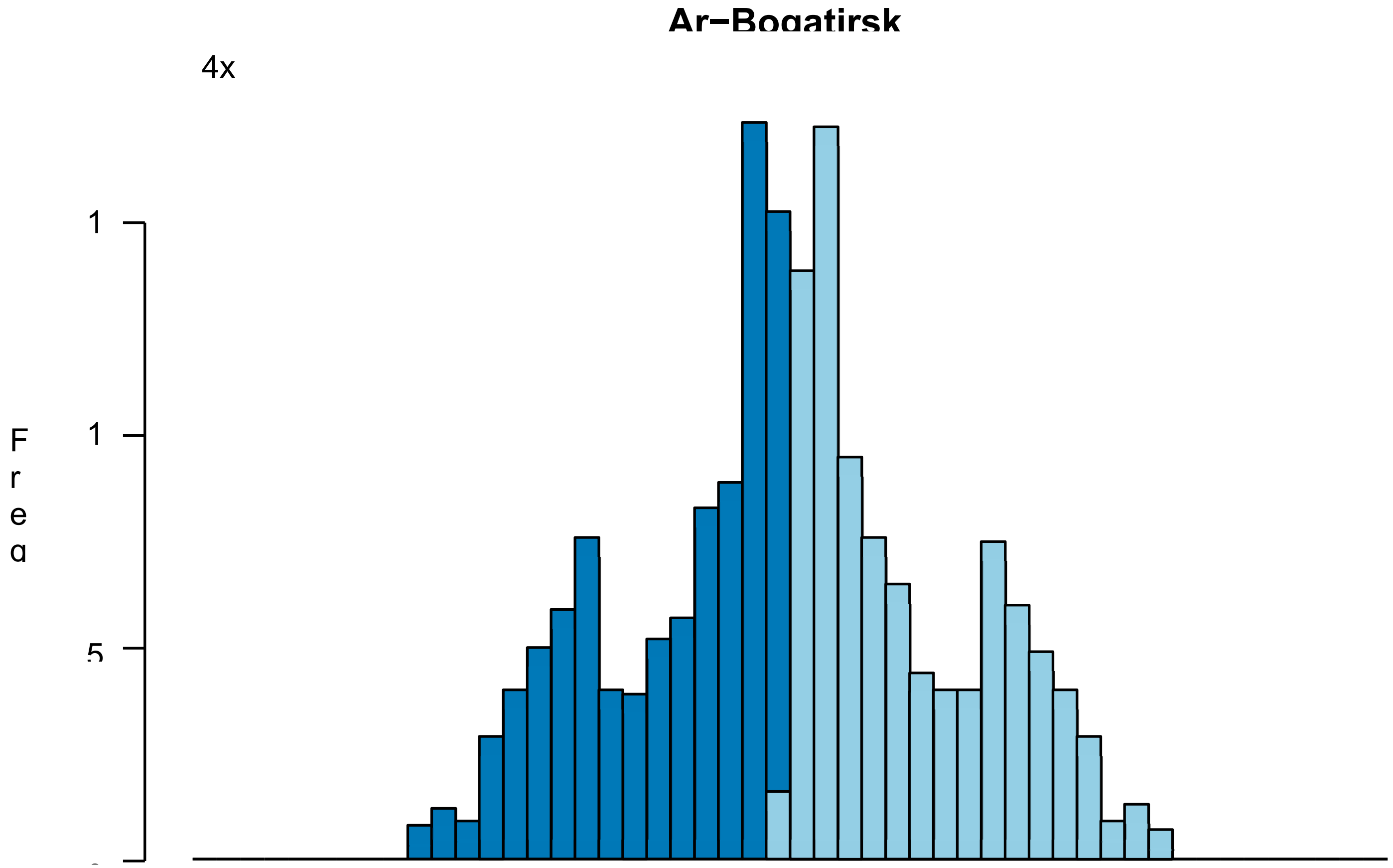
Ar−Bogatirsk
4x
1
1
5
F
r
e
q

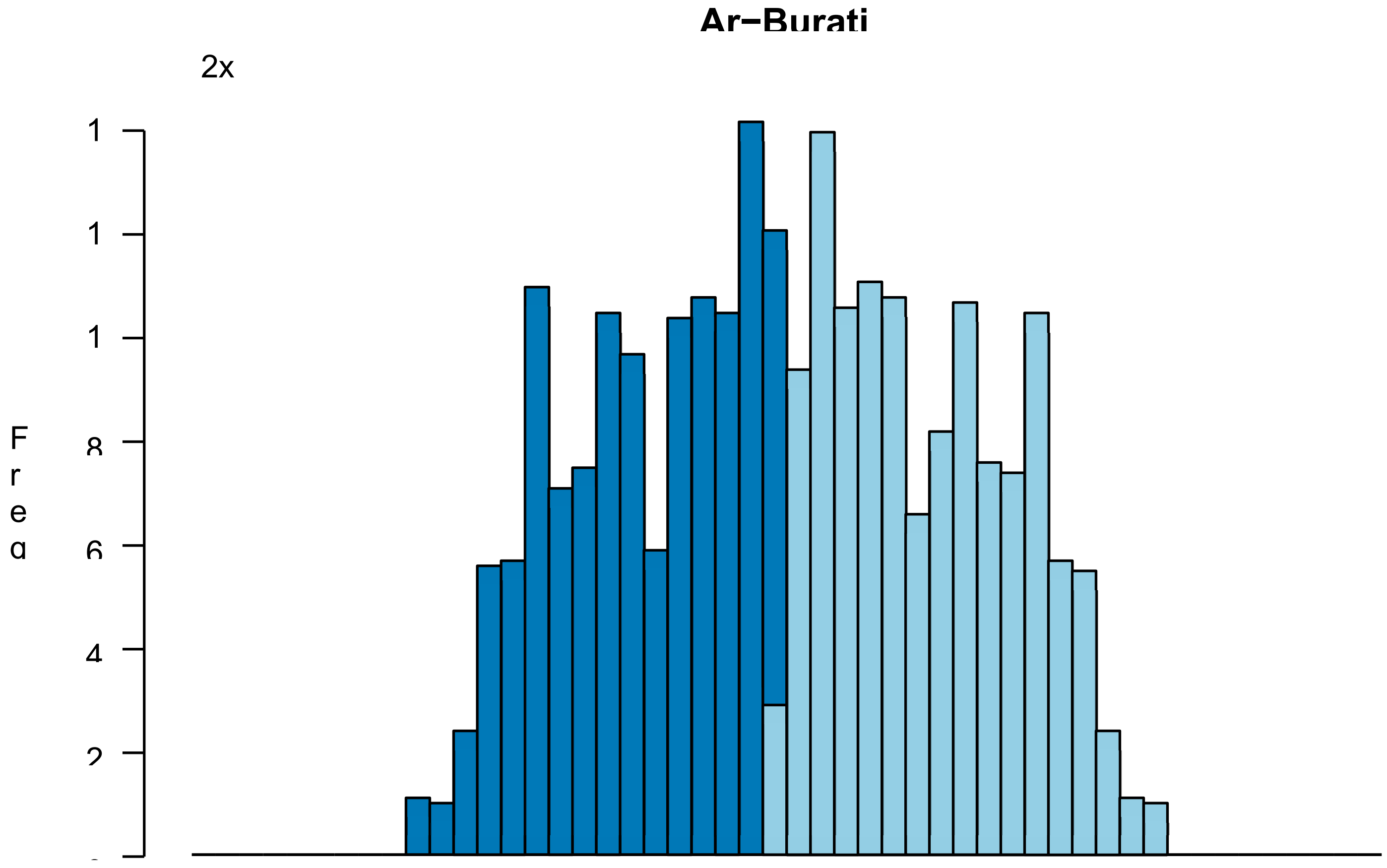
Ar−Burati
2x
1
1
1
8
6
4
2
F
r
e
q

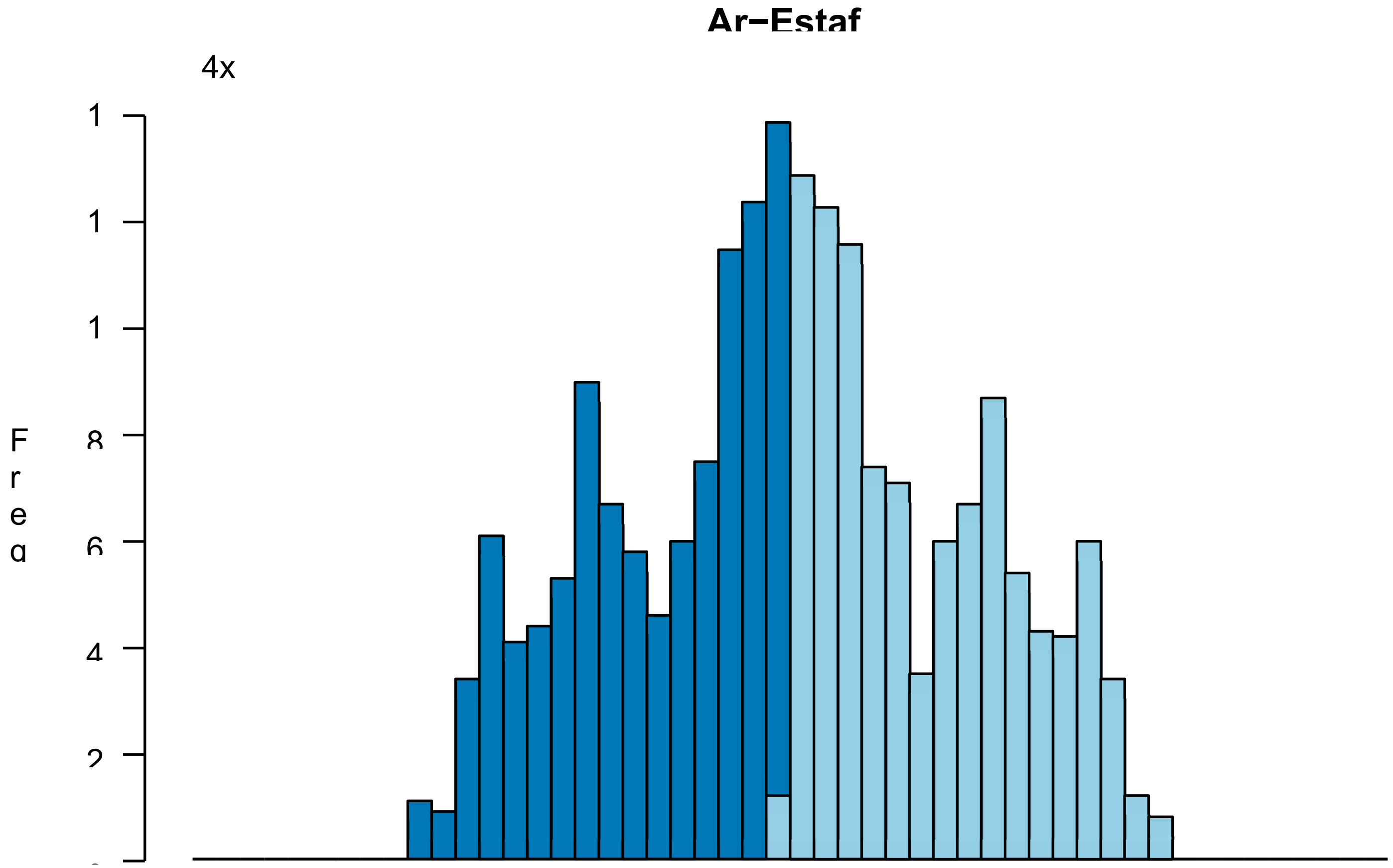
Ar−Estaf
4x
1
1
1
8
6
4
2
F
r
e

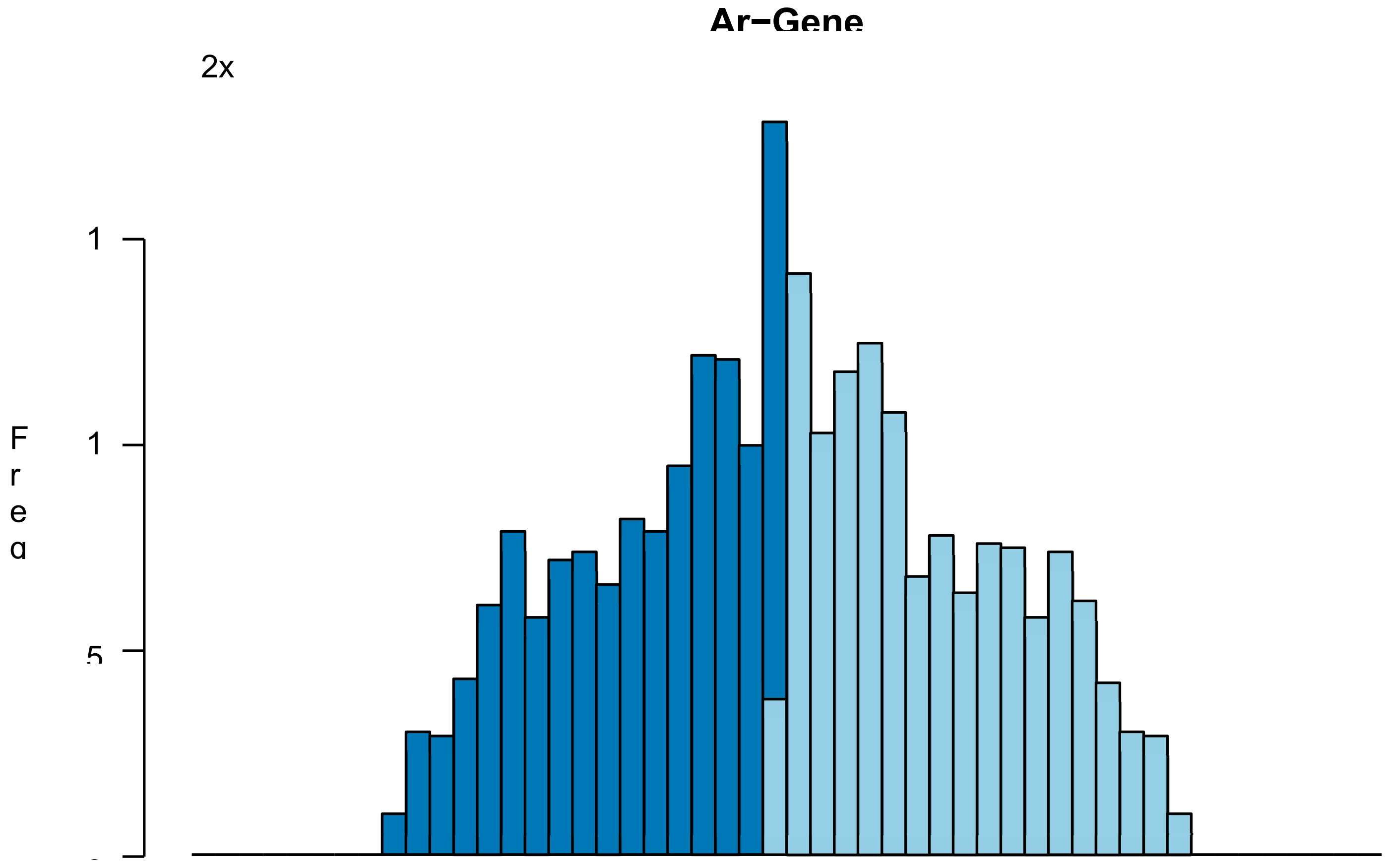

Ar−Gene
2x
1
1
5
F
r
e
q

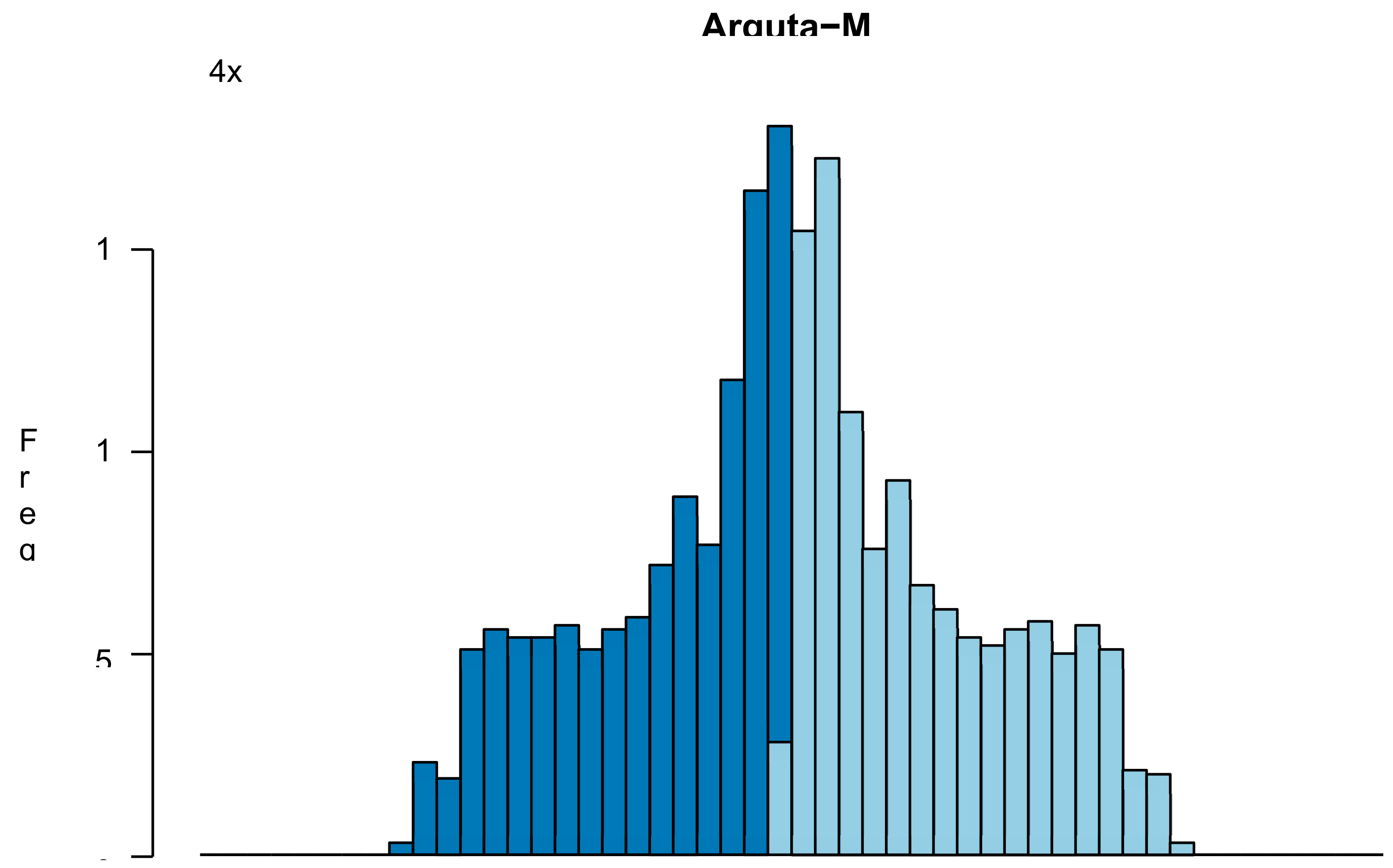
Arguta−M
4x
F
r
e
q
1
1
5

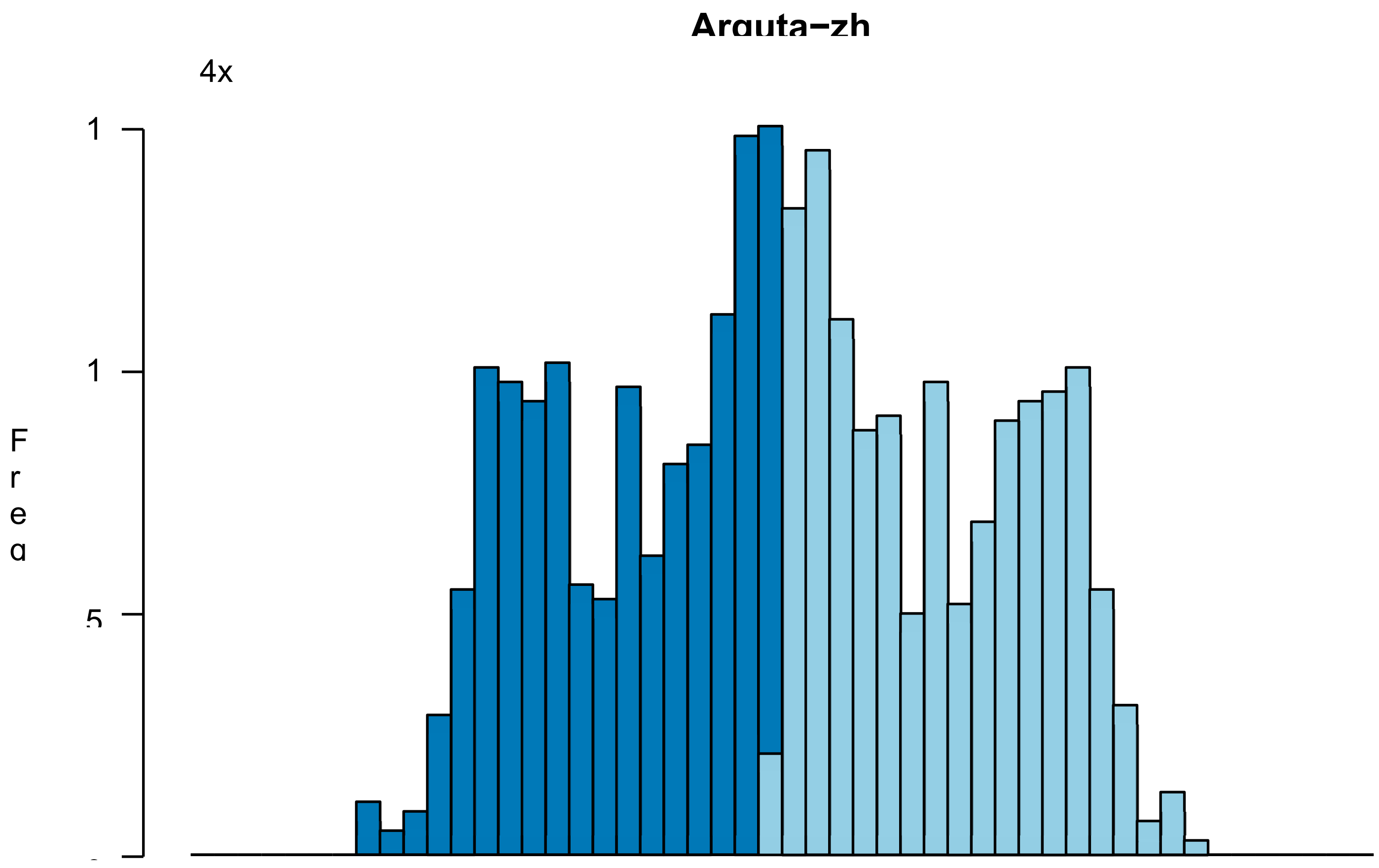
Arguta−zh
4x
1
1
5
F
r
e
q

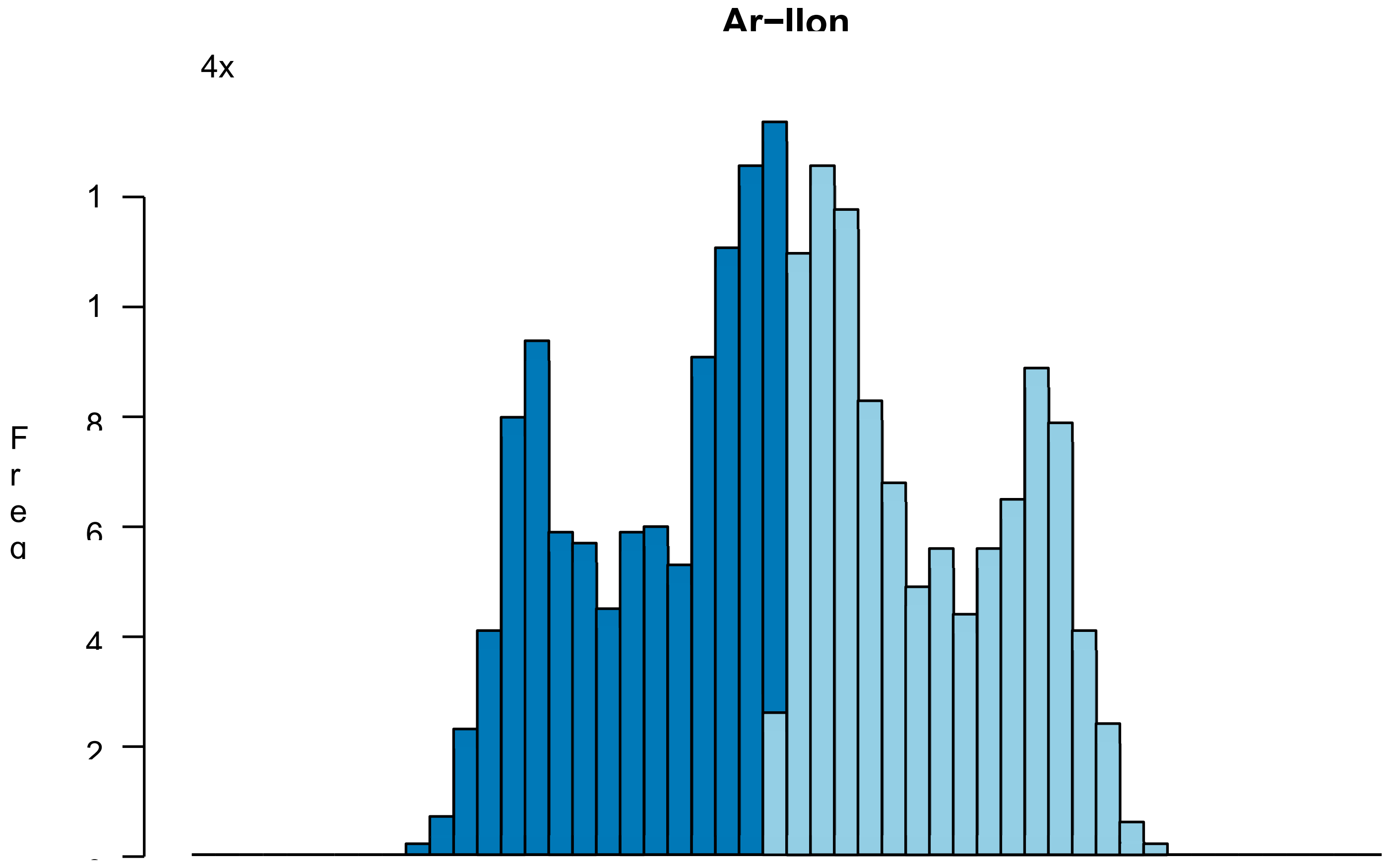

Ar−Ilon
4x
1
1
8
6
4
2
F
r
e
q

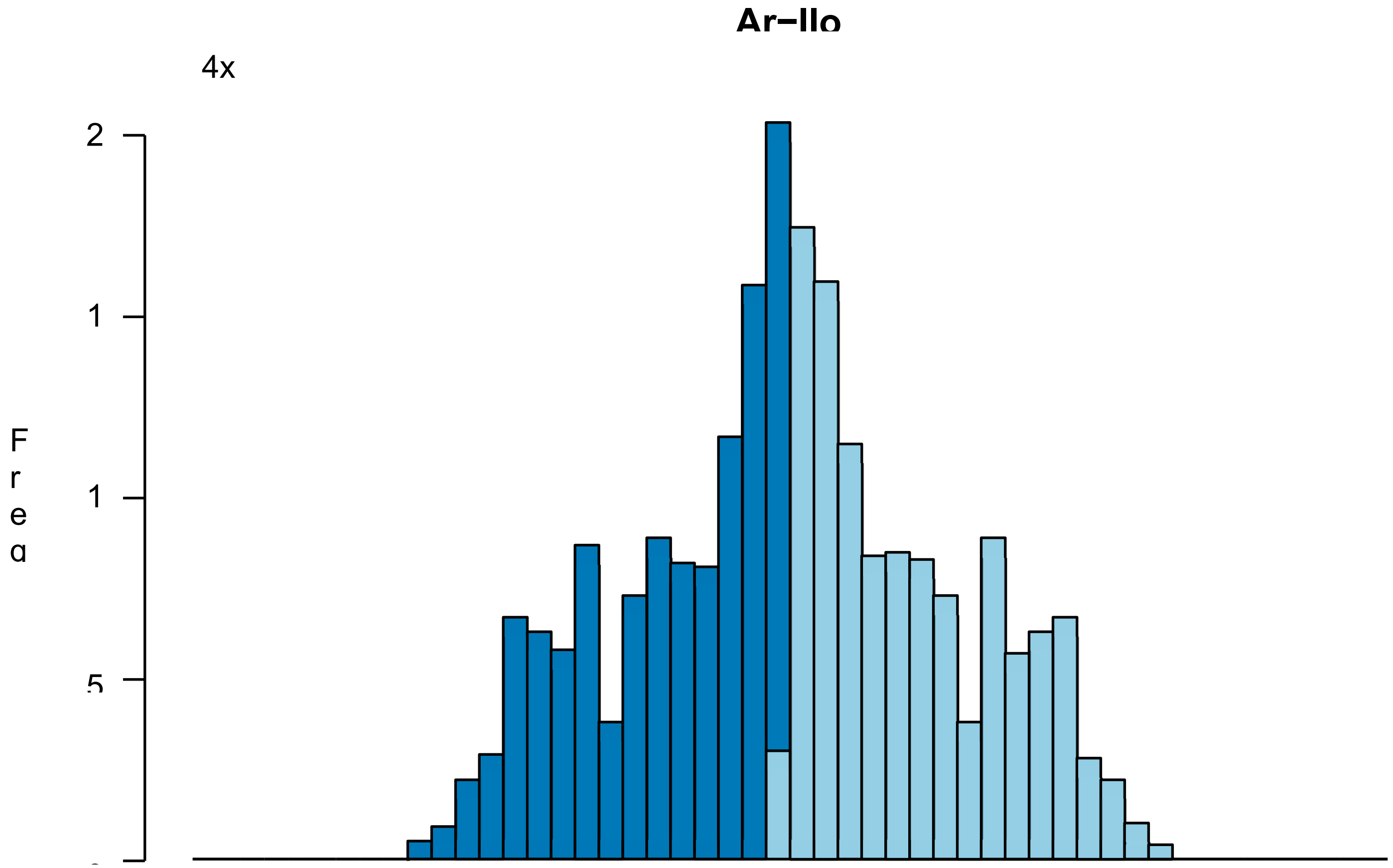
Ar−llo
4x
2
1
1
5
F
r
e
q

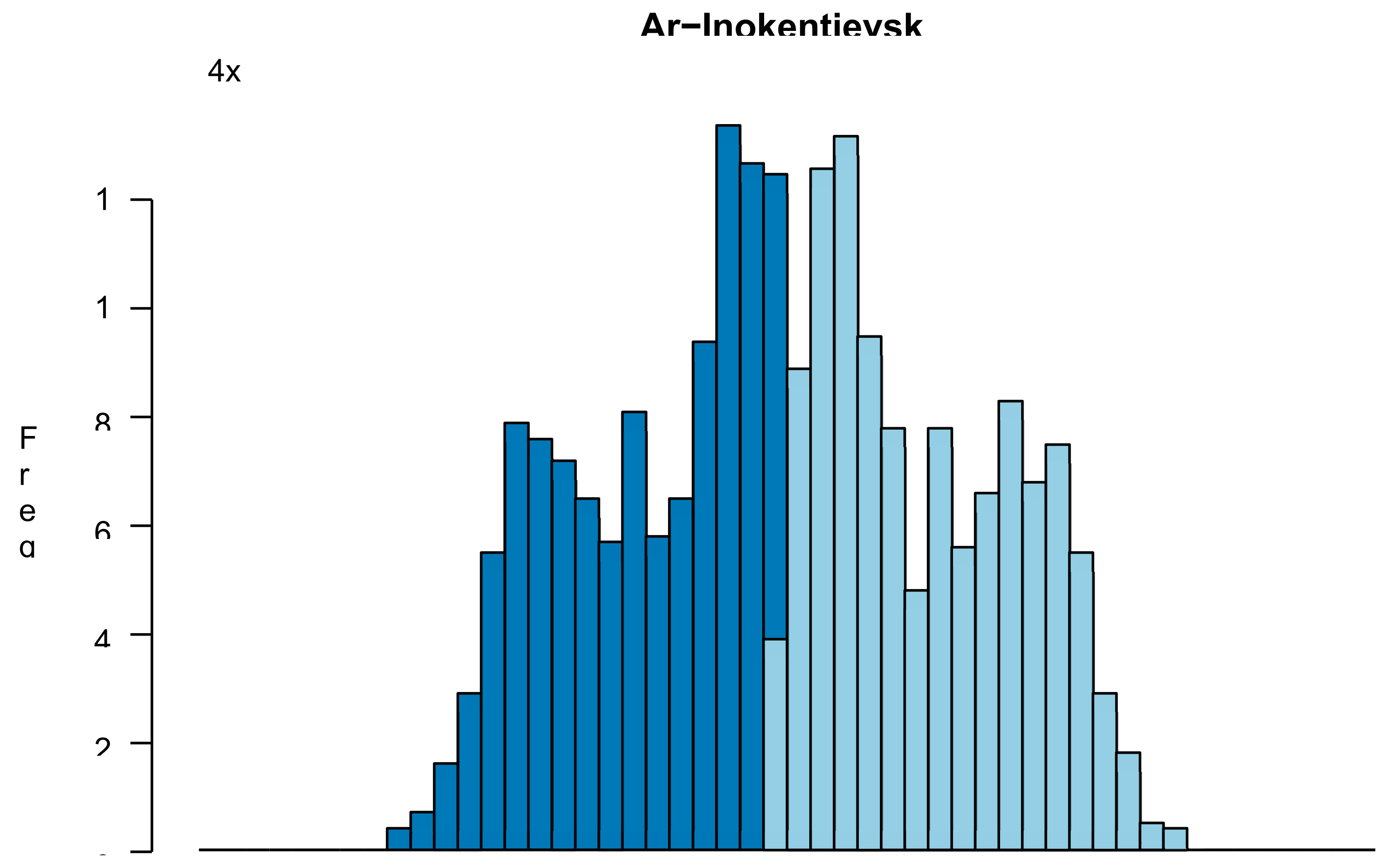

Ar−Inokentievsk
4x
1
1
8
6
4
2
F
r
e
q

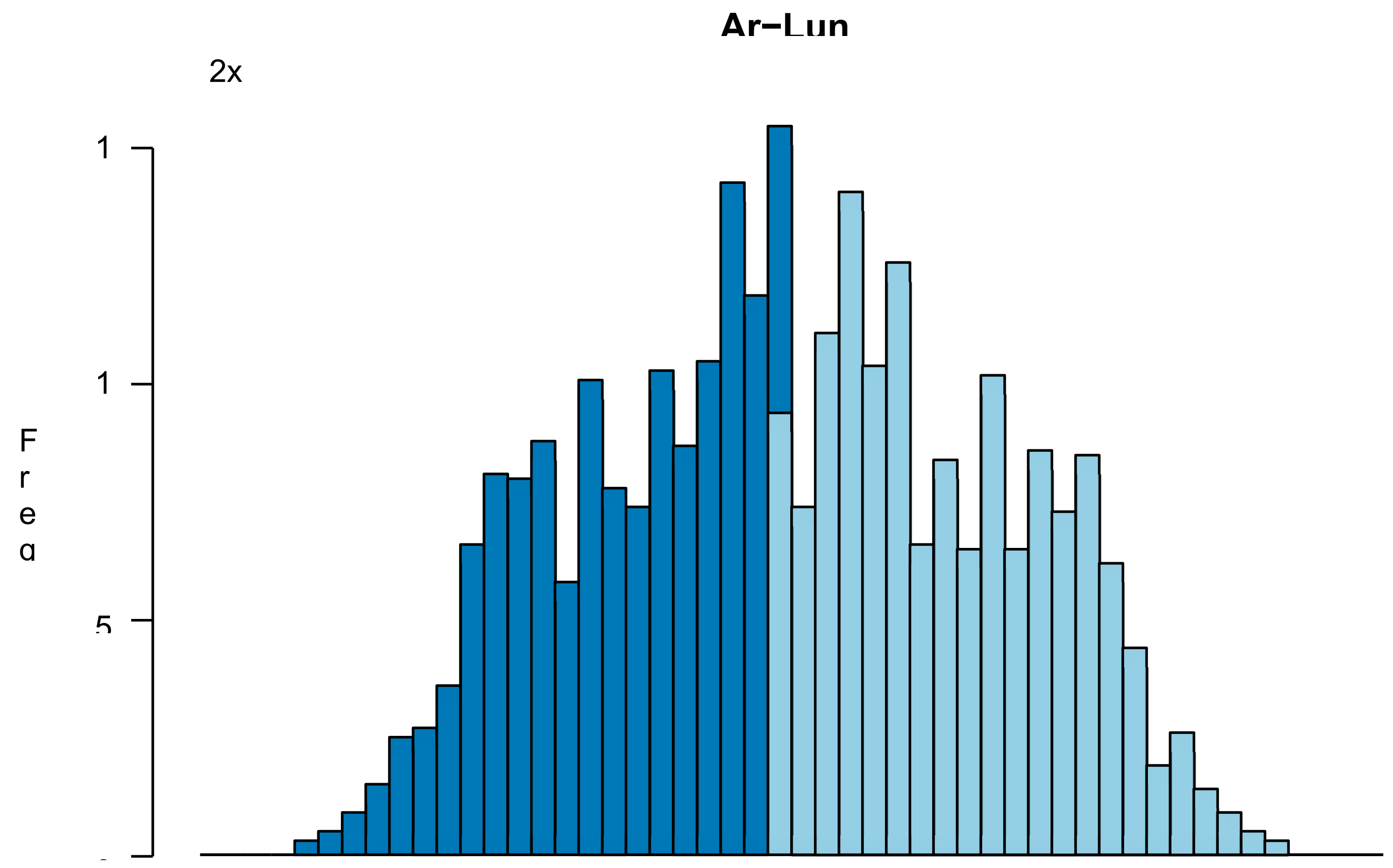

Ar−Lun
2x
1
1
5
F
r
e
q

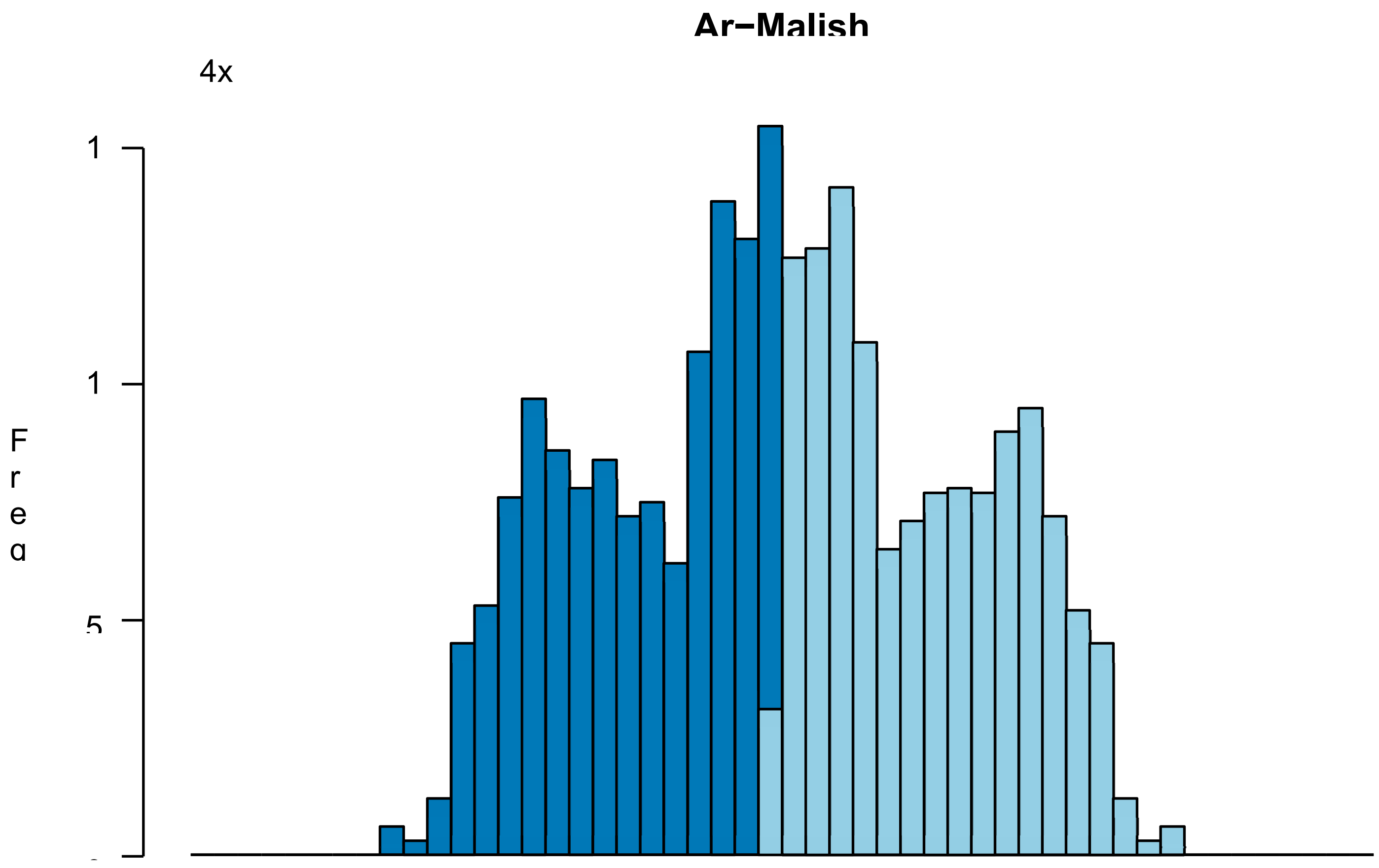

Ar−Malish
4x
1
1
5
F
r
e
q

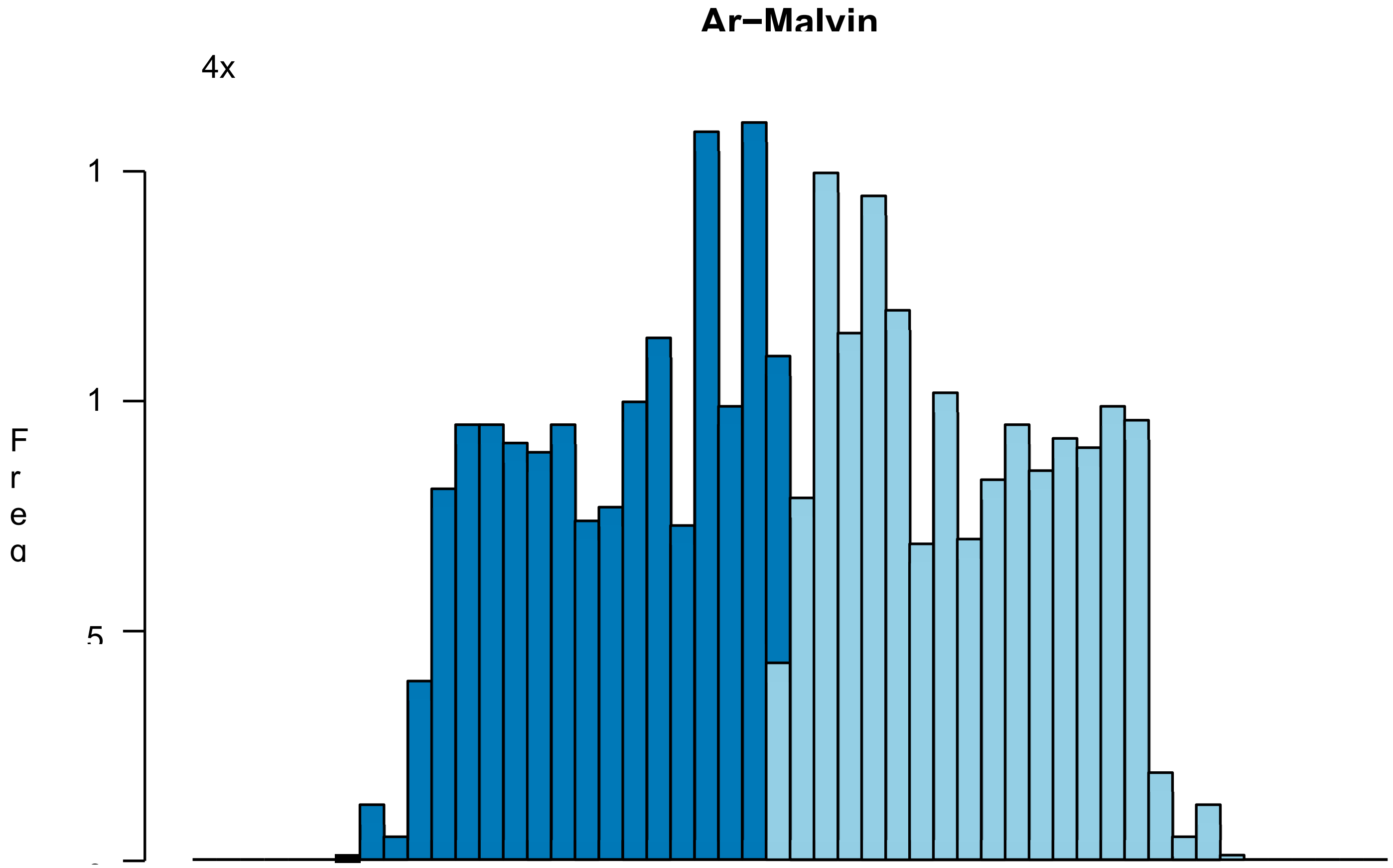
Ar−Malvin
4x
1
1
5
F
r
e
q

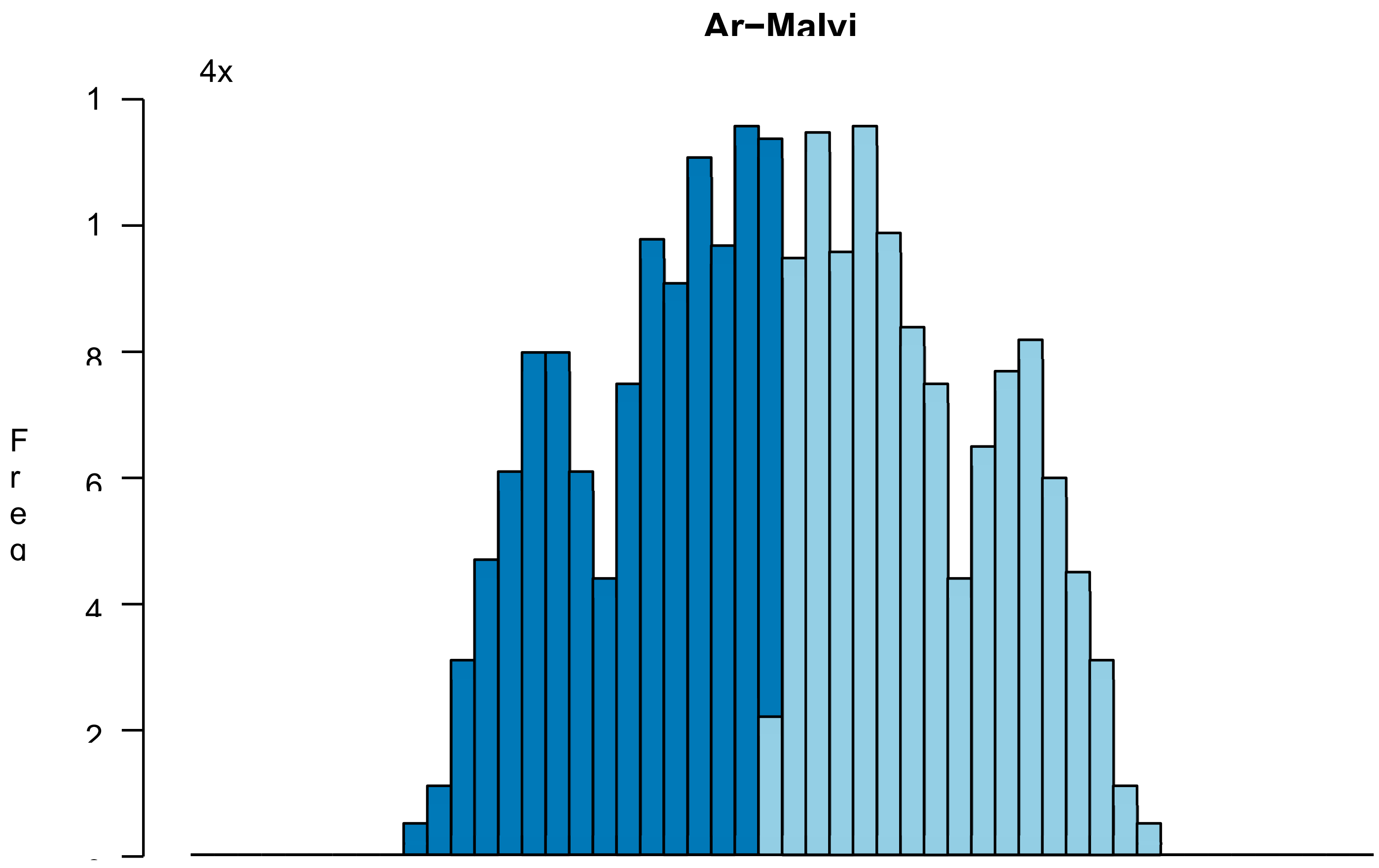

Ar−Malvi
4x
1
1
8
6
4
2
F
r
e
q

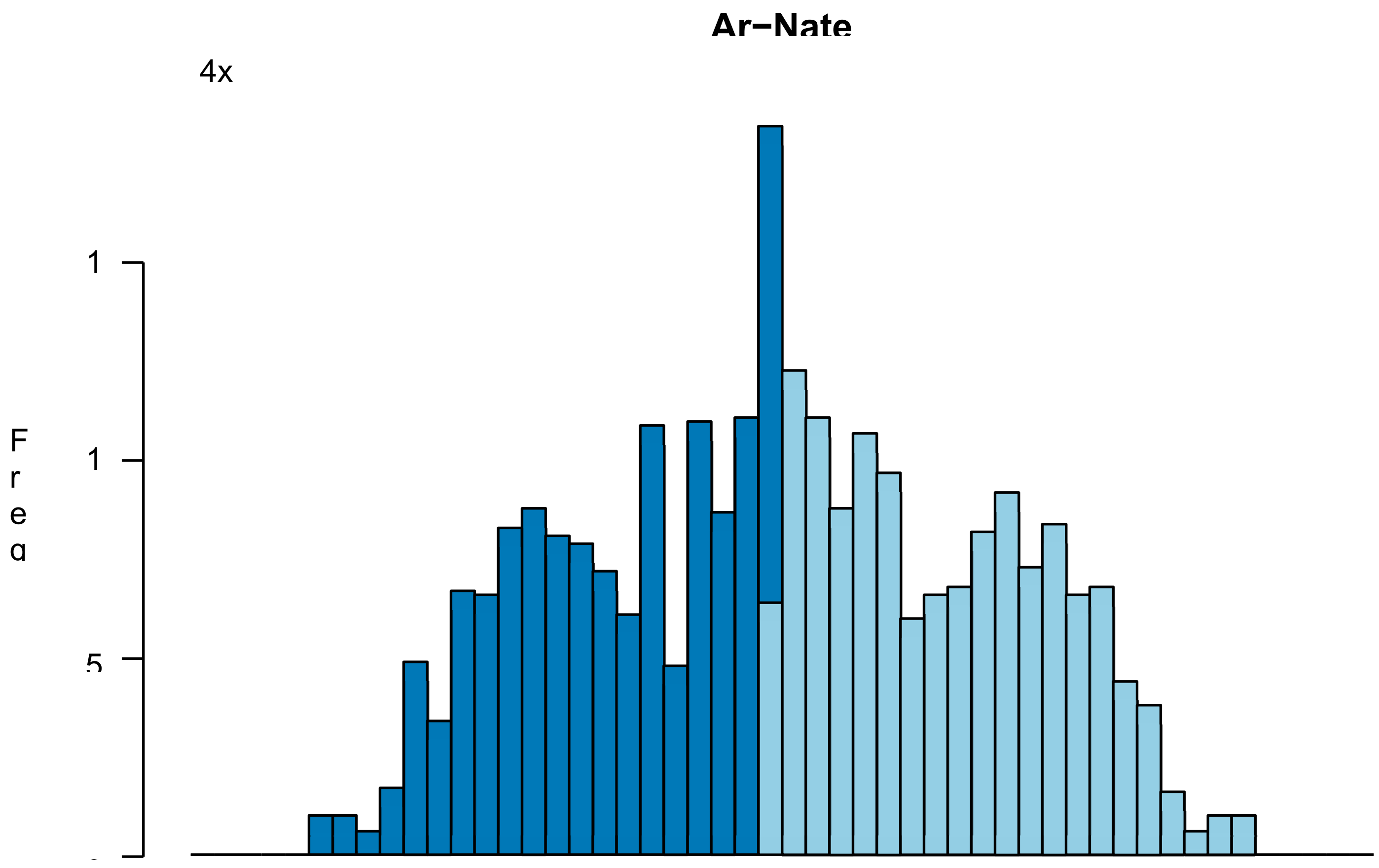

Ar−Nate
4x
F
r
e
q
1
1
5

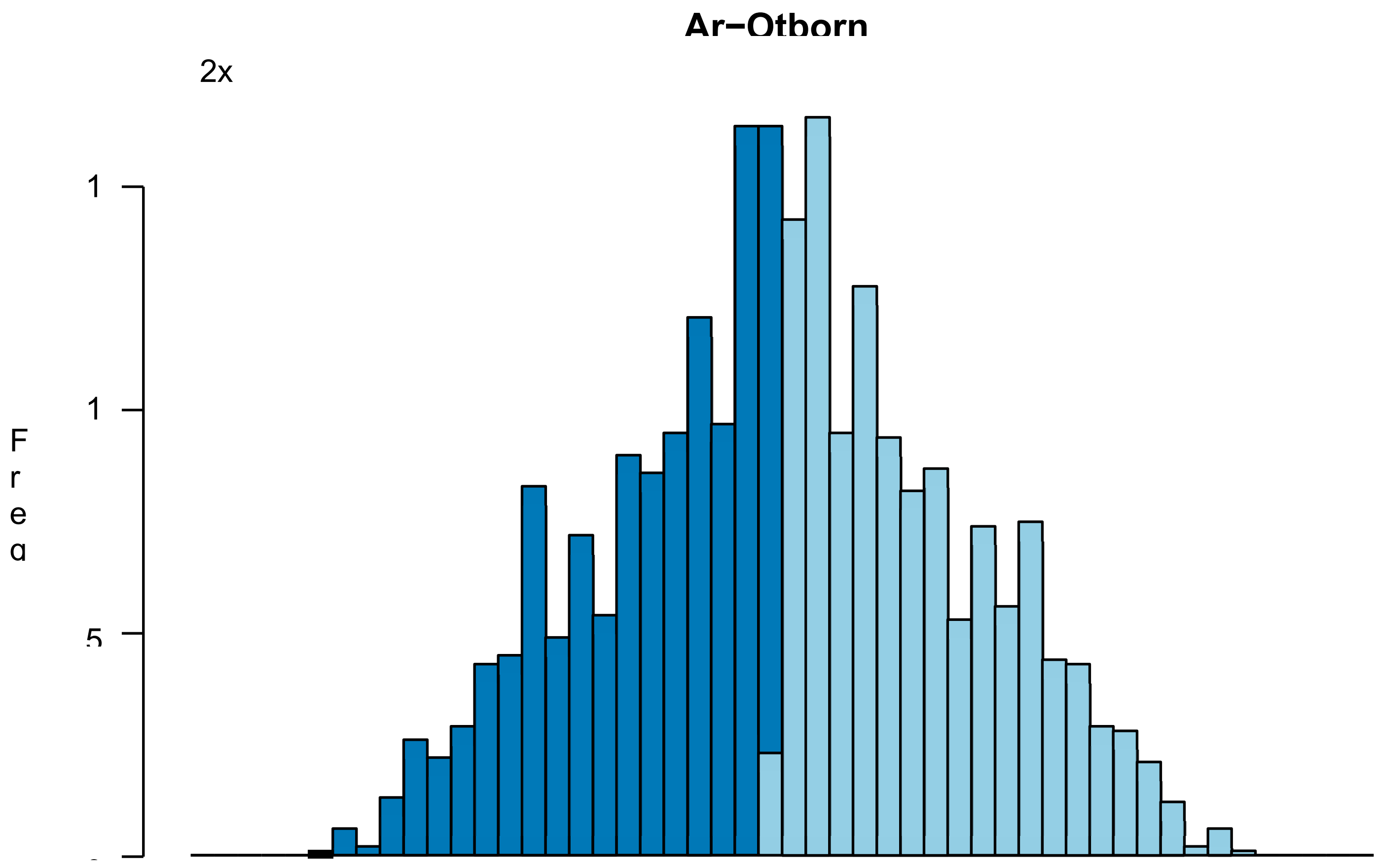

Ar−Otborn
2x
1
1
5
F
r
e
q

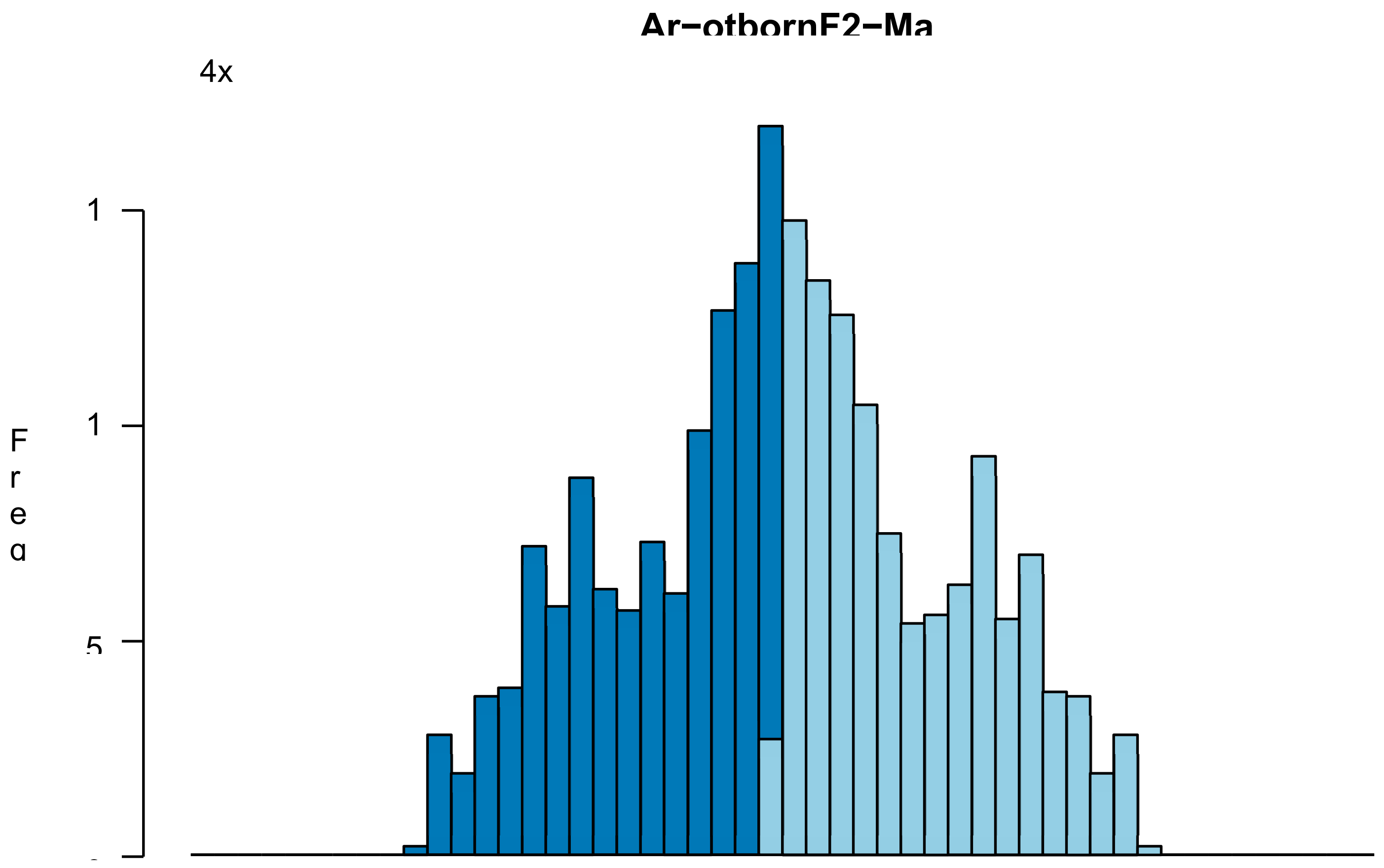
Ar−otbornF2−Ma
4x
1
1
5
F
r
e
q

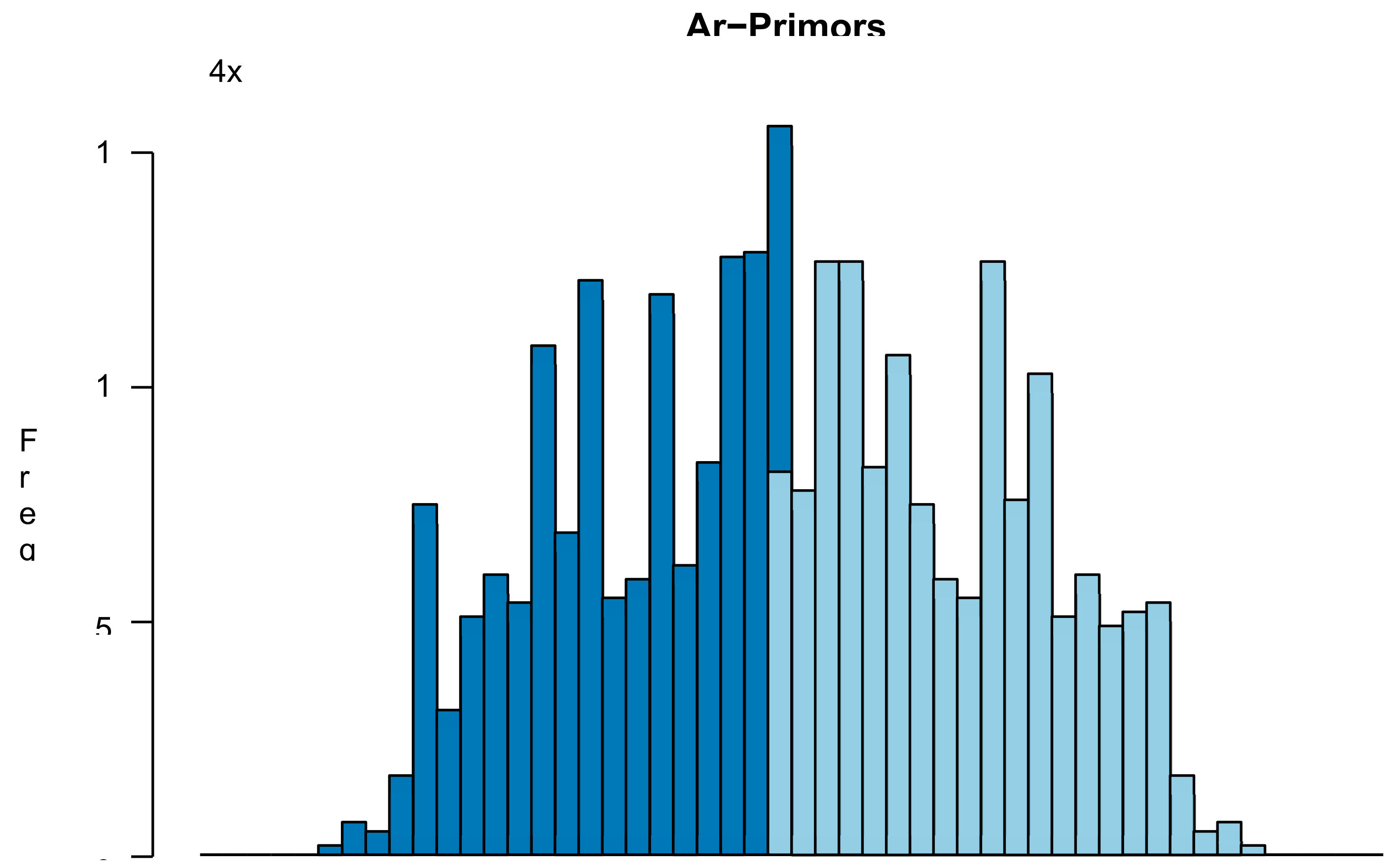
Ar−Primors
4x
1
1
5
F
r
e
q

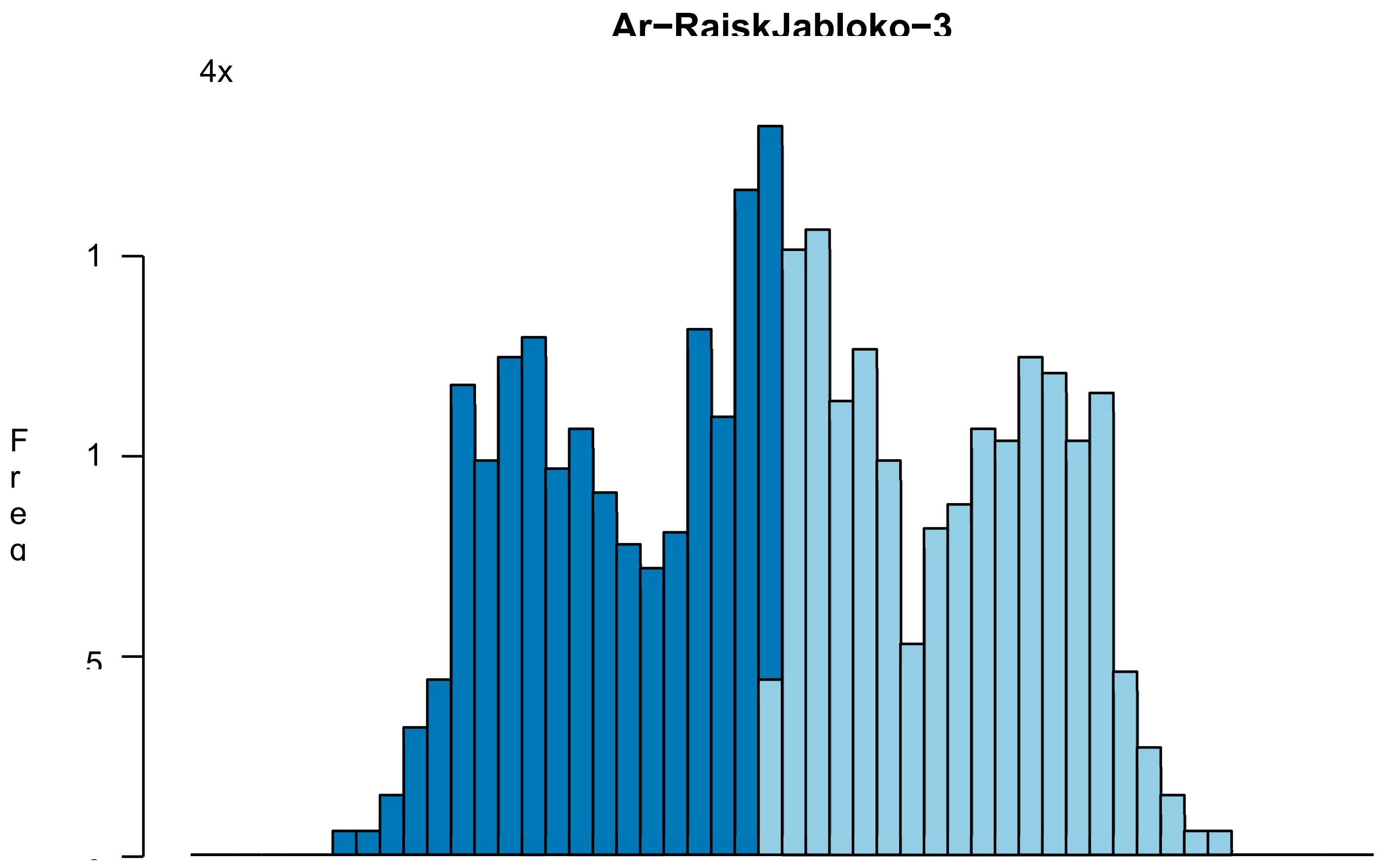

Ar−RaiskJabloko−3
4x
F
r
e
q
1
1
5

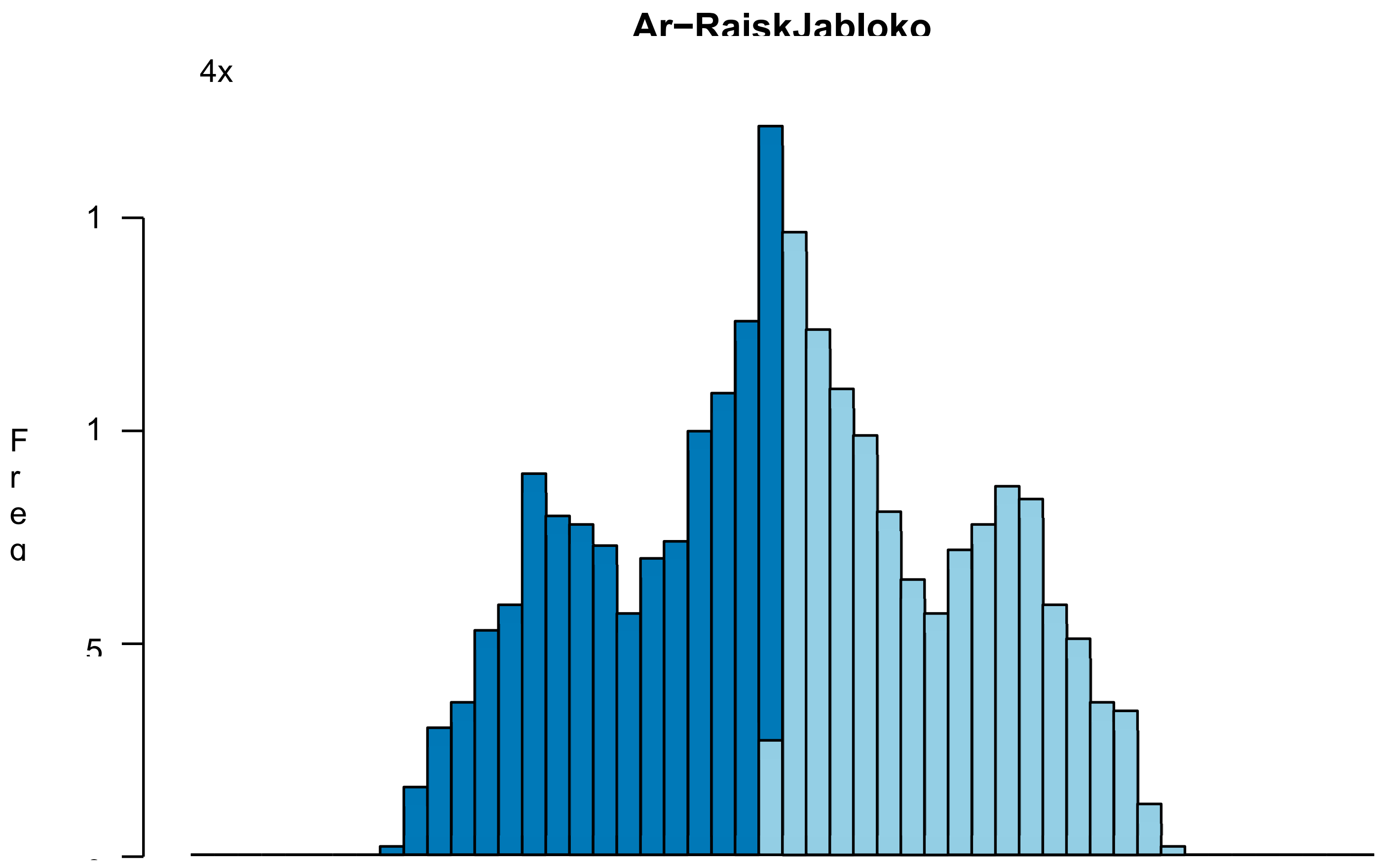

Ar−RaiskJabloko
4x
1
1
5
F
r
e
q

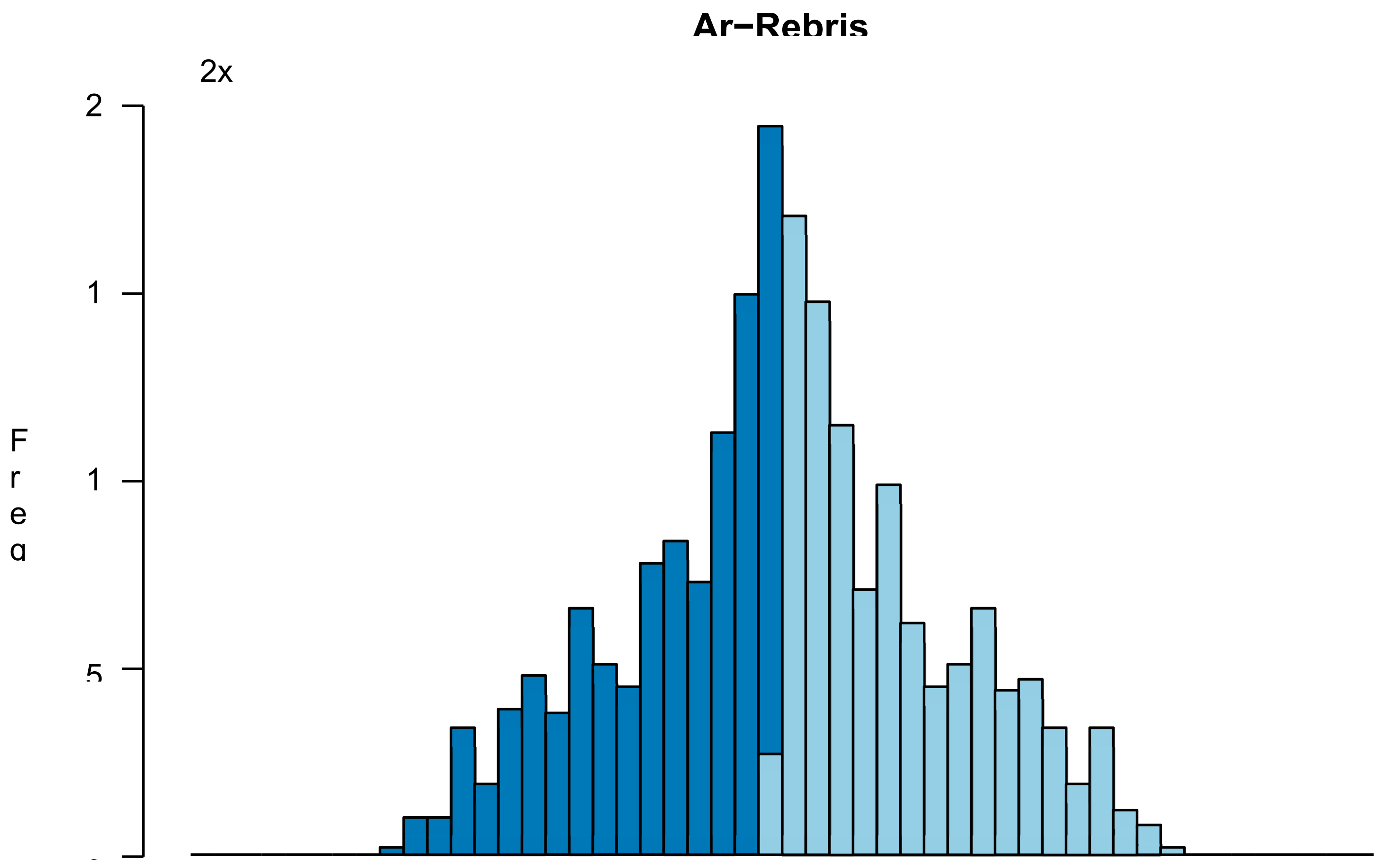

Ar−Rebris
2x
2
1
1
5
F
r
e
q

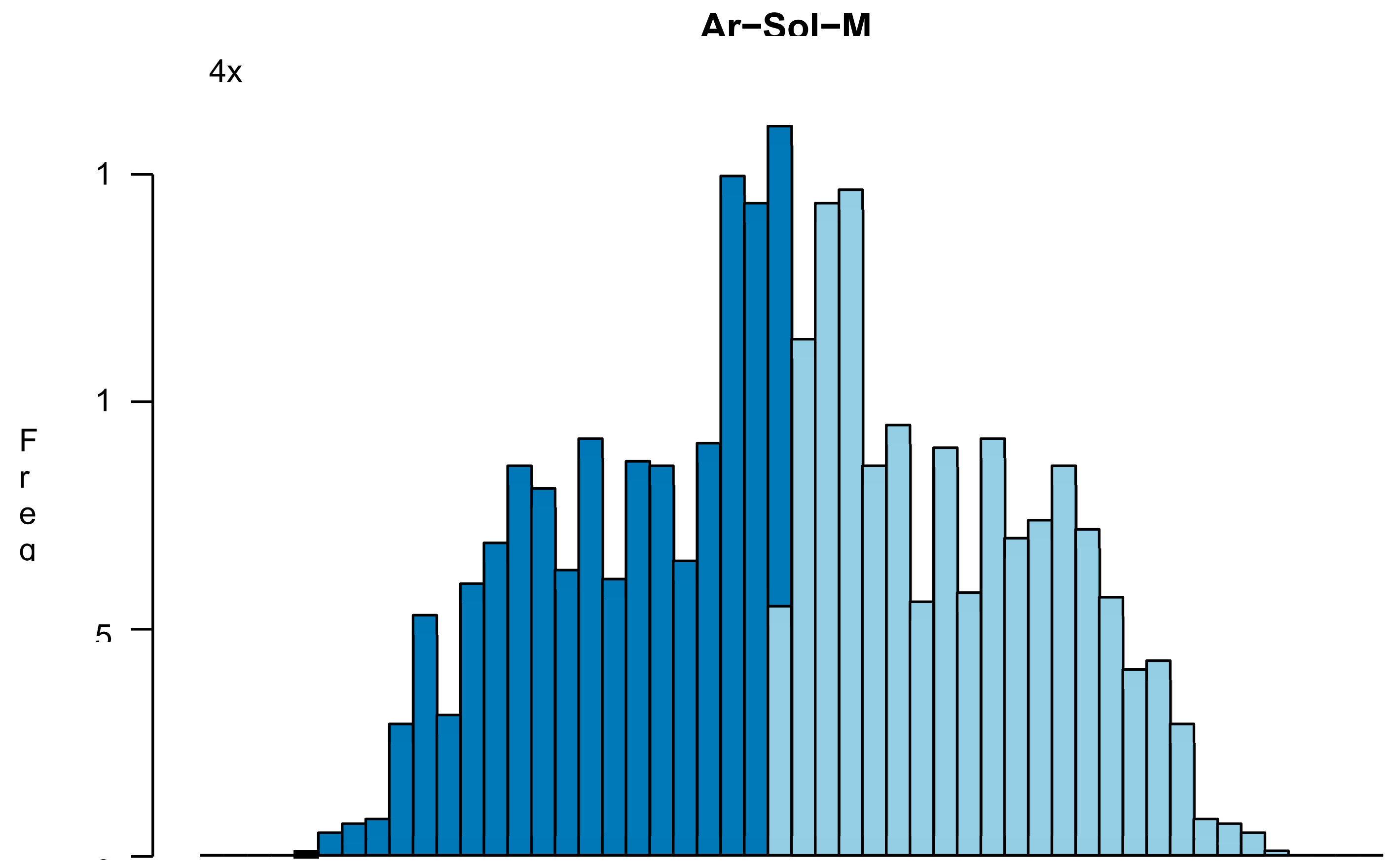

Ar−Sol−M
4x
1
1
F
r
e
q

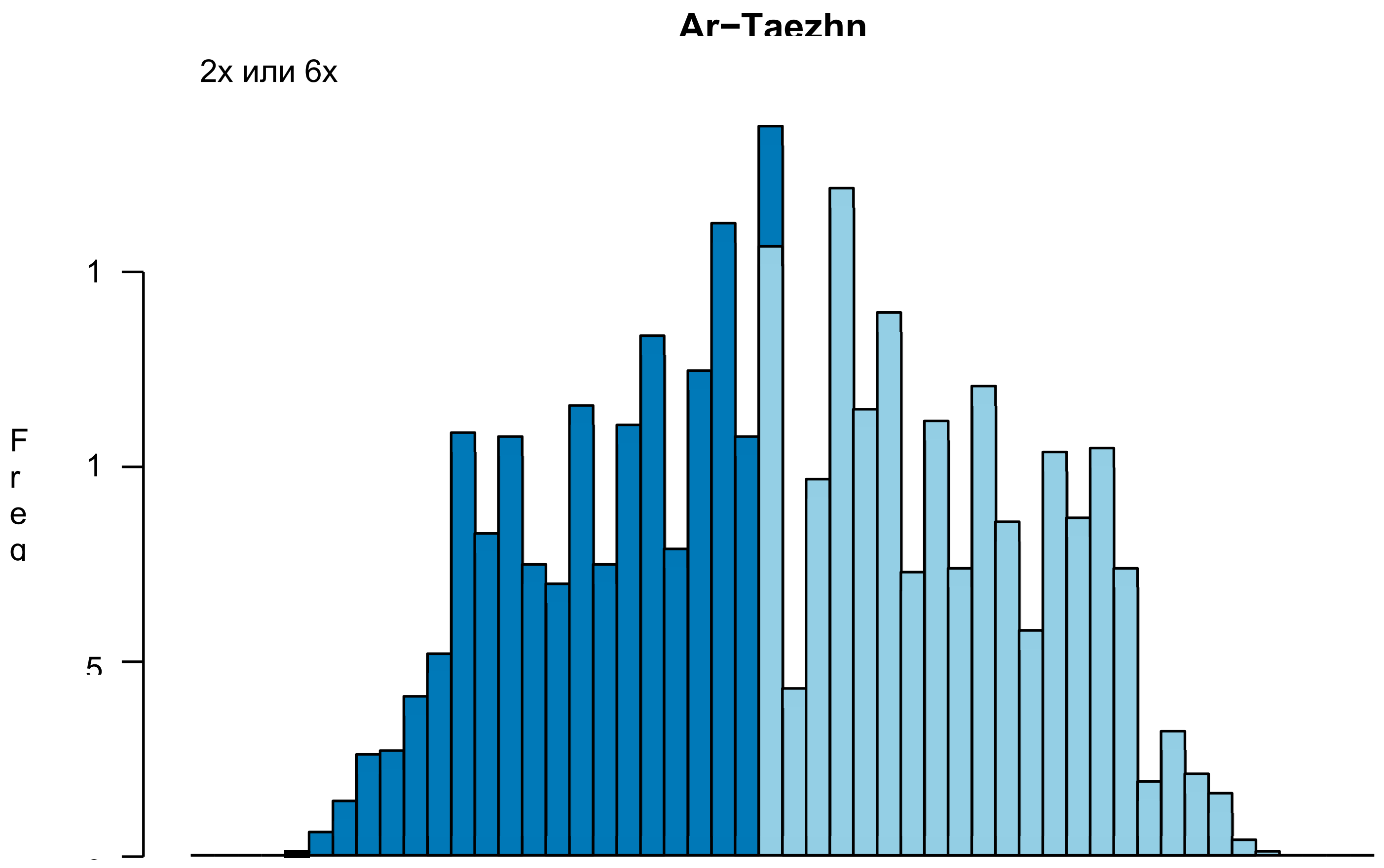
Ar−Taezhn
2x или 6x
F
r
e
q
1
1
5

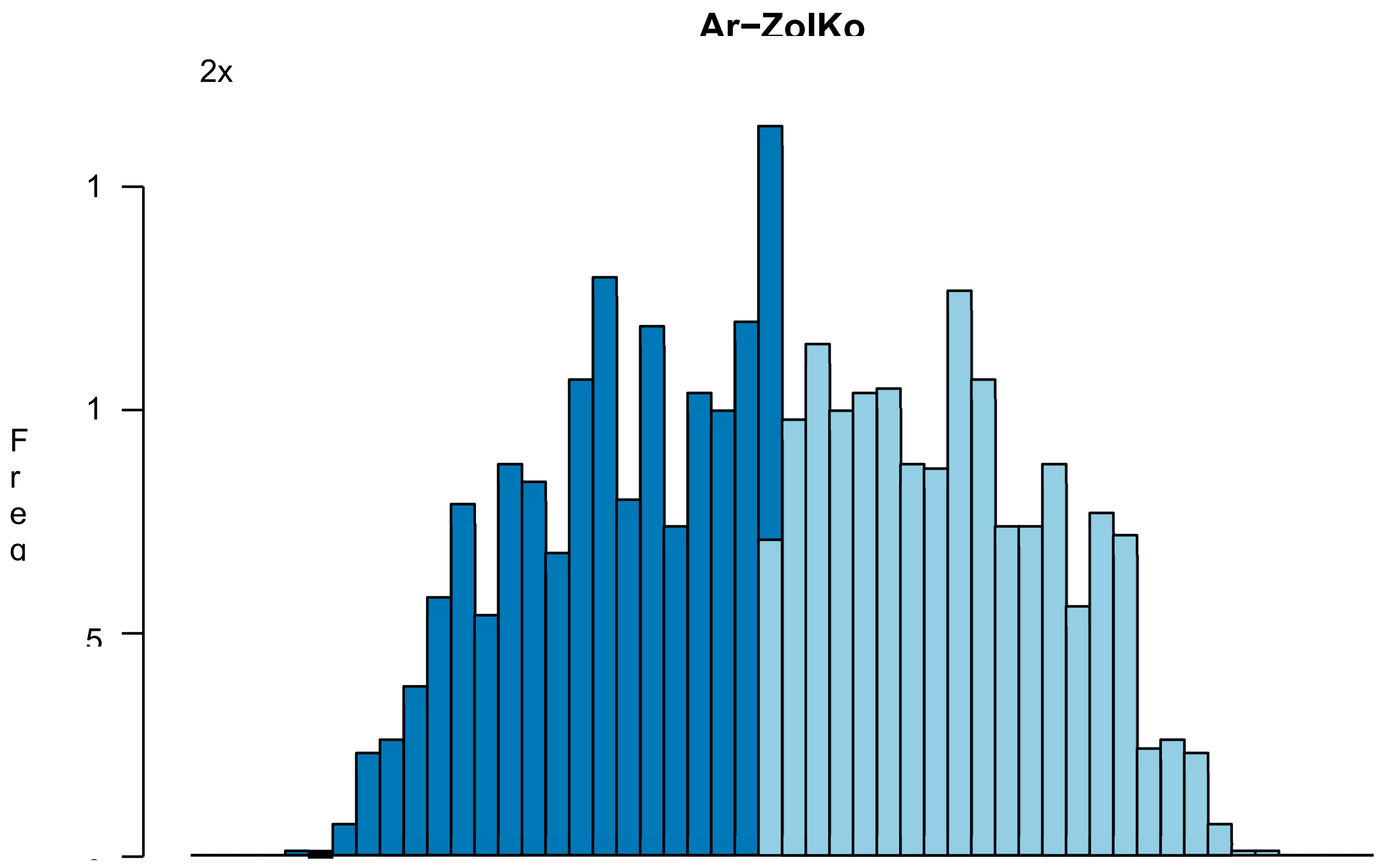
Ar−ZolKo
2x
1
1
5
F
r
e
q

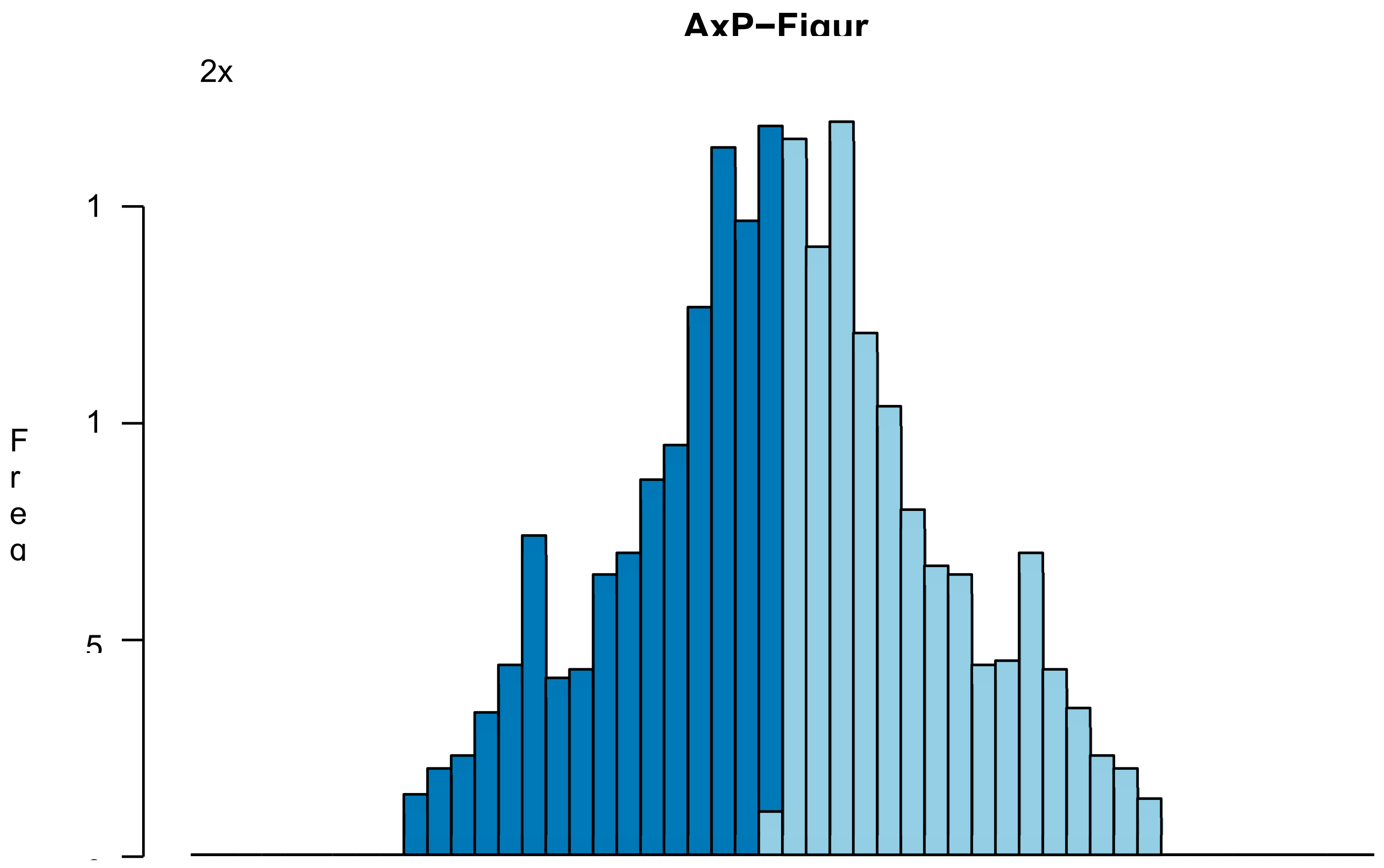
AxP–Figur
2x
1
1
5
F
r
e
q

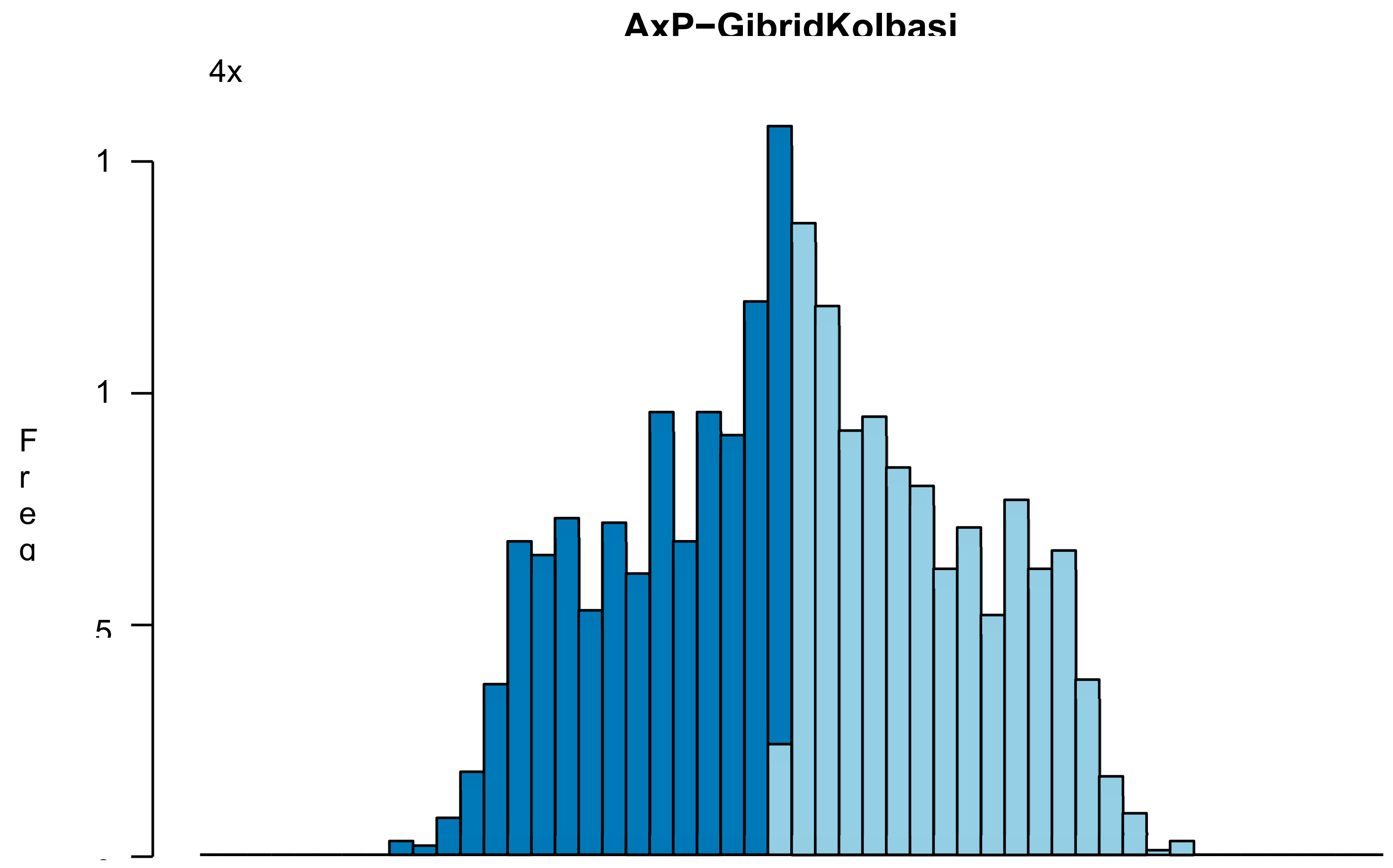

AxP−GibridKolbasi
4x
1
1
5
F
r
e
q

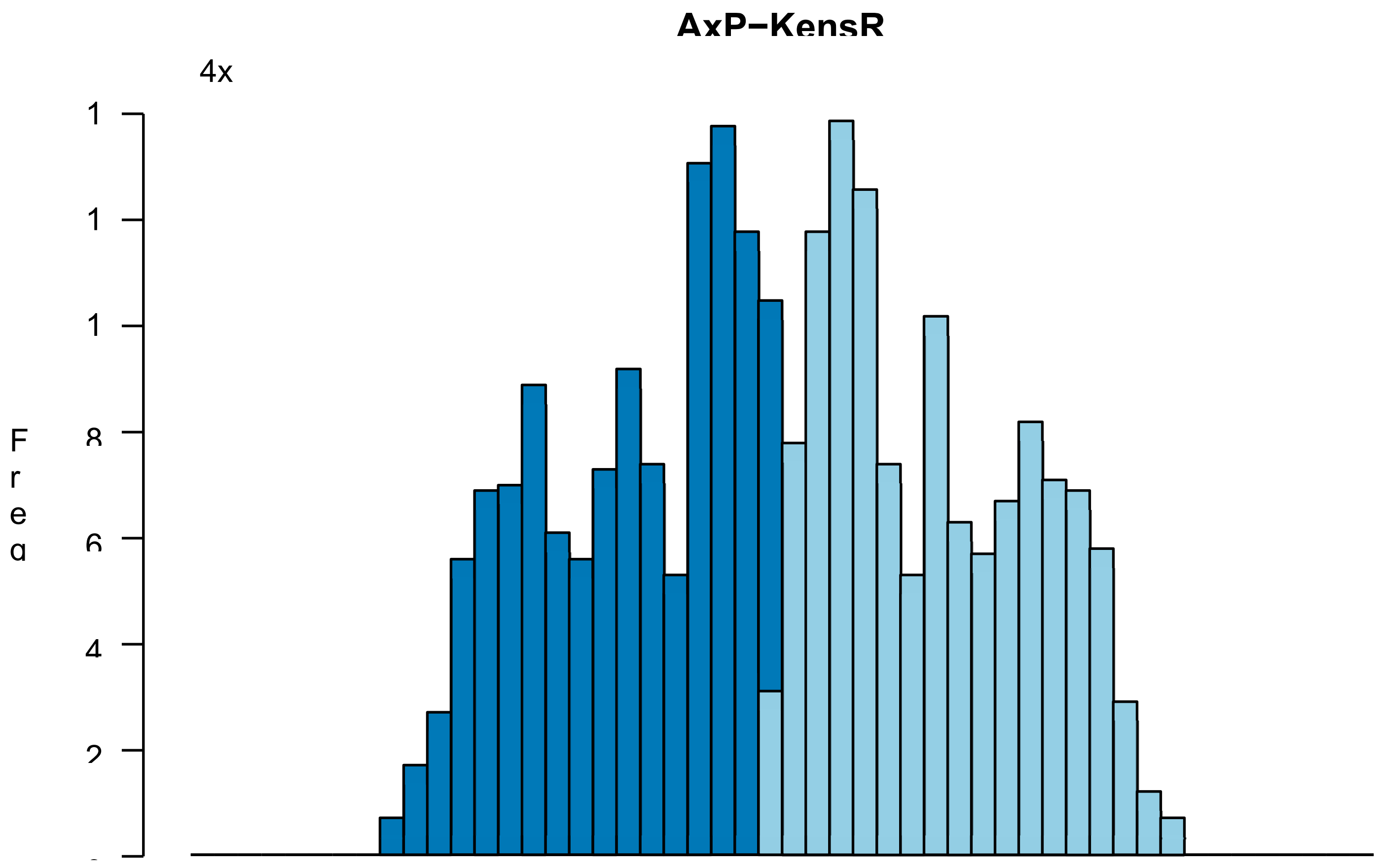
AxP−KensR
4x
1
1
1
8
6
4
2
F
r
e
q

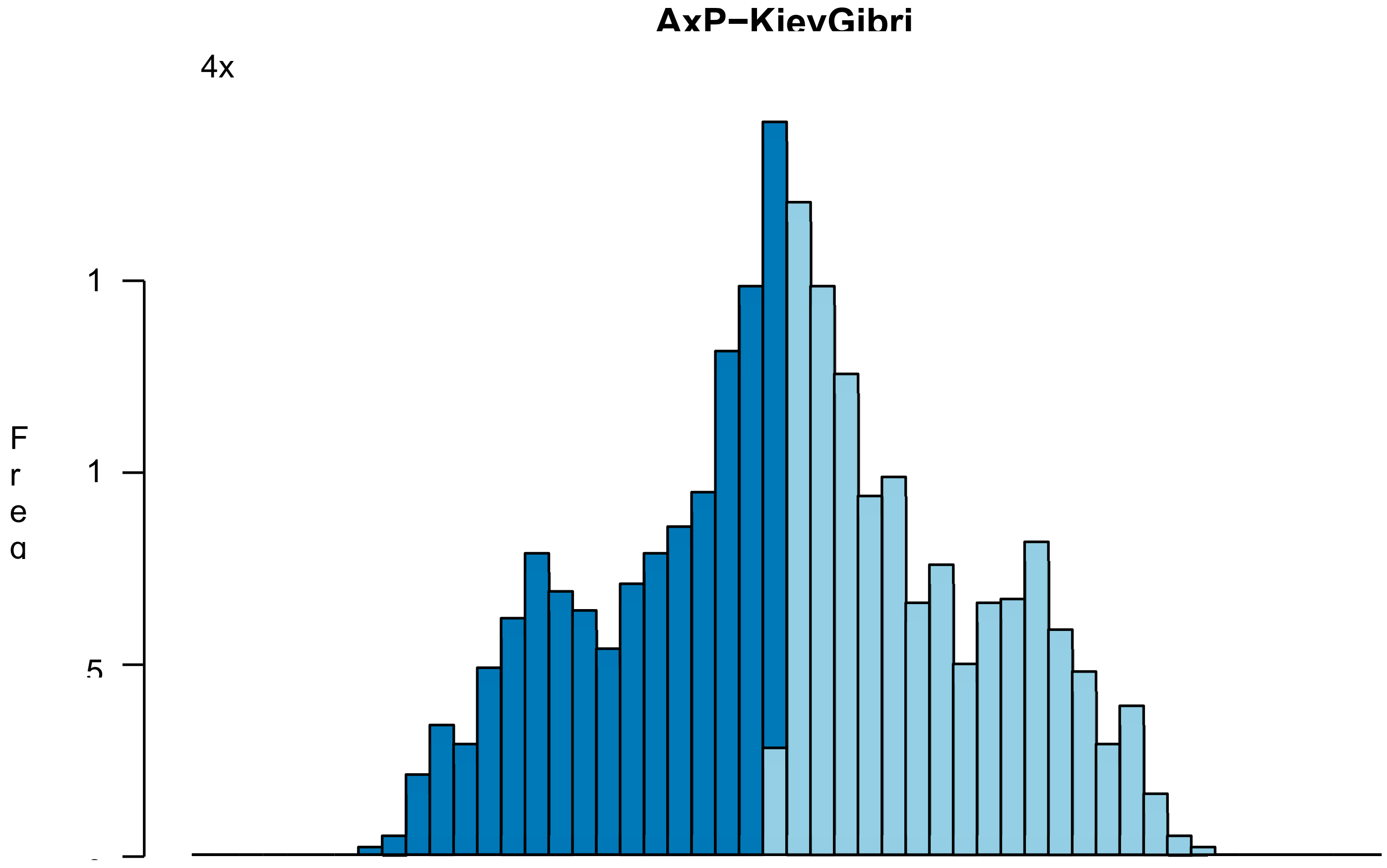
AxP−KievGibri
4x
F
r
e
q
1
1
5

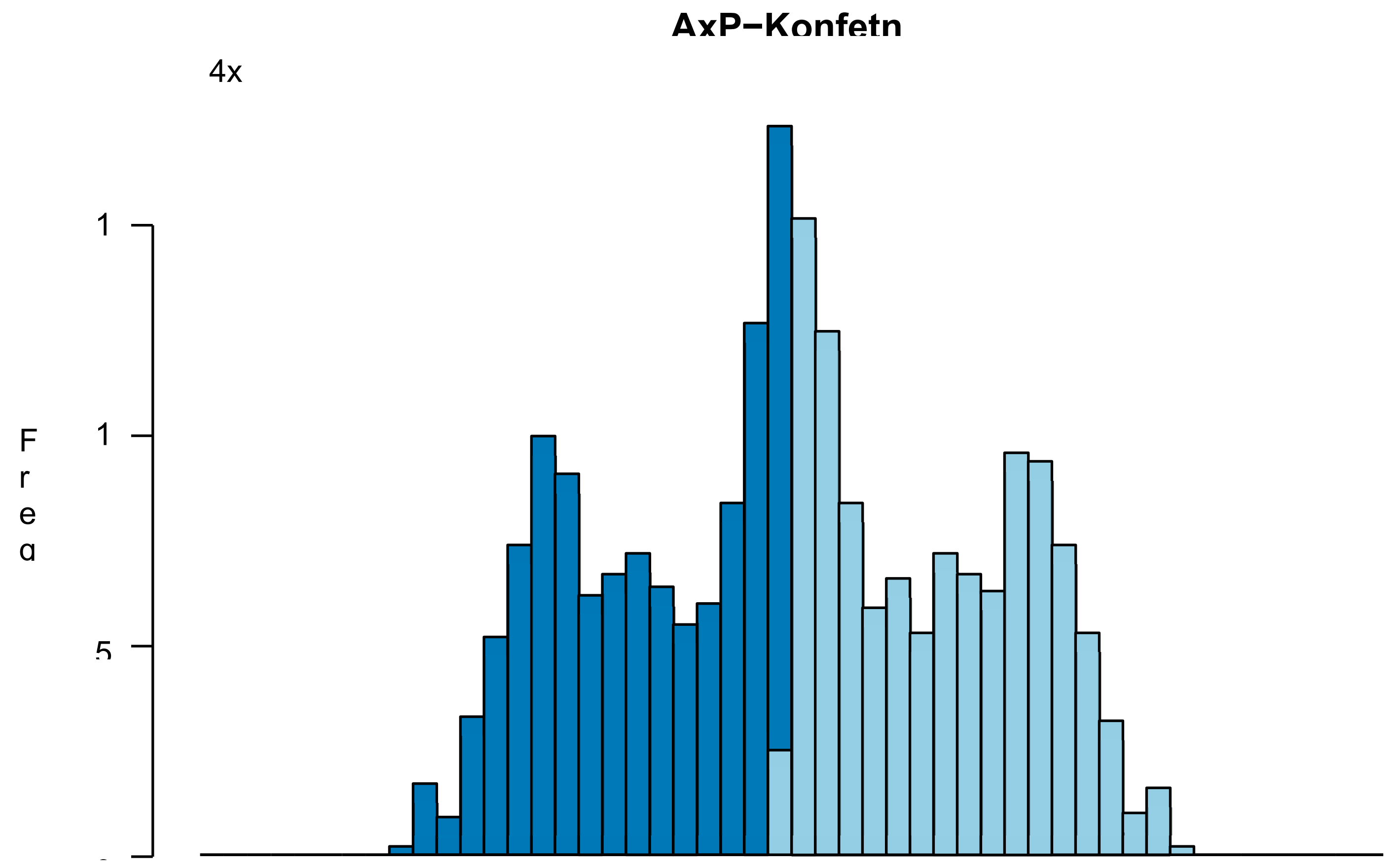

AxP−Konfetn
4x
1
1
5
F
r
e
q

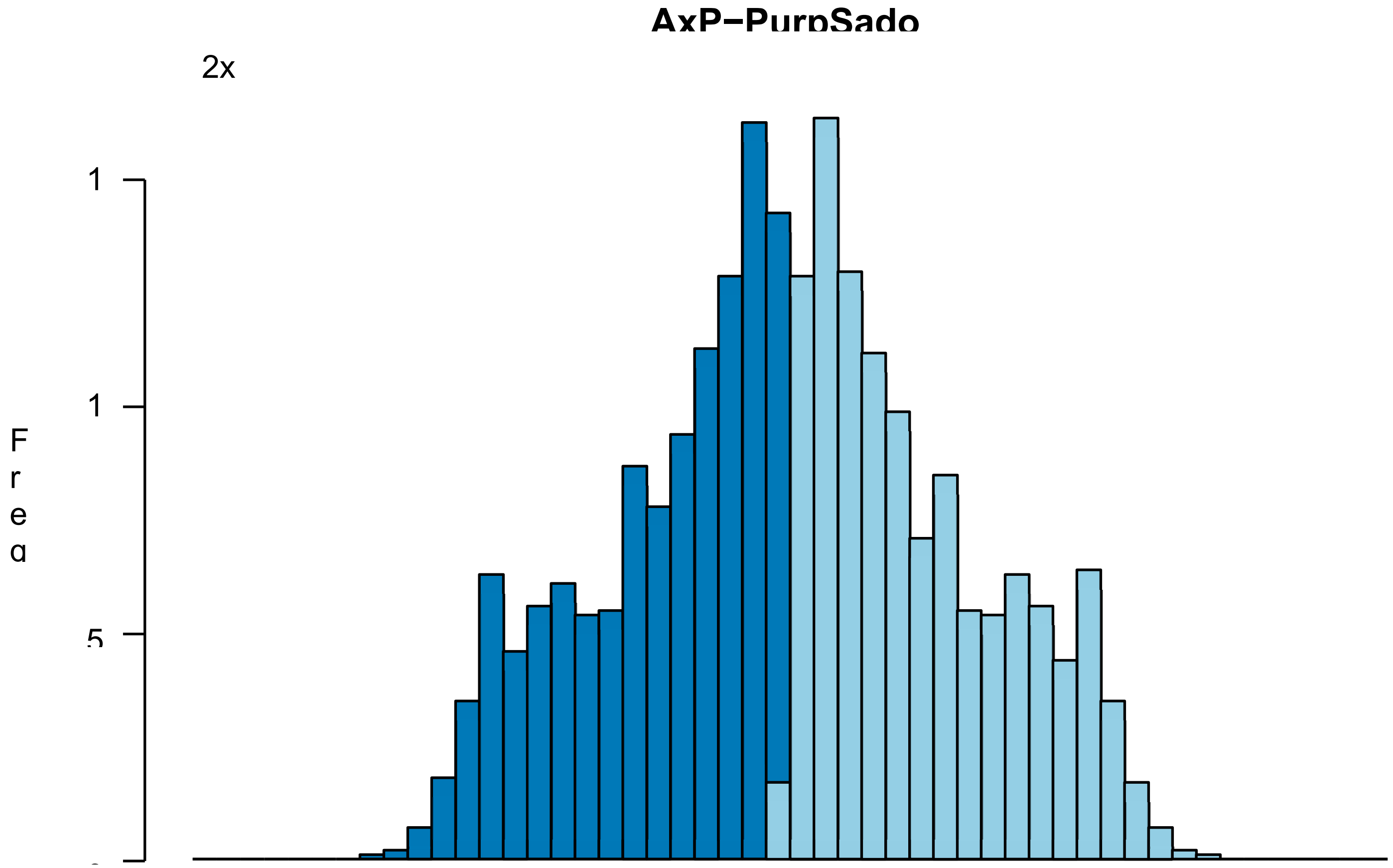
AxP−PurpSado
2x
1
1
F
r
e
q
5

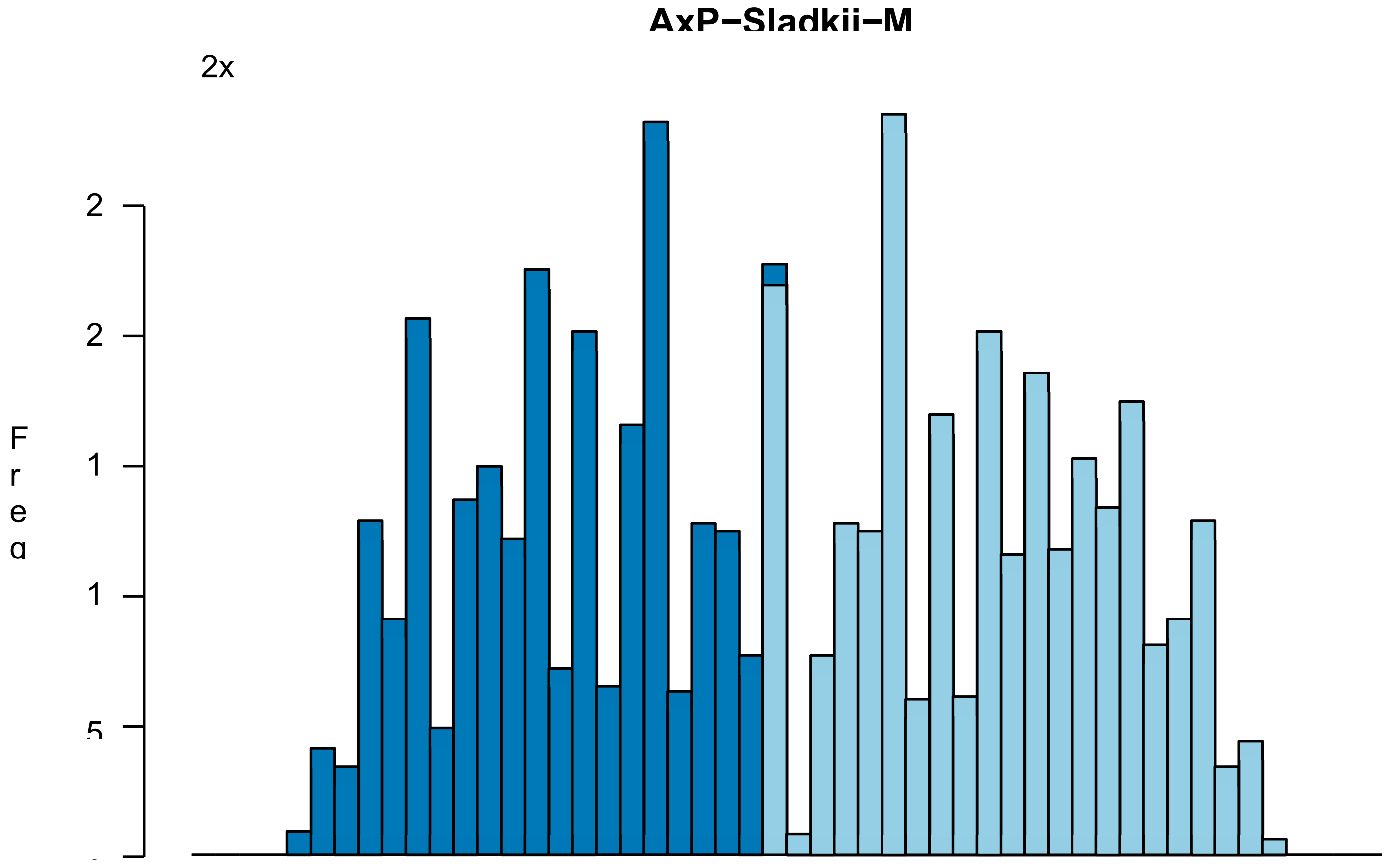
AxP−Sladkii−M
2x
2
2
1
1
5
F
r
e
q

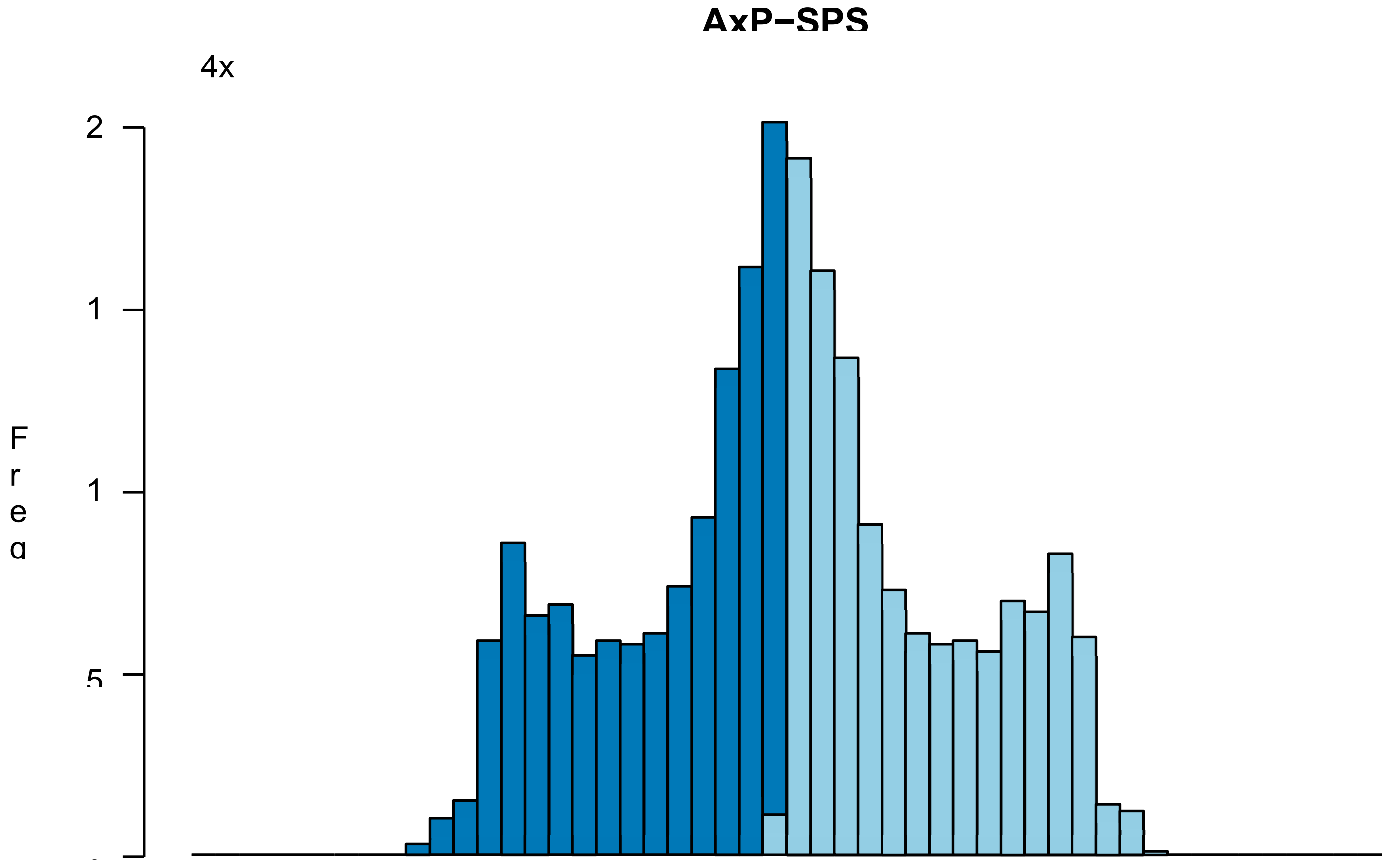
AxP−SPS
4x
2
1
1
5
F
r
e
q

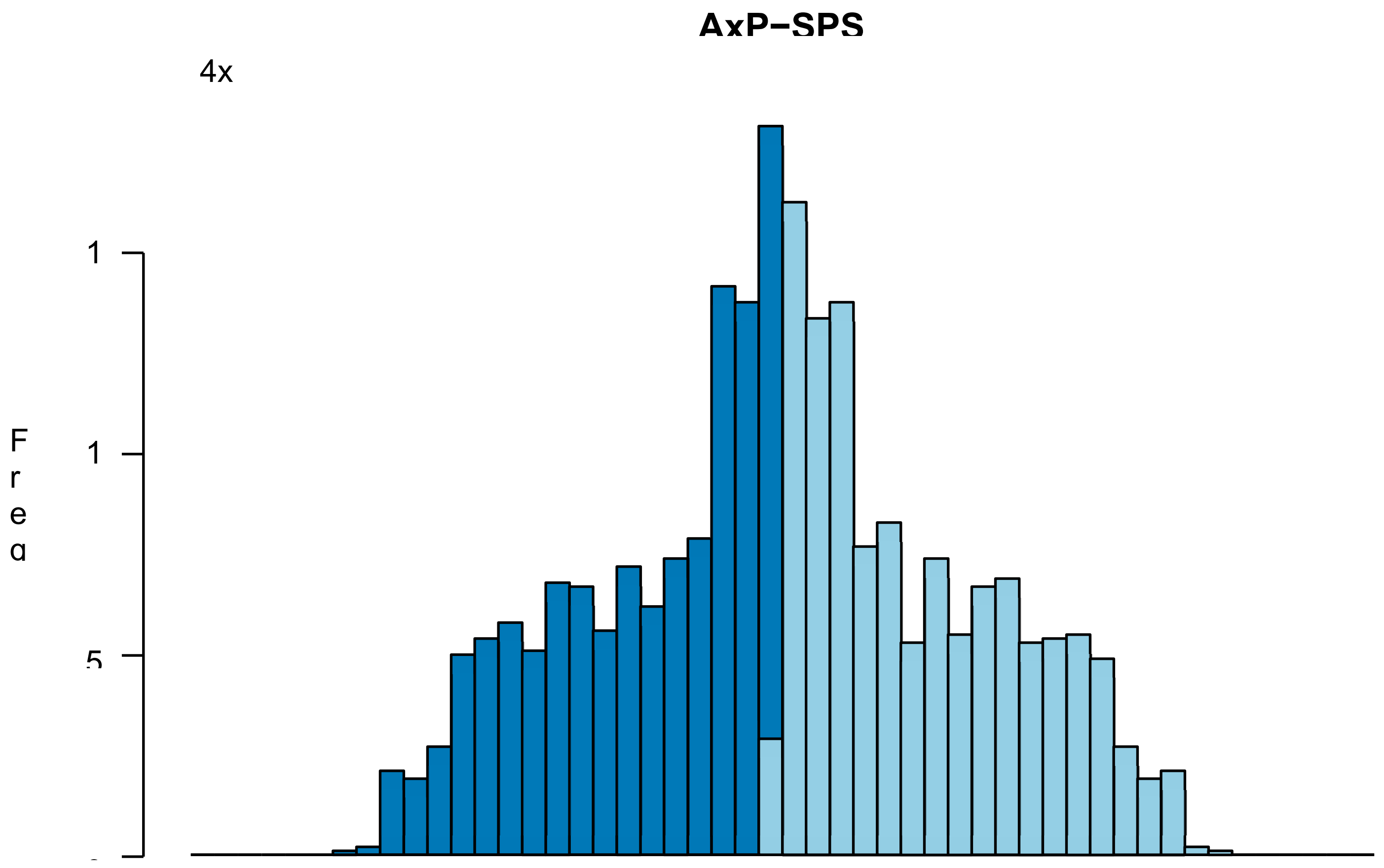
AxP-SPS
4x
F
r
e
q
1
1
5

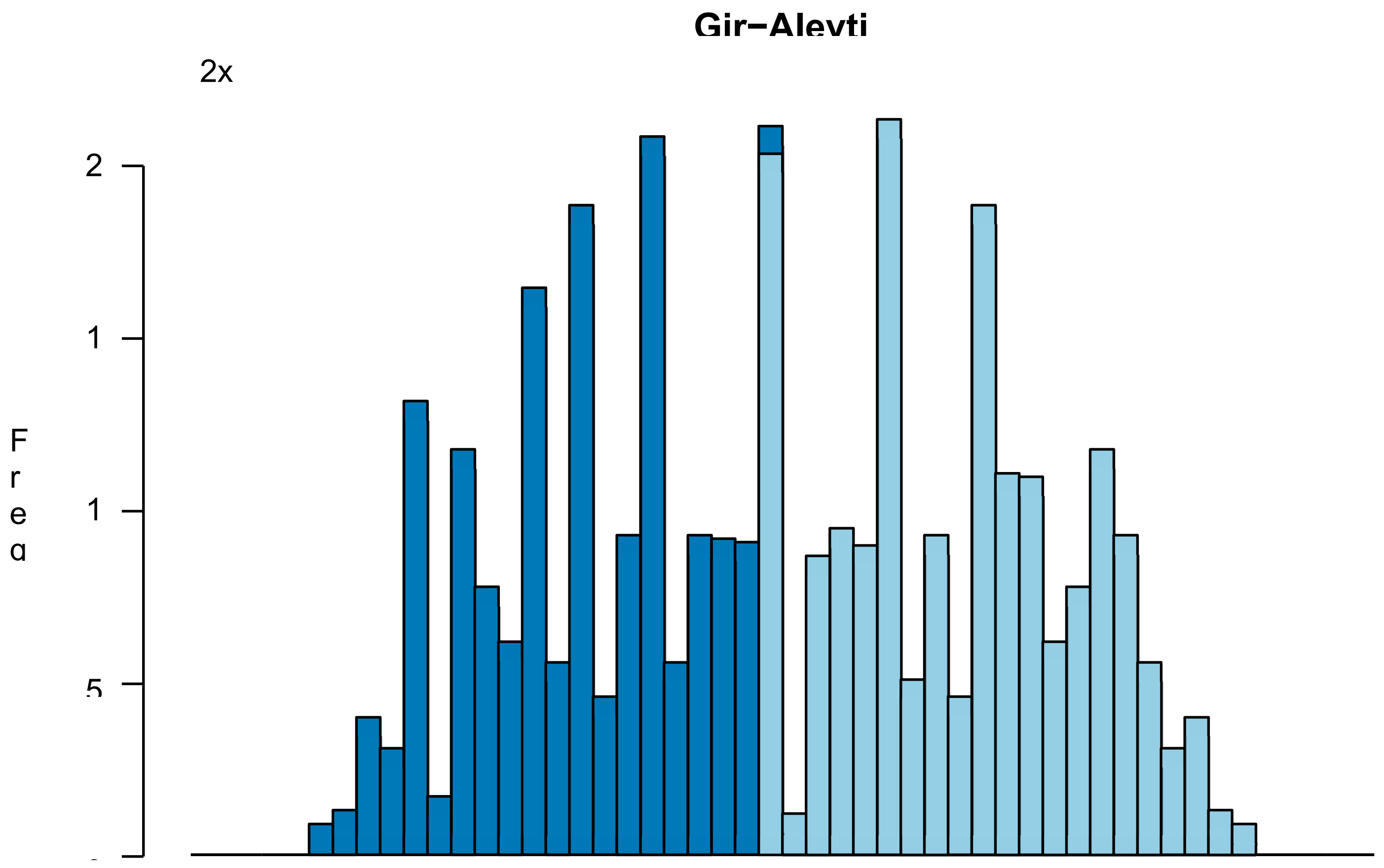

Gir−Alevti
2x
2
1
1
5
F
r
e
q

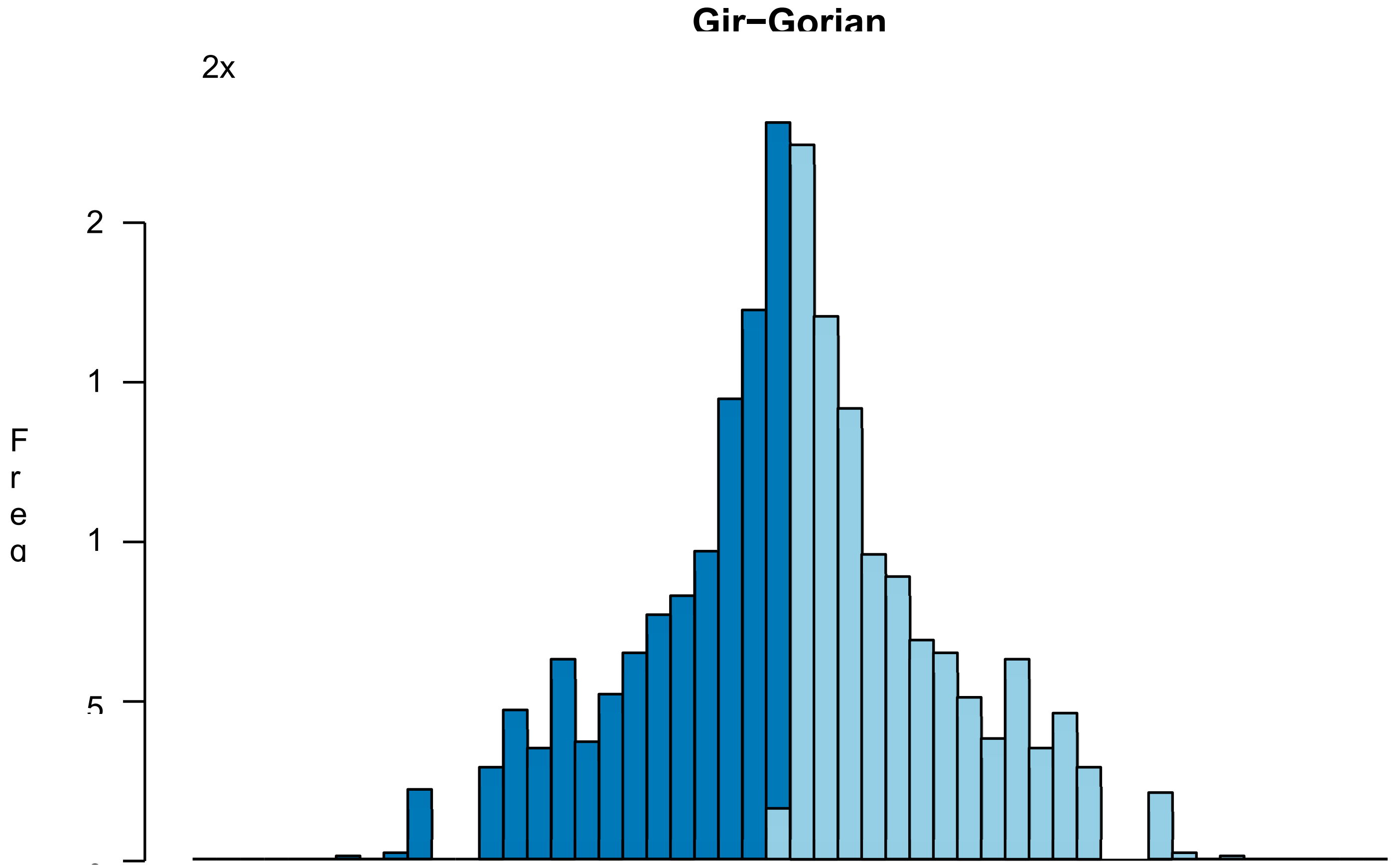
Gir−Gorian
2x
2
1
F
r
e
q
1
5

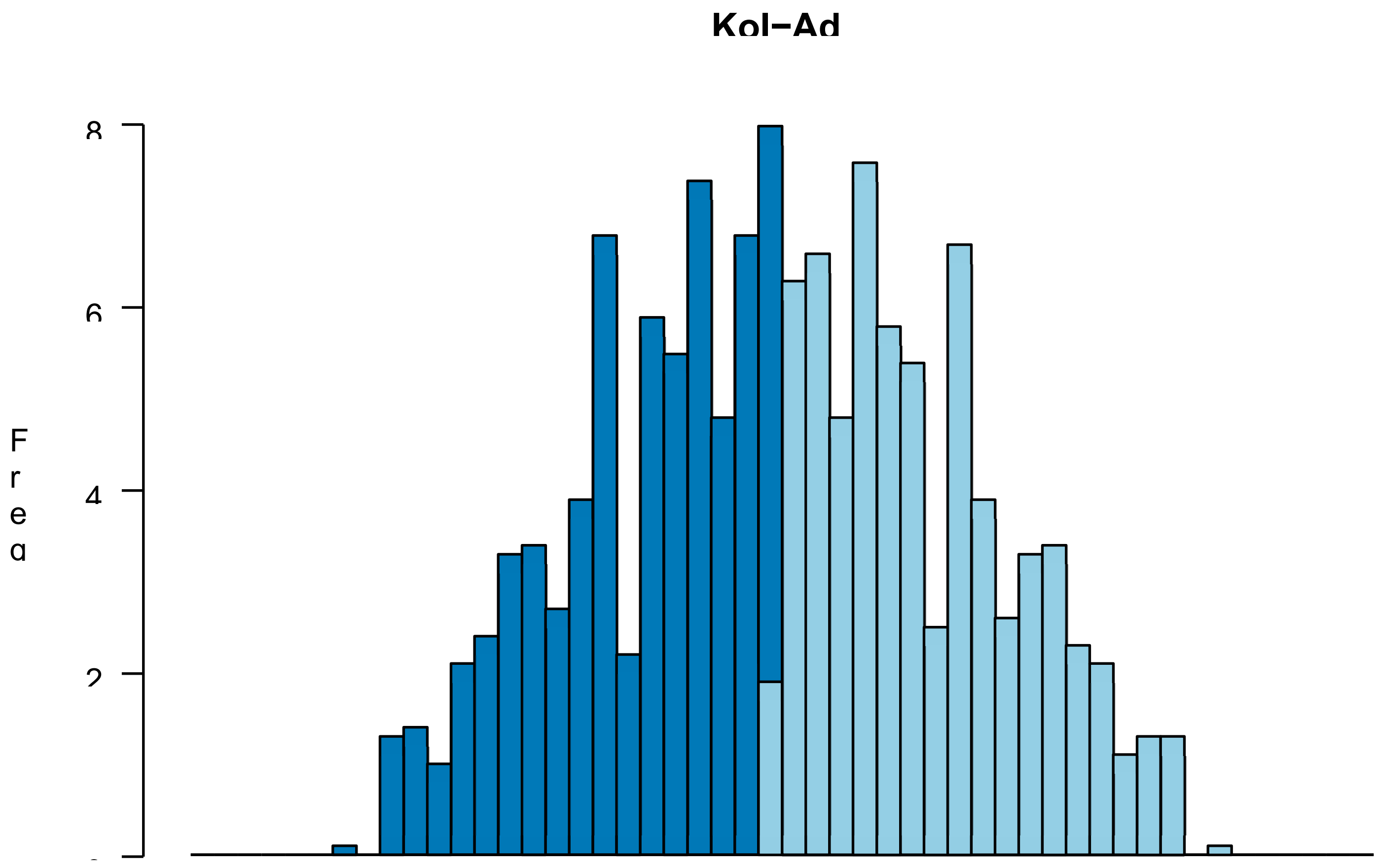
Kol-Ad
F
r
e
q
8
6
4
2

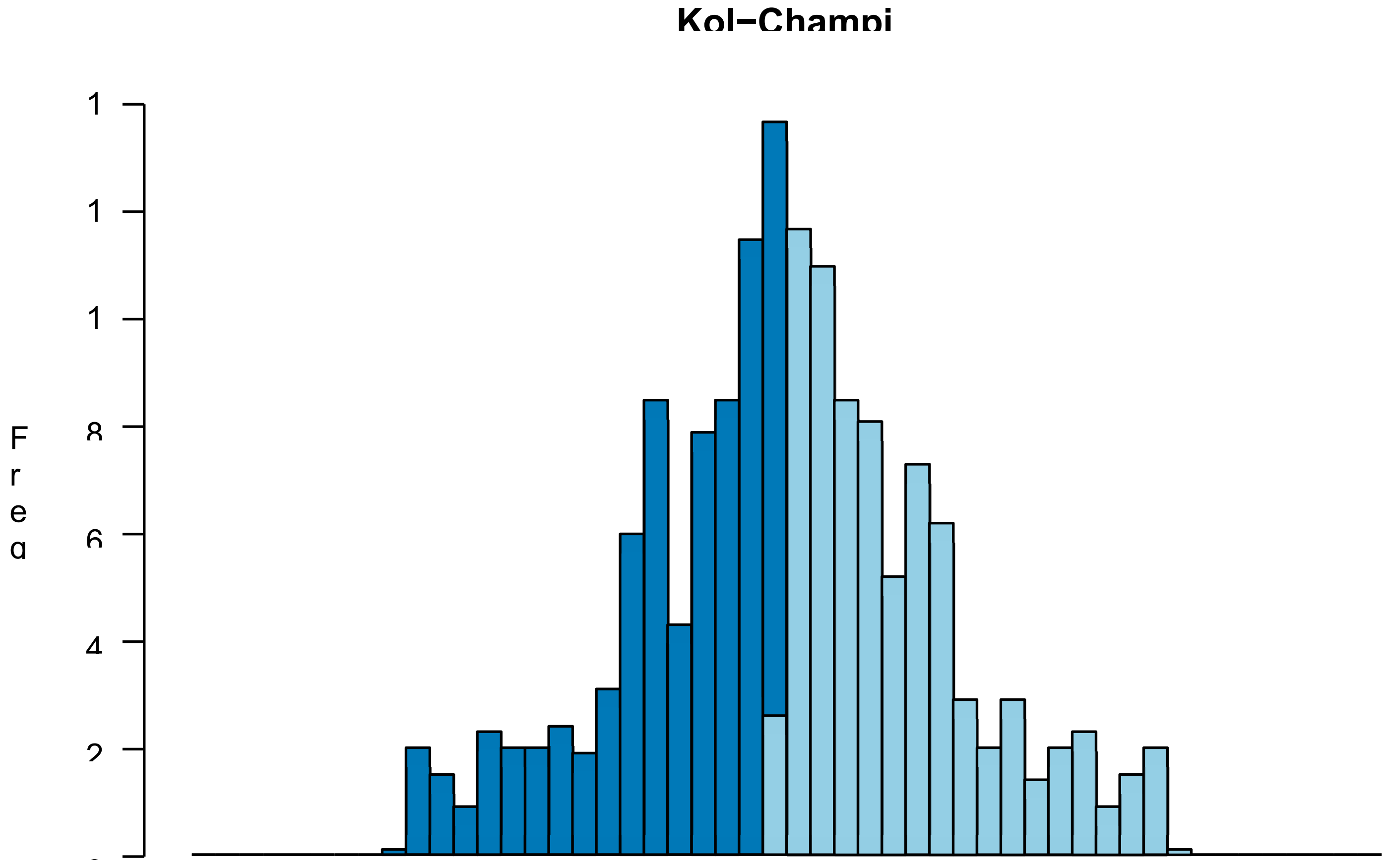

Kol−Champi
F
r
e
q
1
1
1
8
6
4
2

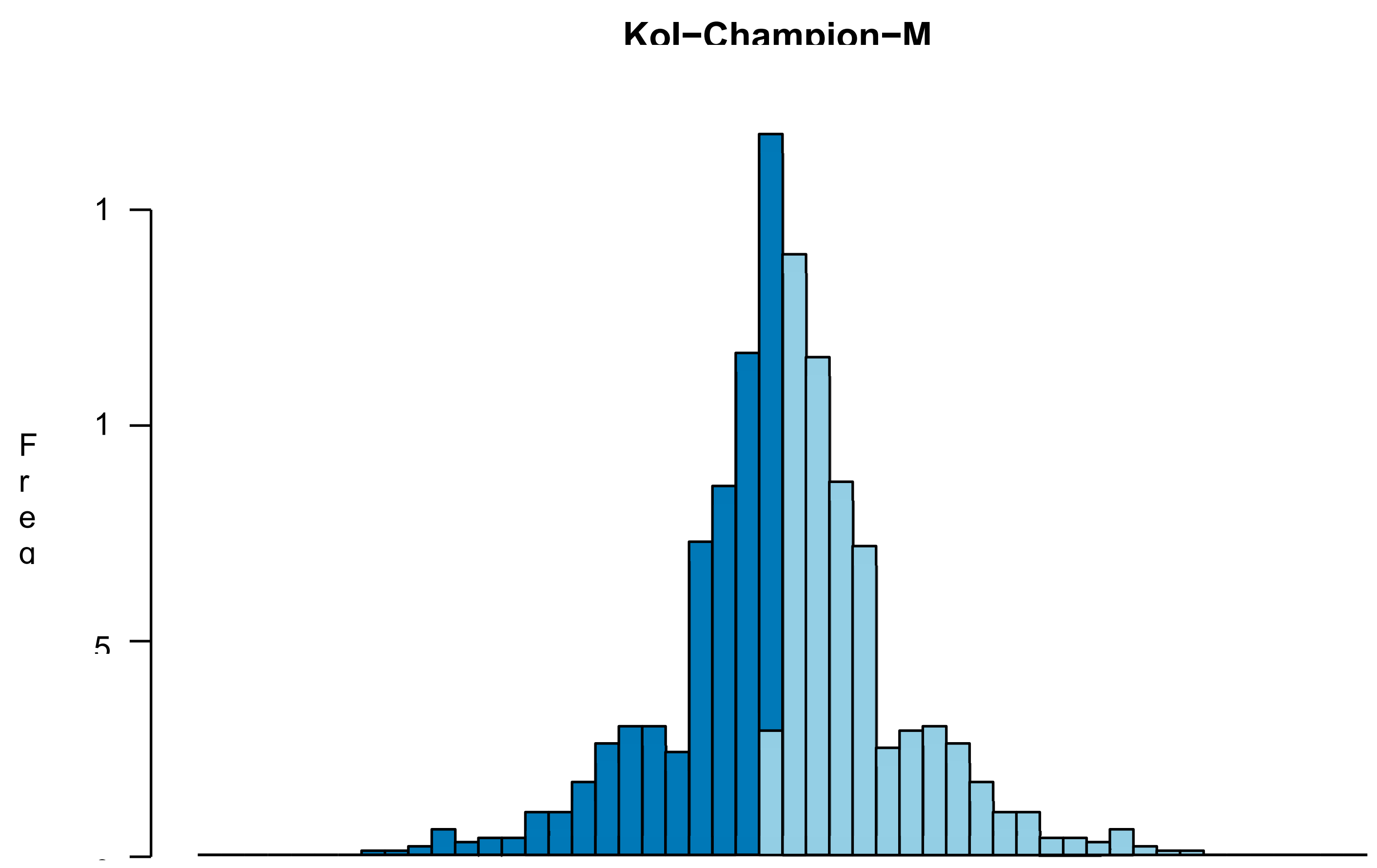
Kol−Champion−M
1
1
5
F
r
e
q

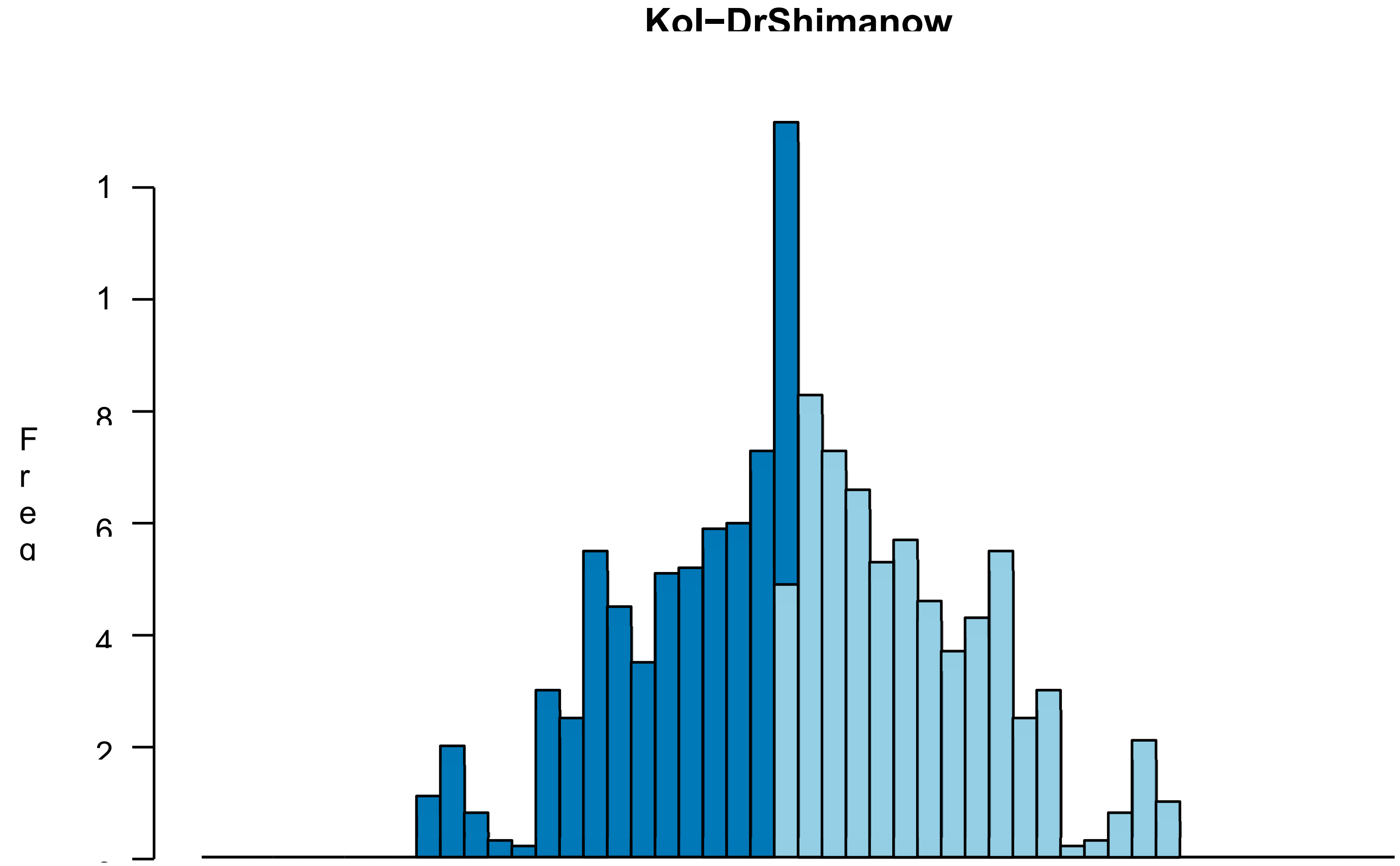
Kol−DrShimanow
F
r
e
q
1
1
8
6
4
2

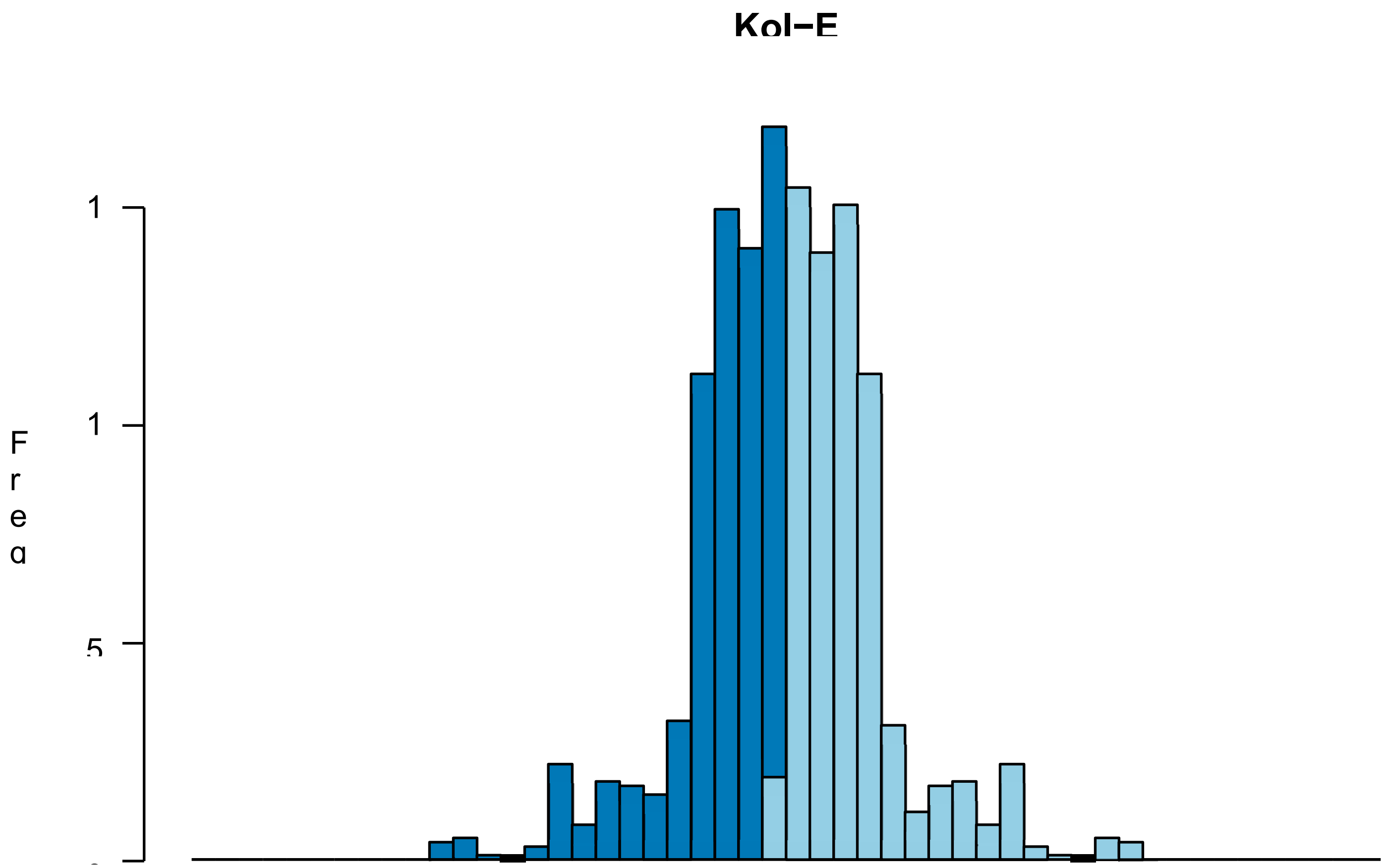

Kol−E
F
r
e
q
1
1
5

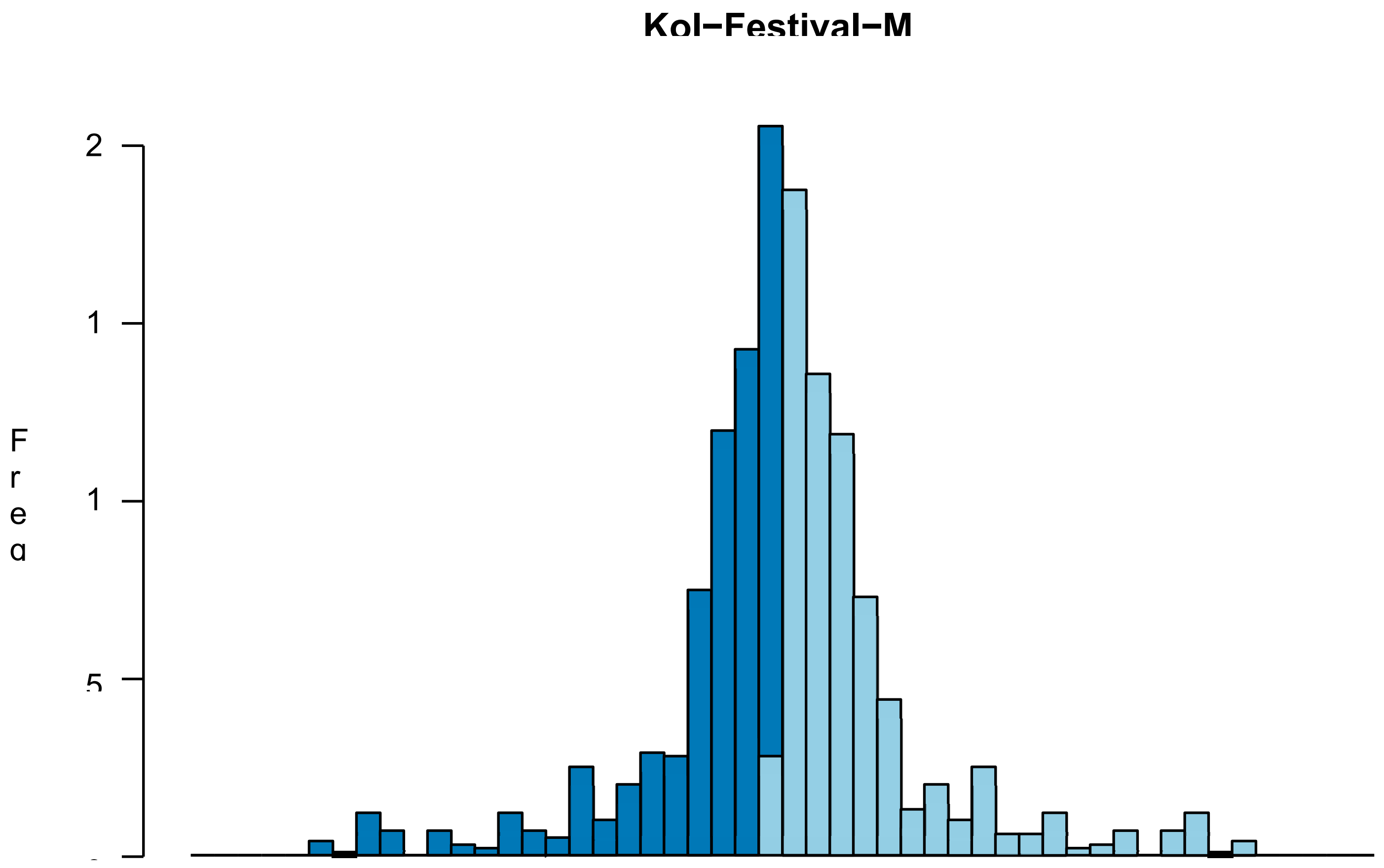

Kol−Festival−M
2
1
1
5
F
r
e
q

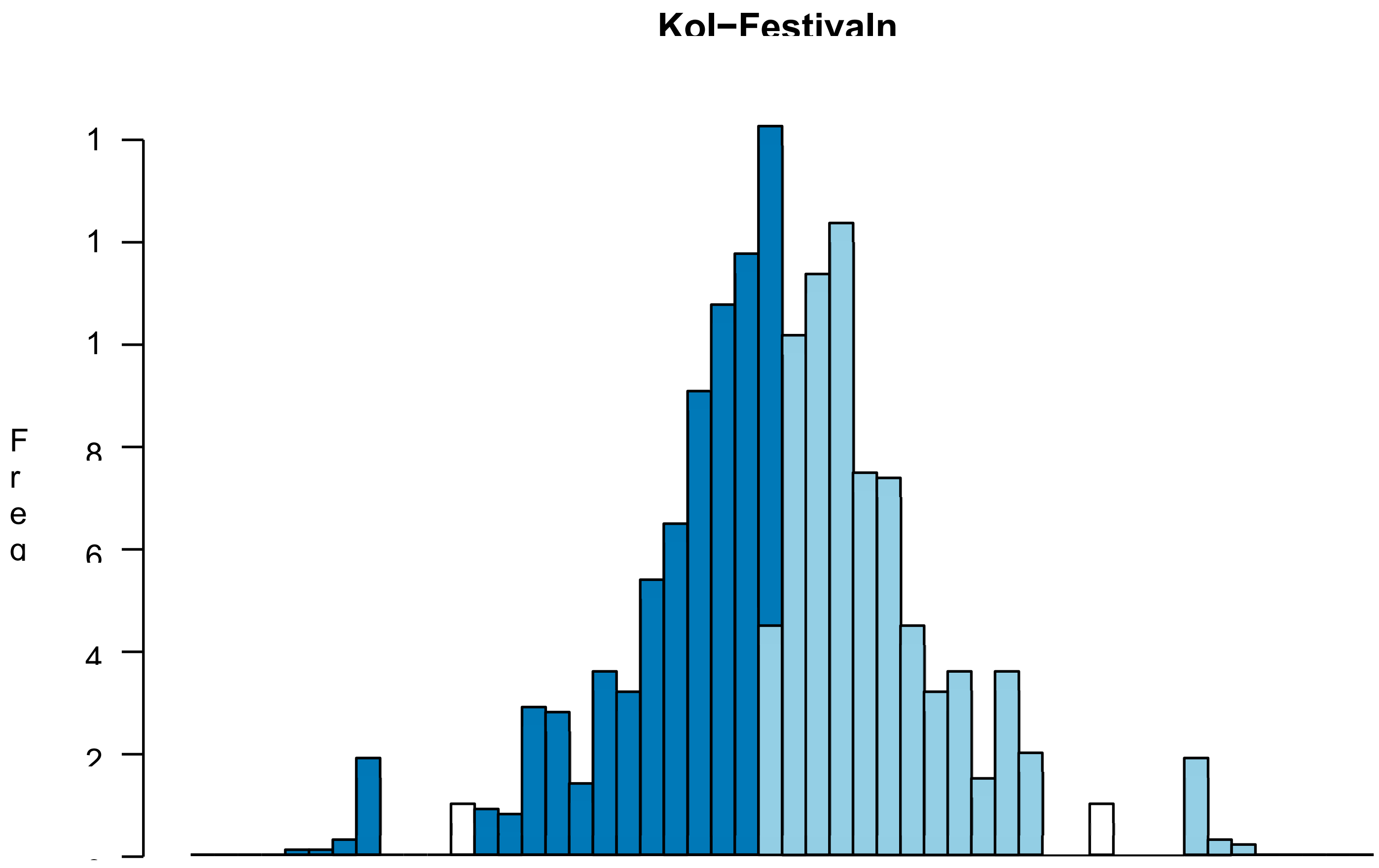

Kol−Festivaln
F
r
e
q
1
1
1
8
6
4
2

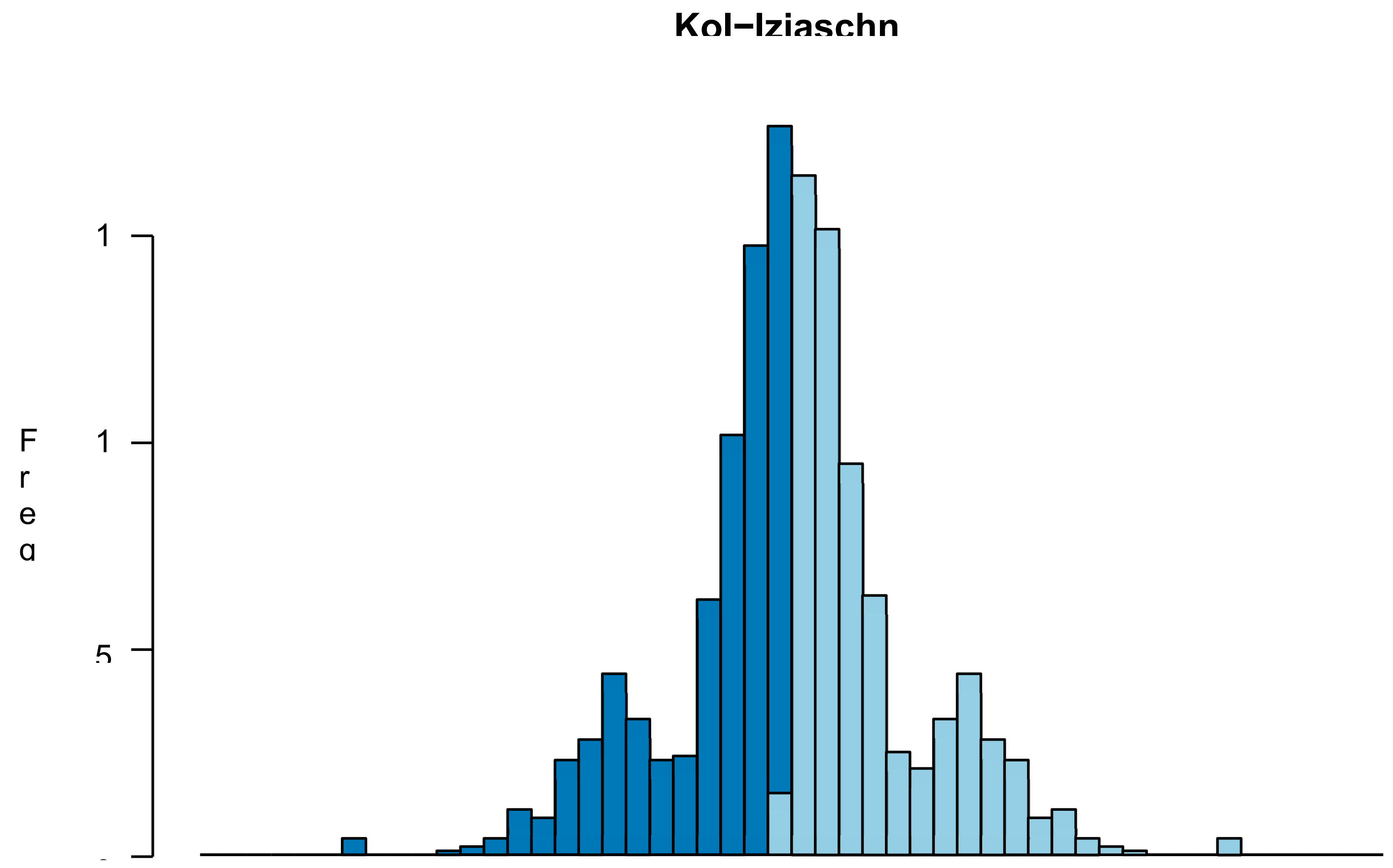
Kol−Iziaschn
F
r
e
q
1
1
5

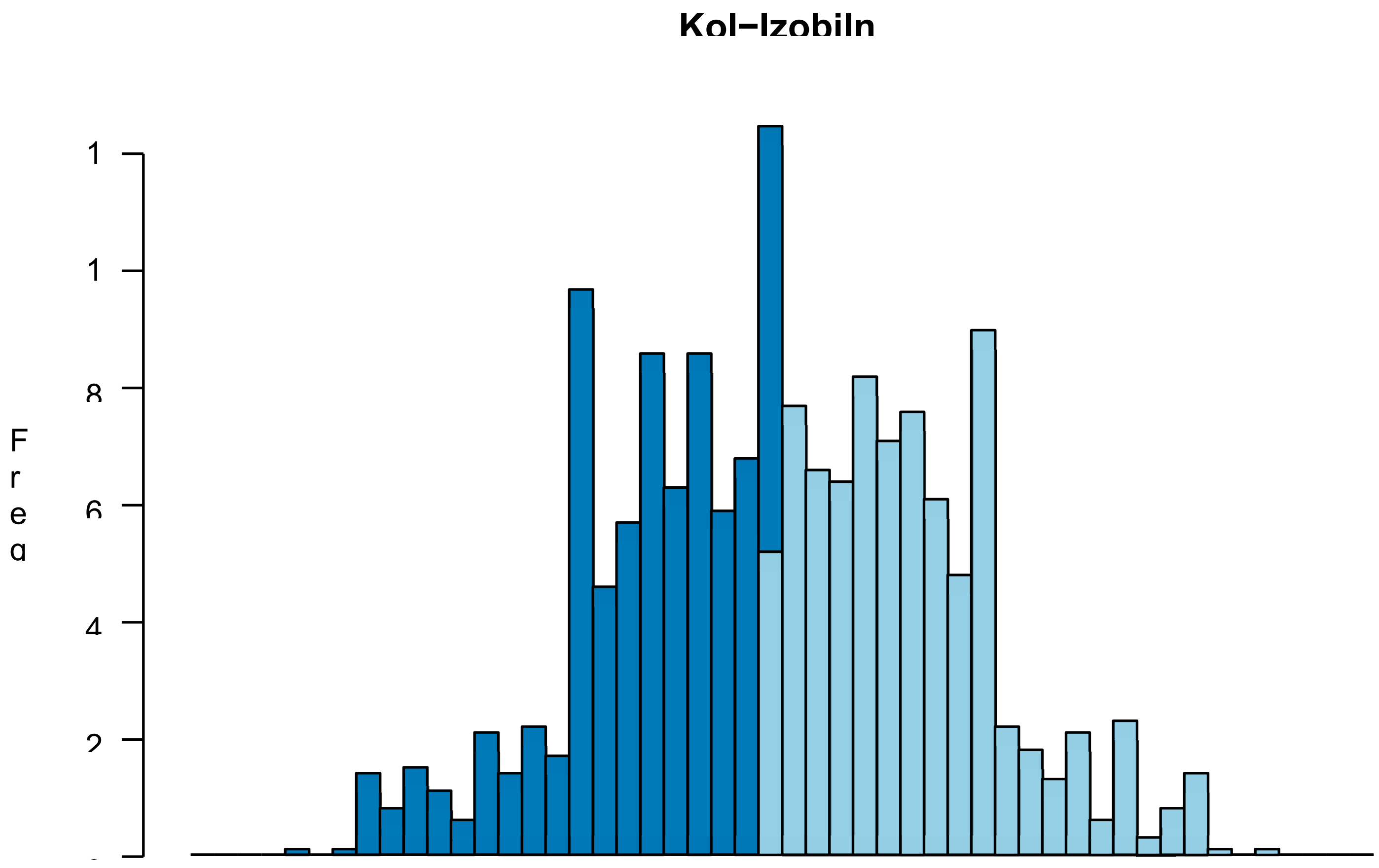

Kol–Izobiln
1
1
8
6
4
2
F
r
e
q

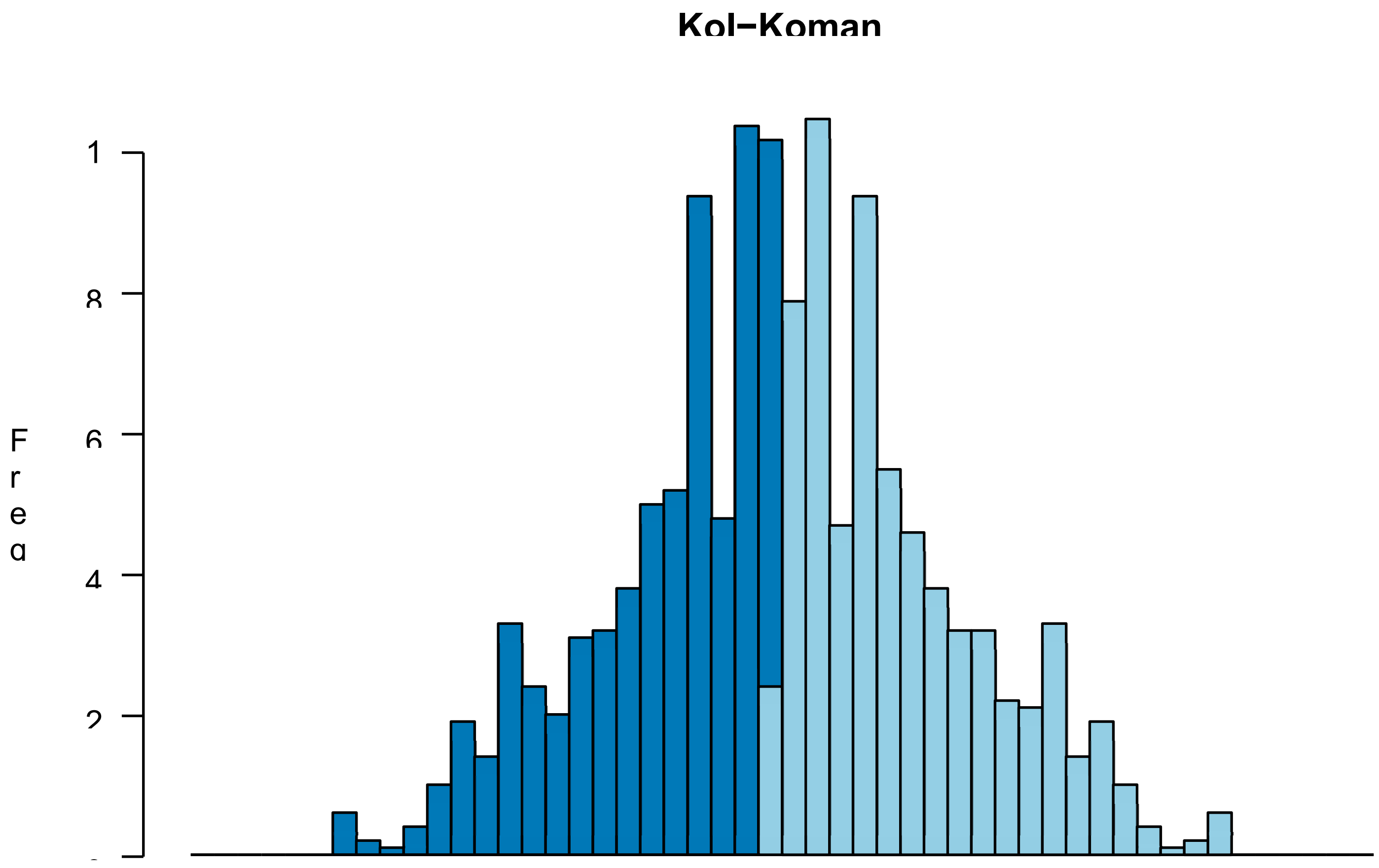
Kol−Koman
F
r
e
q
1
8
6
4
2

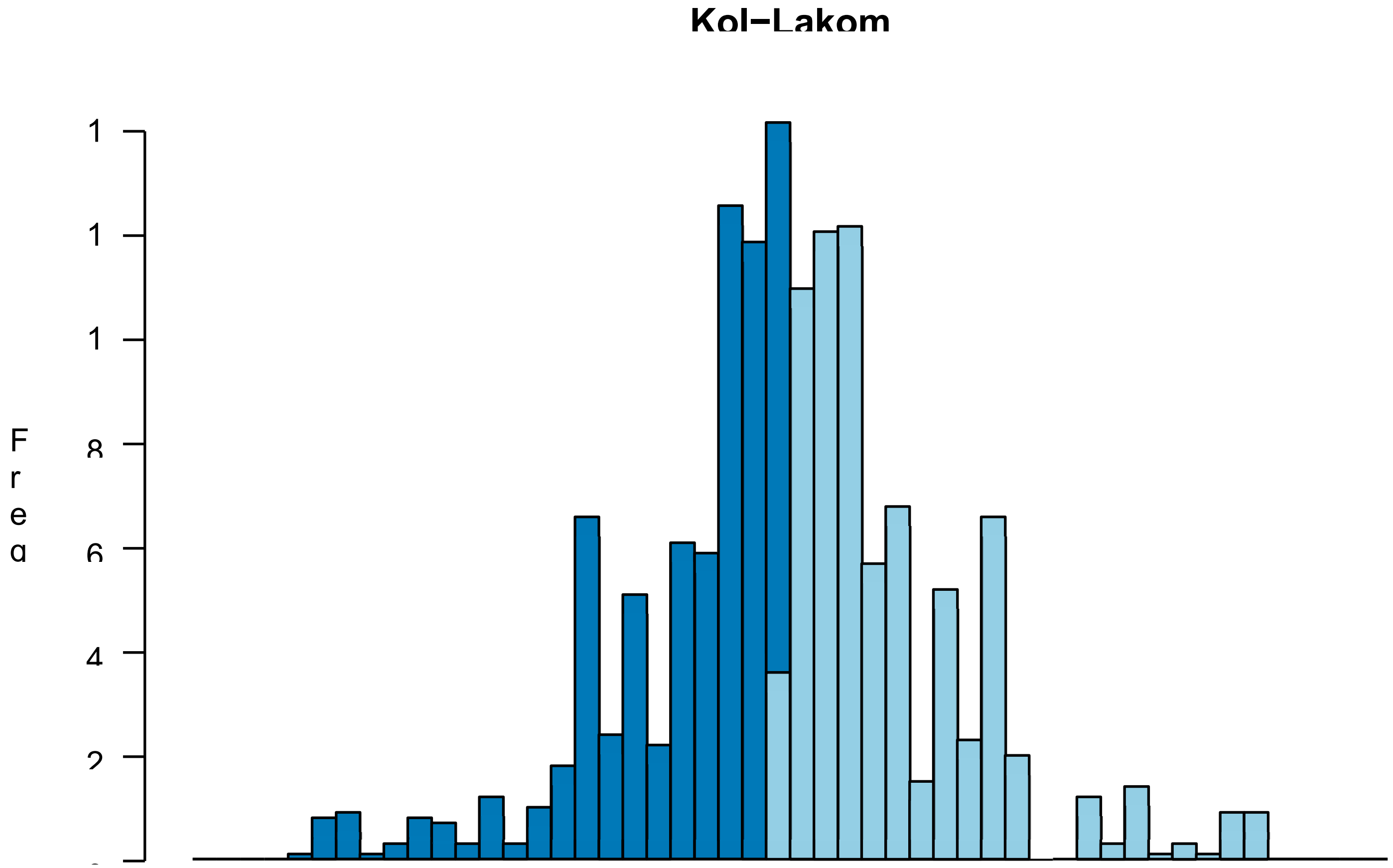

Kol−Lakom
F
r
e
q
1
1
1
8
6
4
2

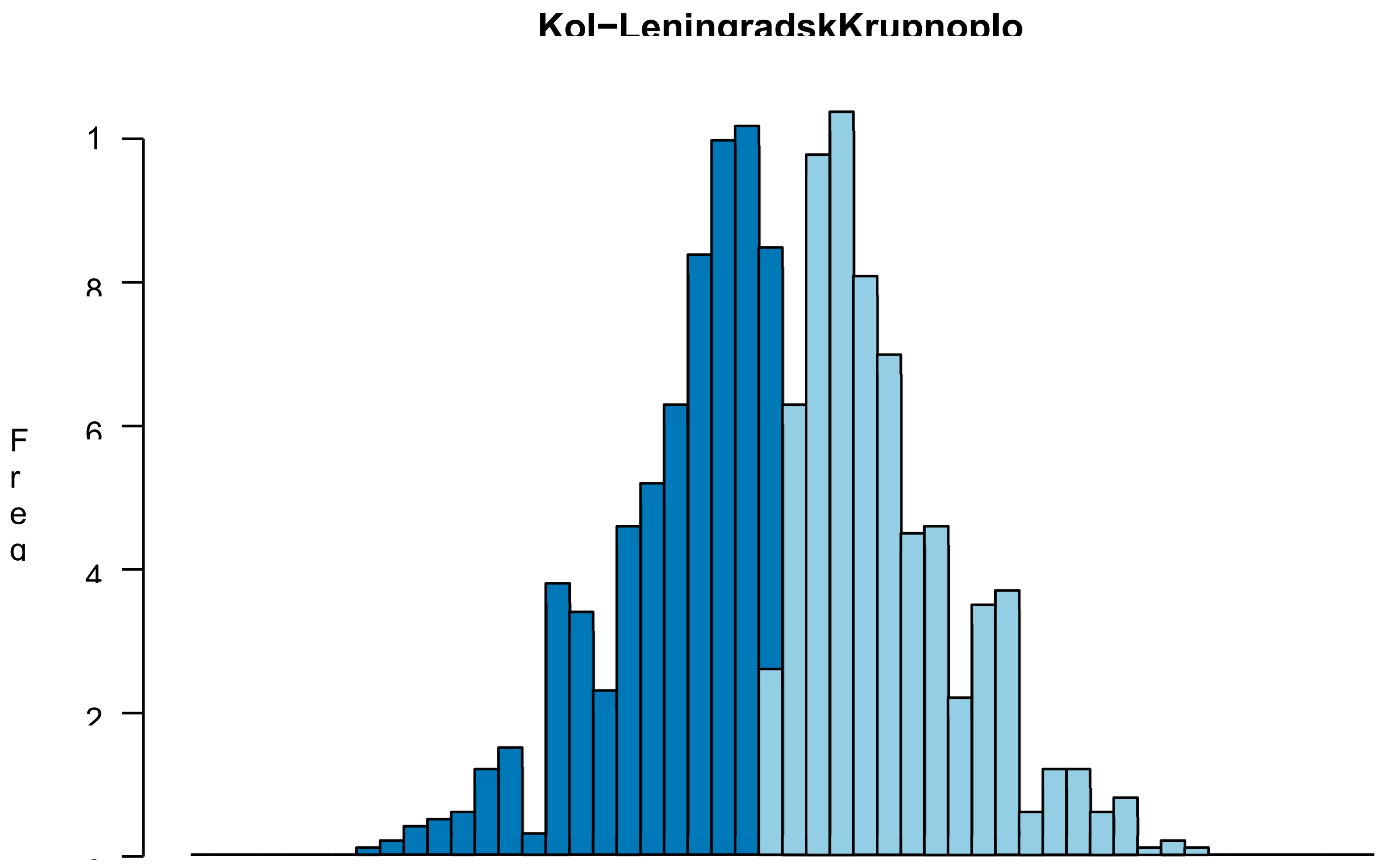

Kol−LeningradskKrupnoplo
F
r
e
q
1
8
6
4
2

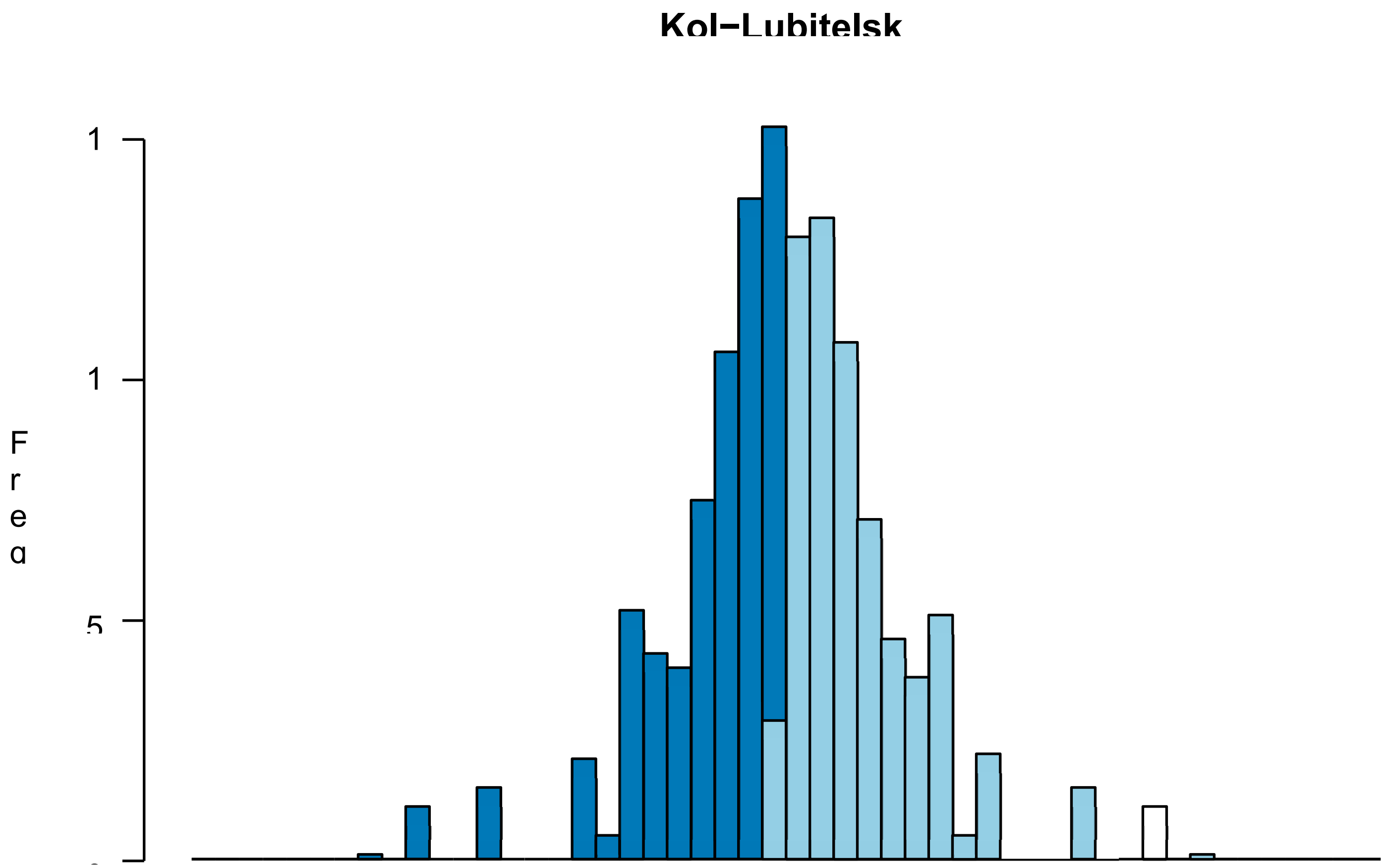

Kol−Lubitelsk
1
1
5
F
r
e
q

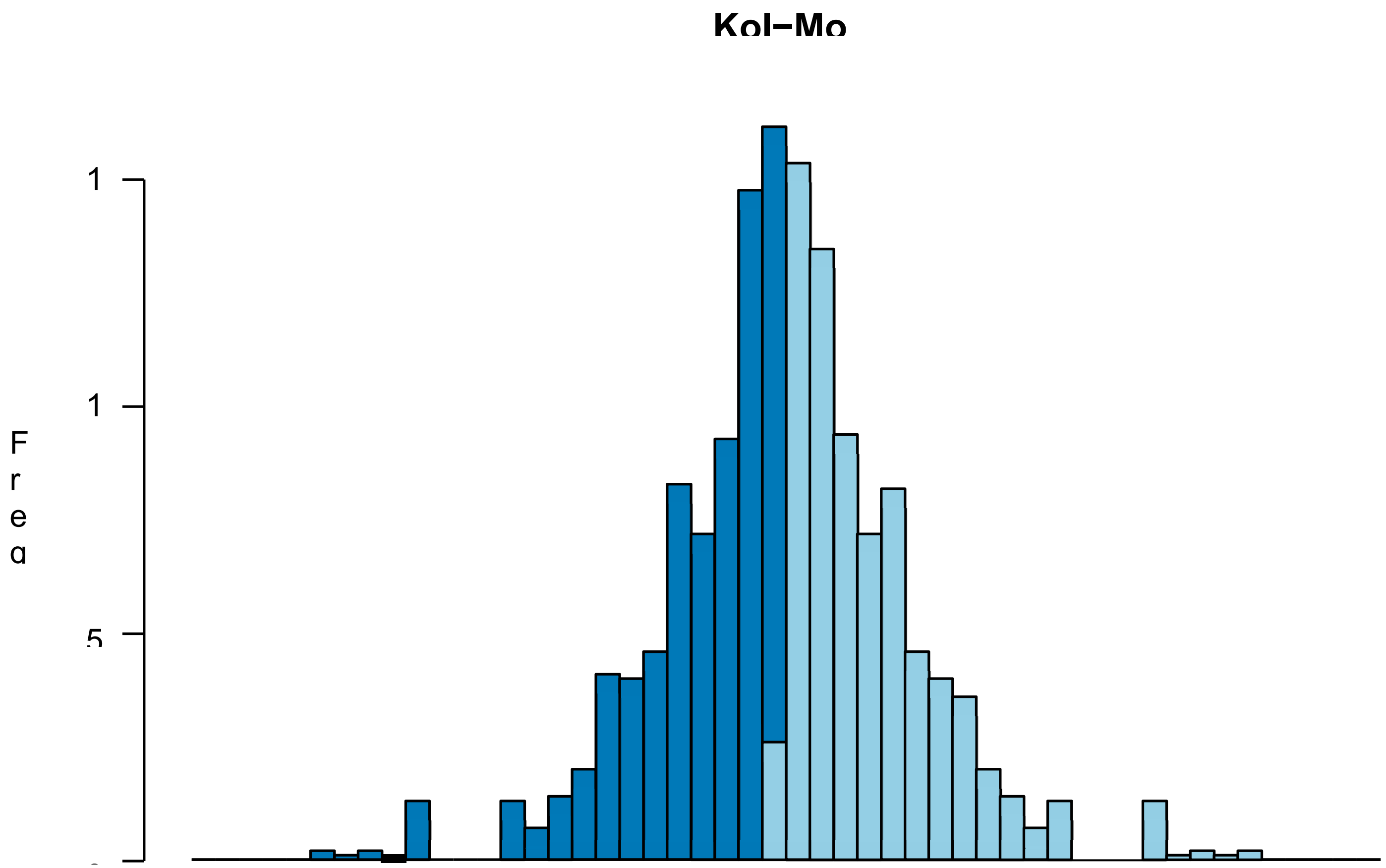

Kol−Mo
F
r
e
q
1
1
5

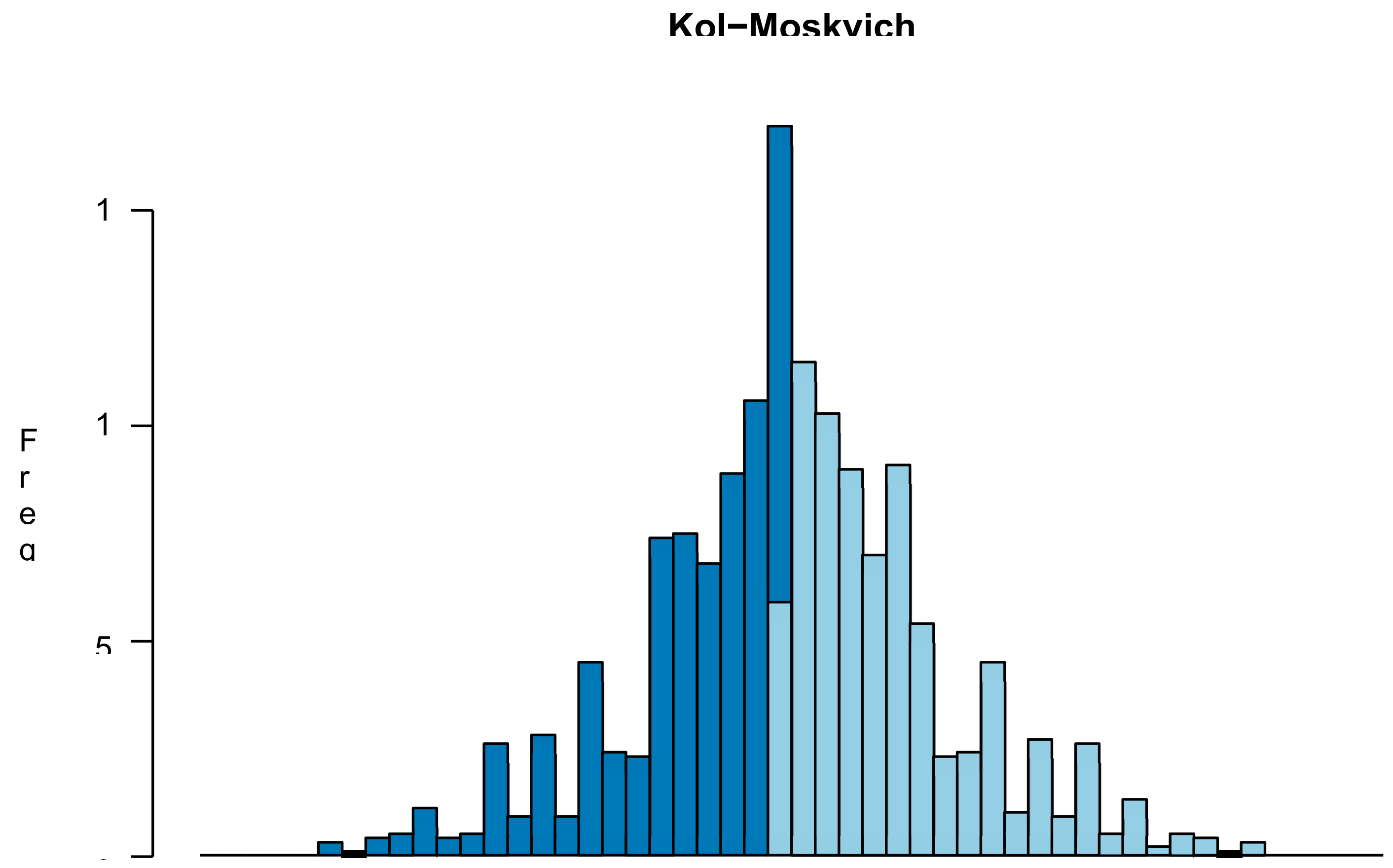
Kol−Moskvich
F
r
e
q
1
1
5

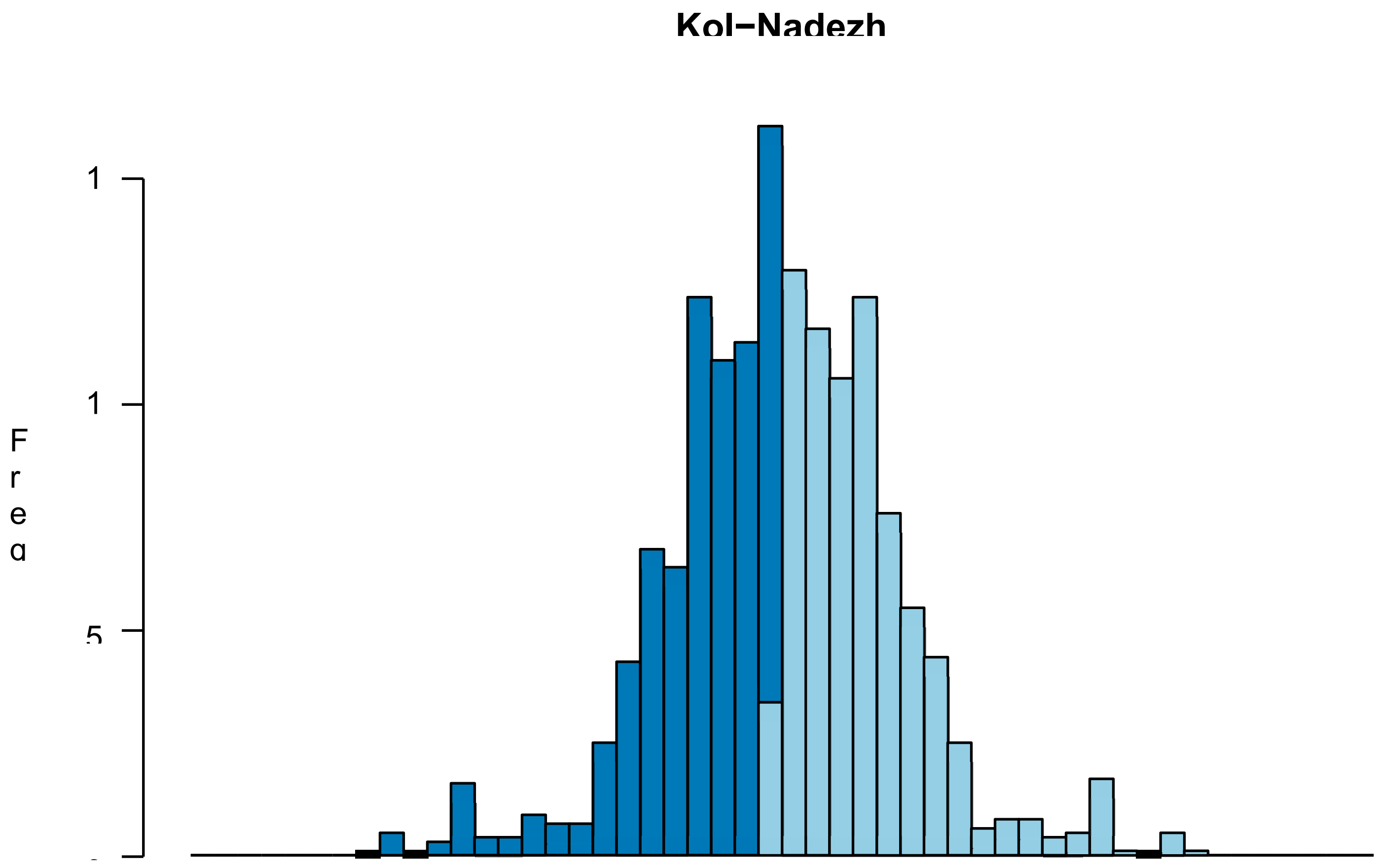

Kol−Nadezh
1
1
5
F
r
e
q

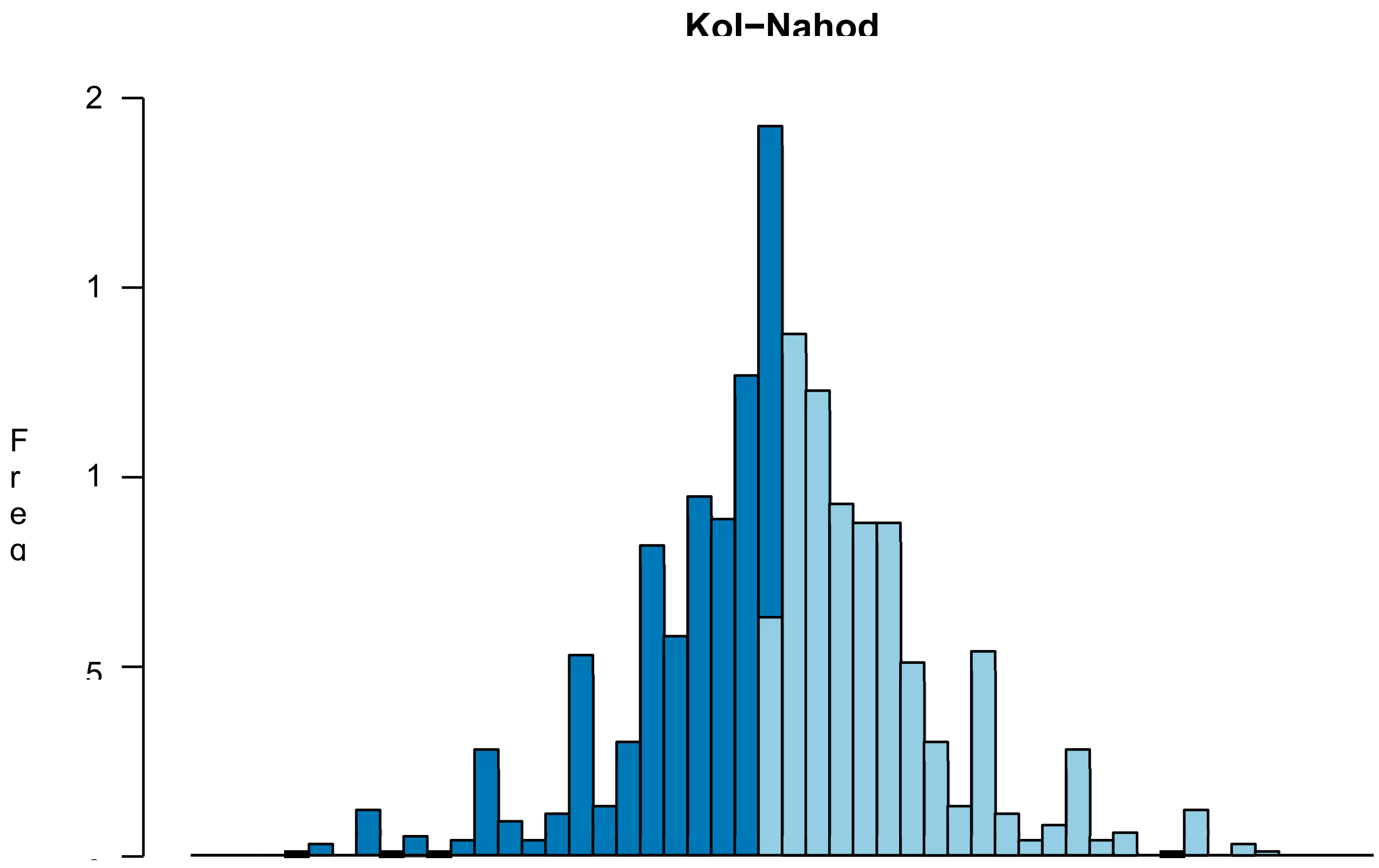

Kol−Nahod
2
1
1
5
F
r
e
q

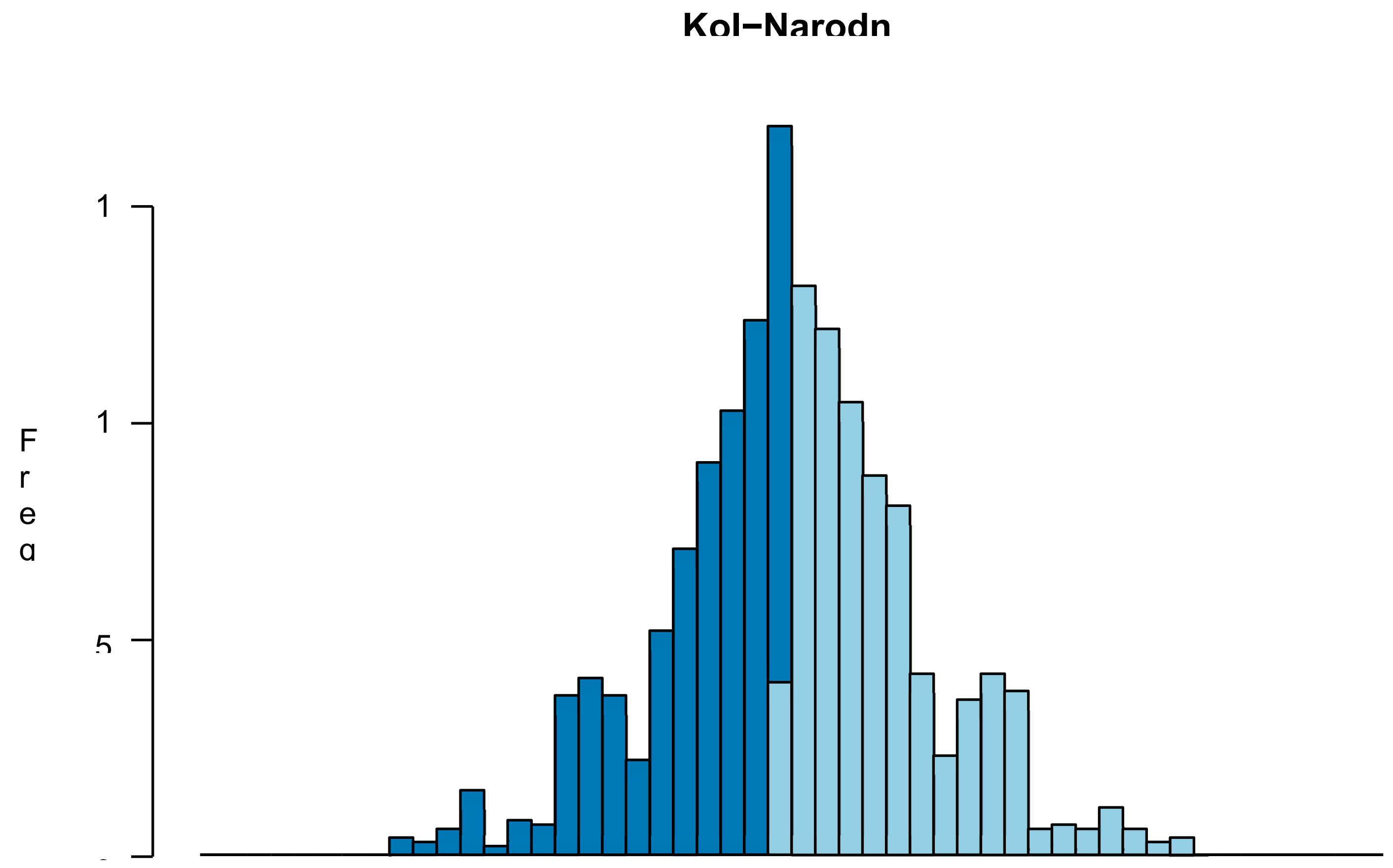

Kol−Narodn
F
r
e
q
1
1
5

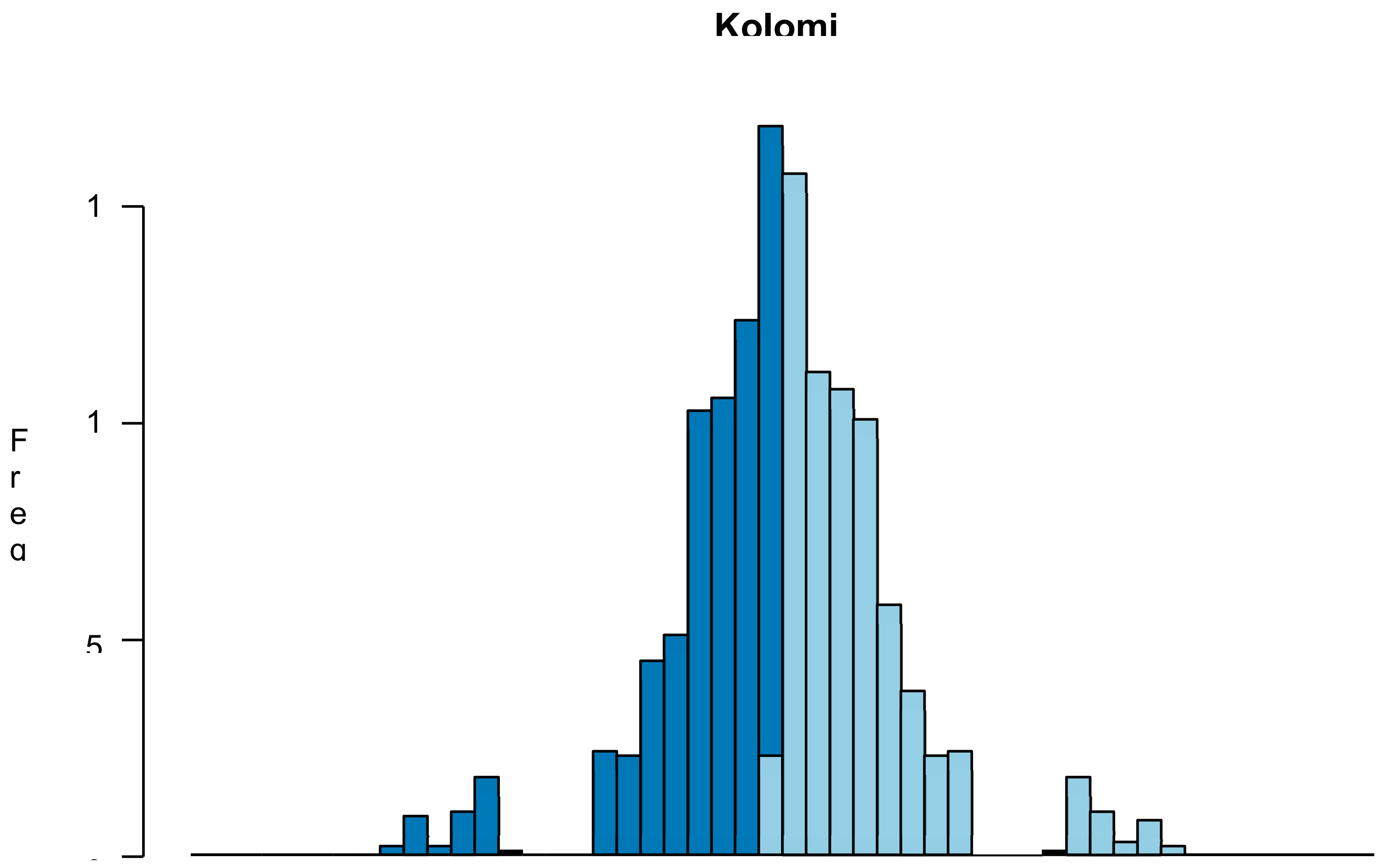

Kolomi
1
1
5
F
r
e
q

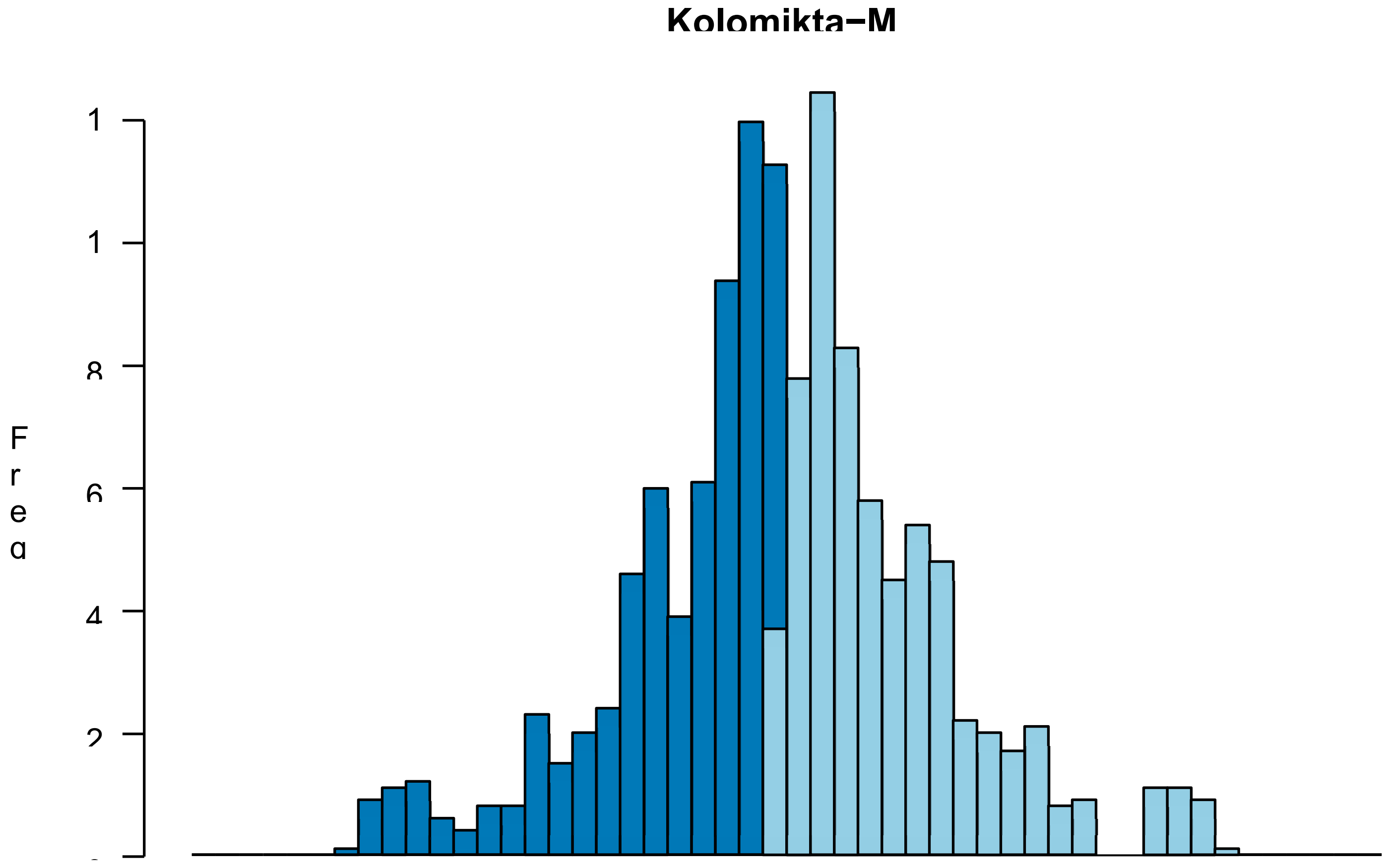

Kolomikta−M
1
1
8
6
4
2
F
r
e
q

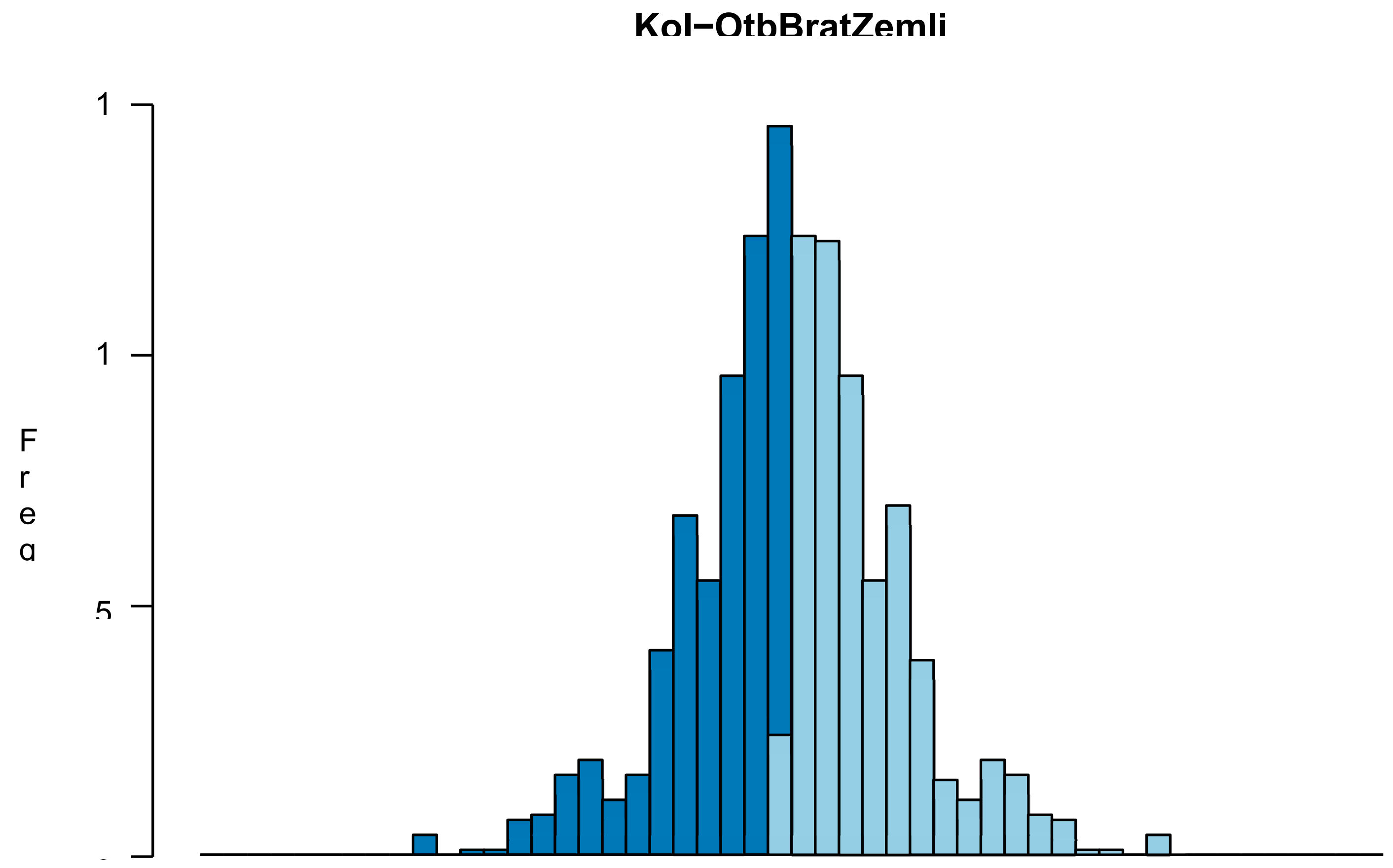

Kol−OtbBratZemli
1
1
5
F
r
e
q

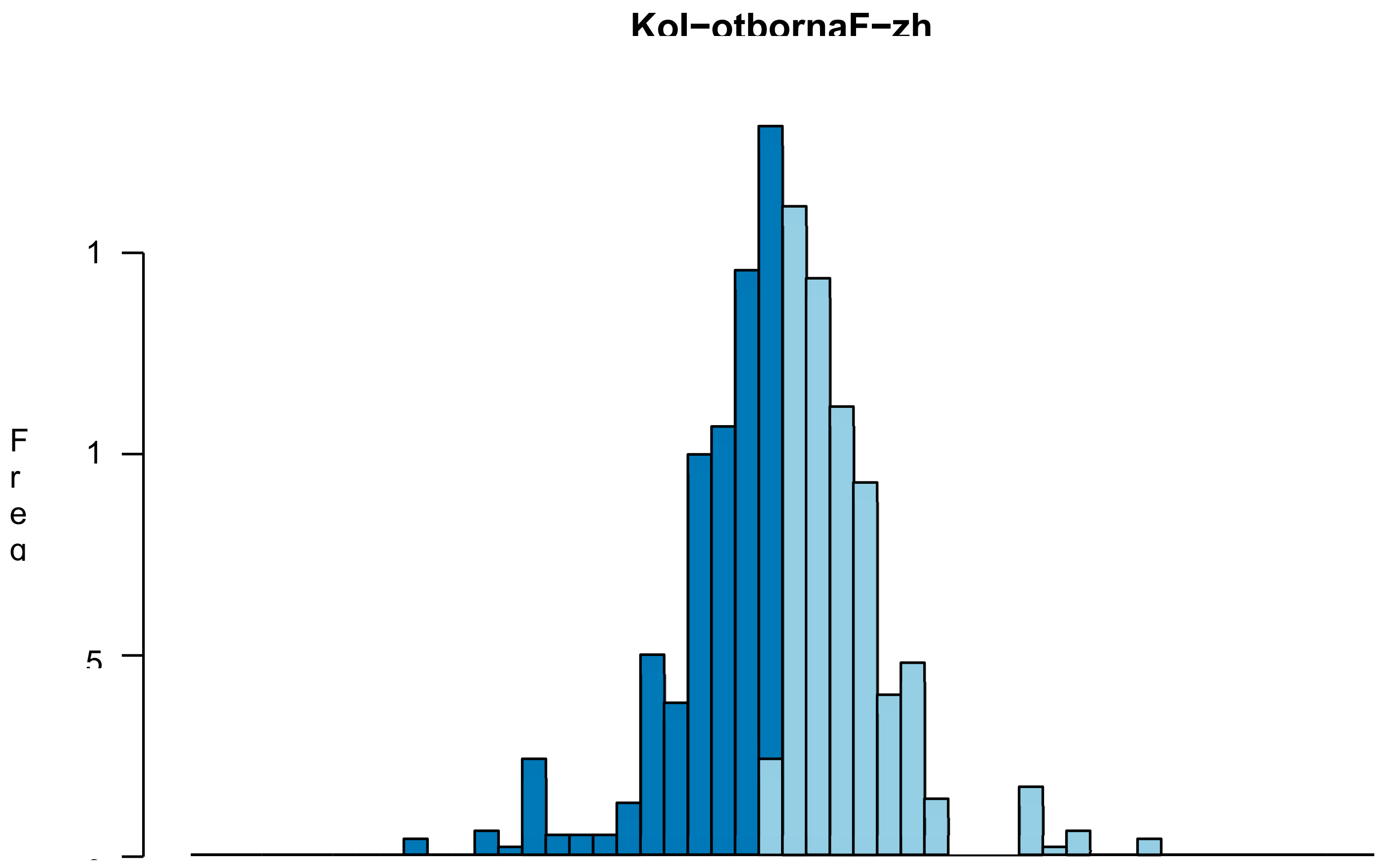

Kol−otbornaF−zh
F
r
e
q
1
1
5

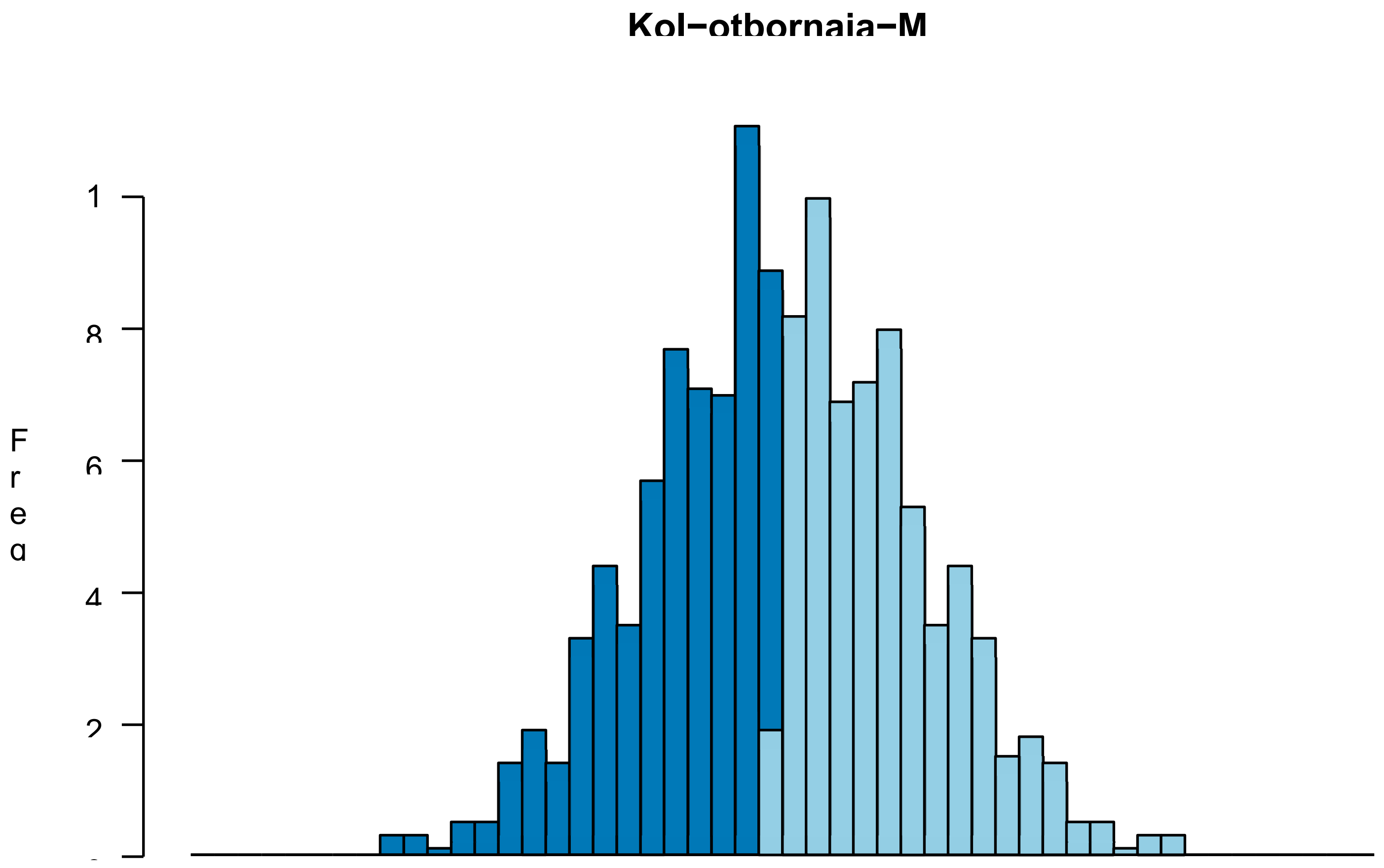
Kol−otbornaia−M
F
r
e
q
1
8
6
4
2

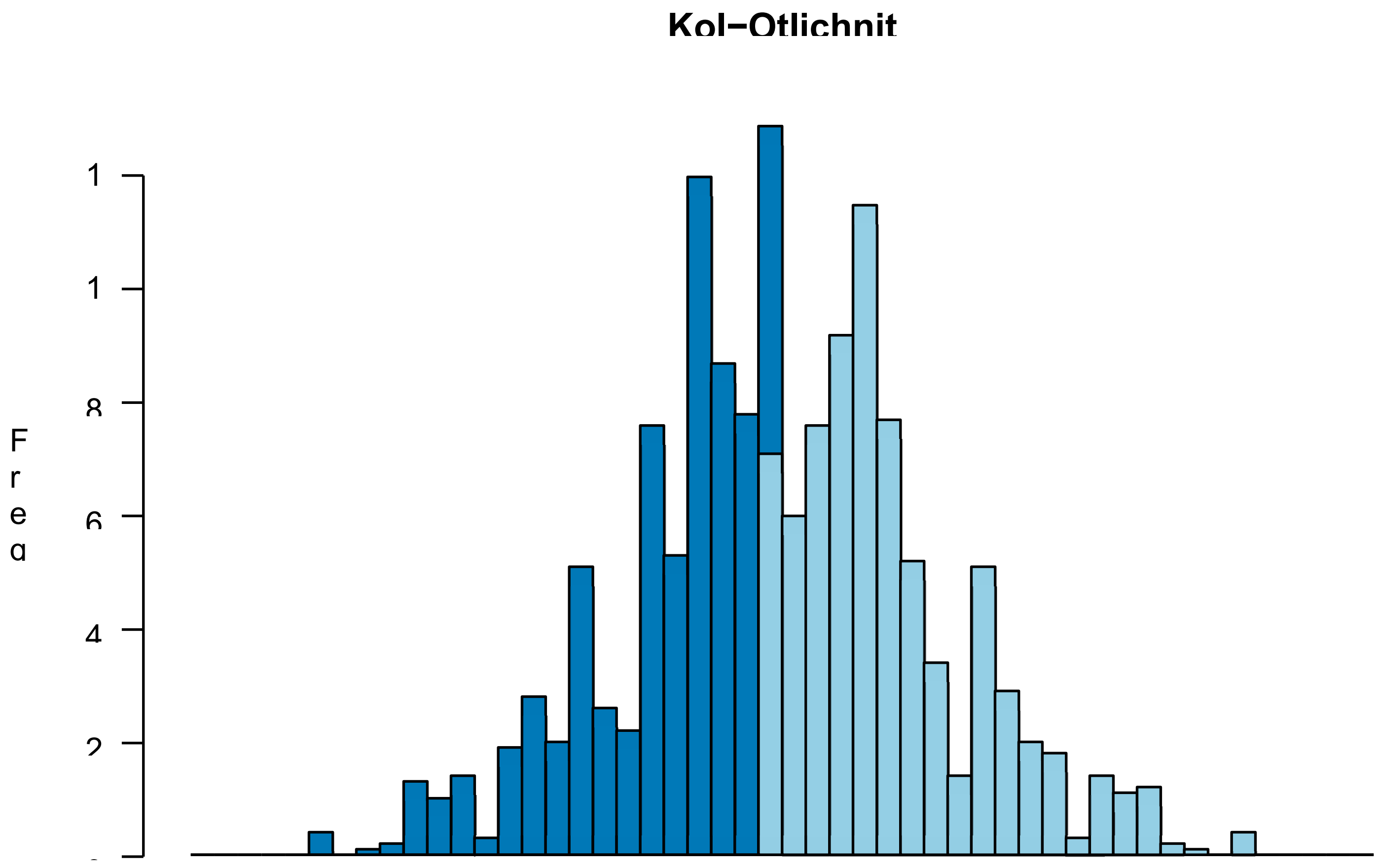

Kol−Otlichnit
1
1
8
F
r
e
q
6
4
2

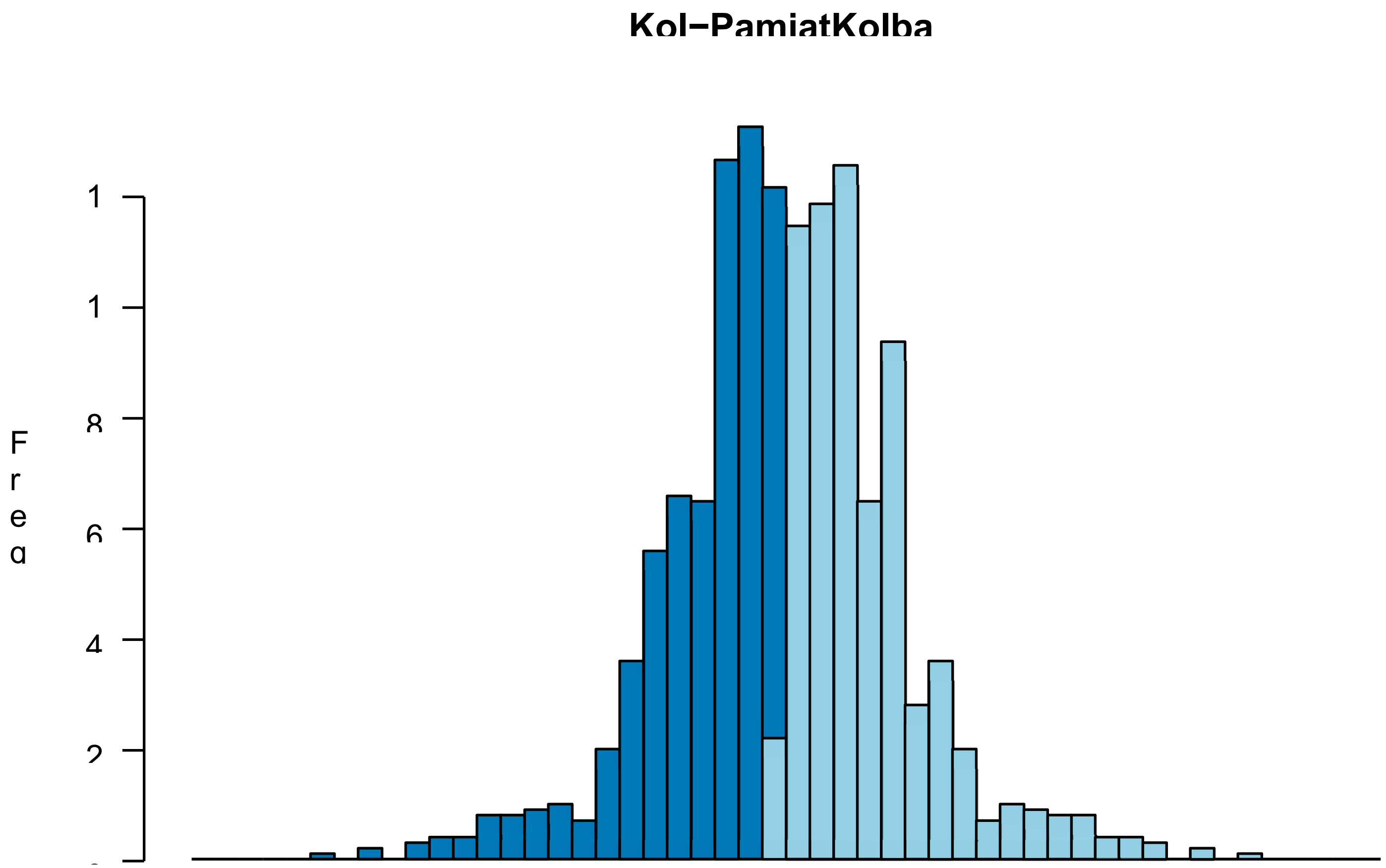

Kol−PamiatKolba
F
r
e
q
1
1
8
6
4
2

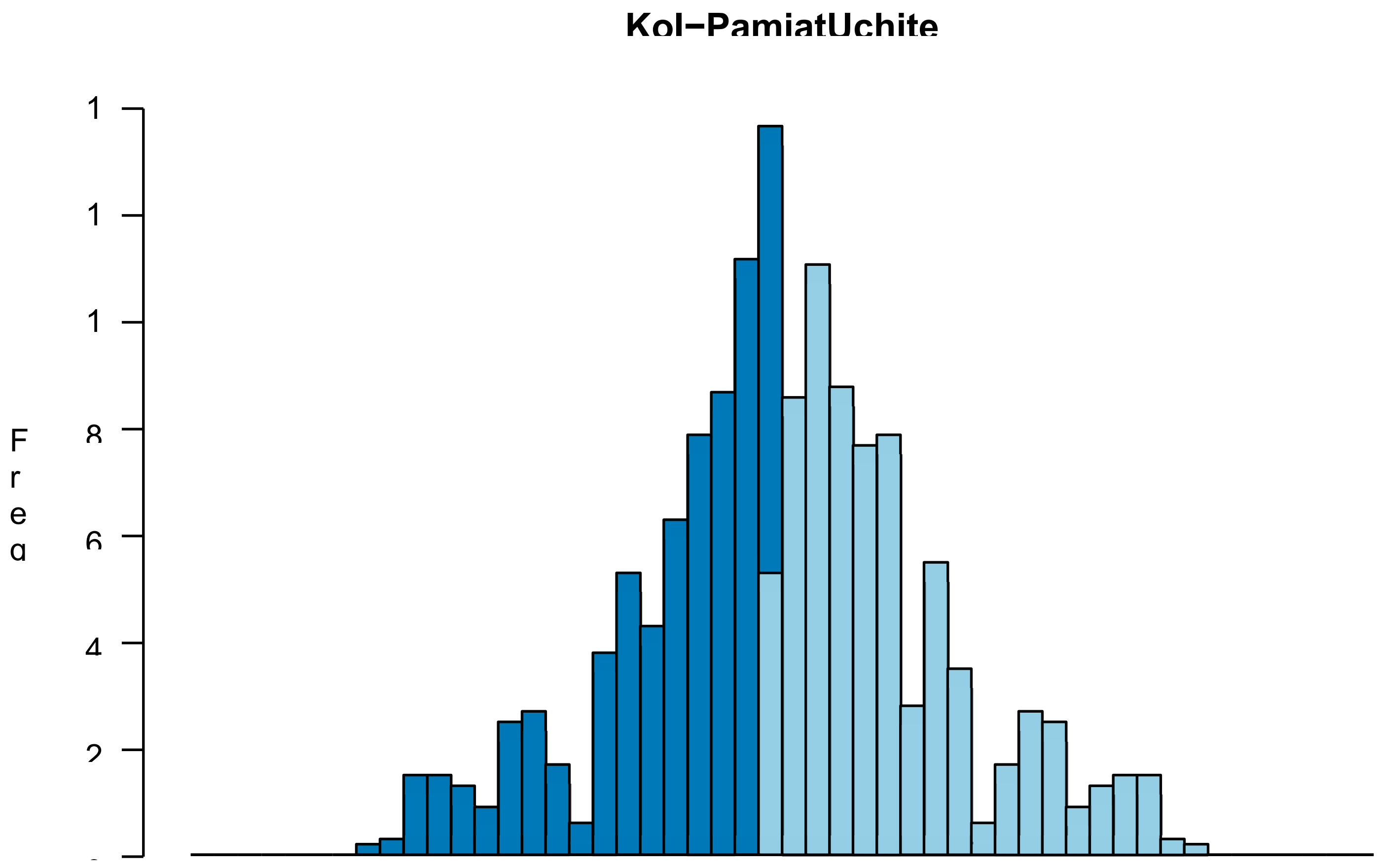

Kol−PamiatUchite
F
r
e
q
1
1
1
8
6
4
2

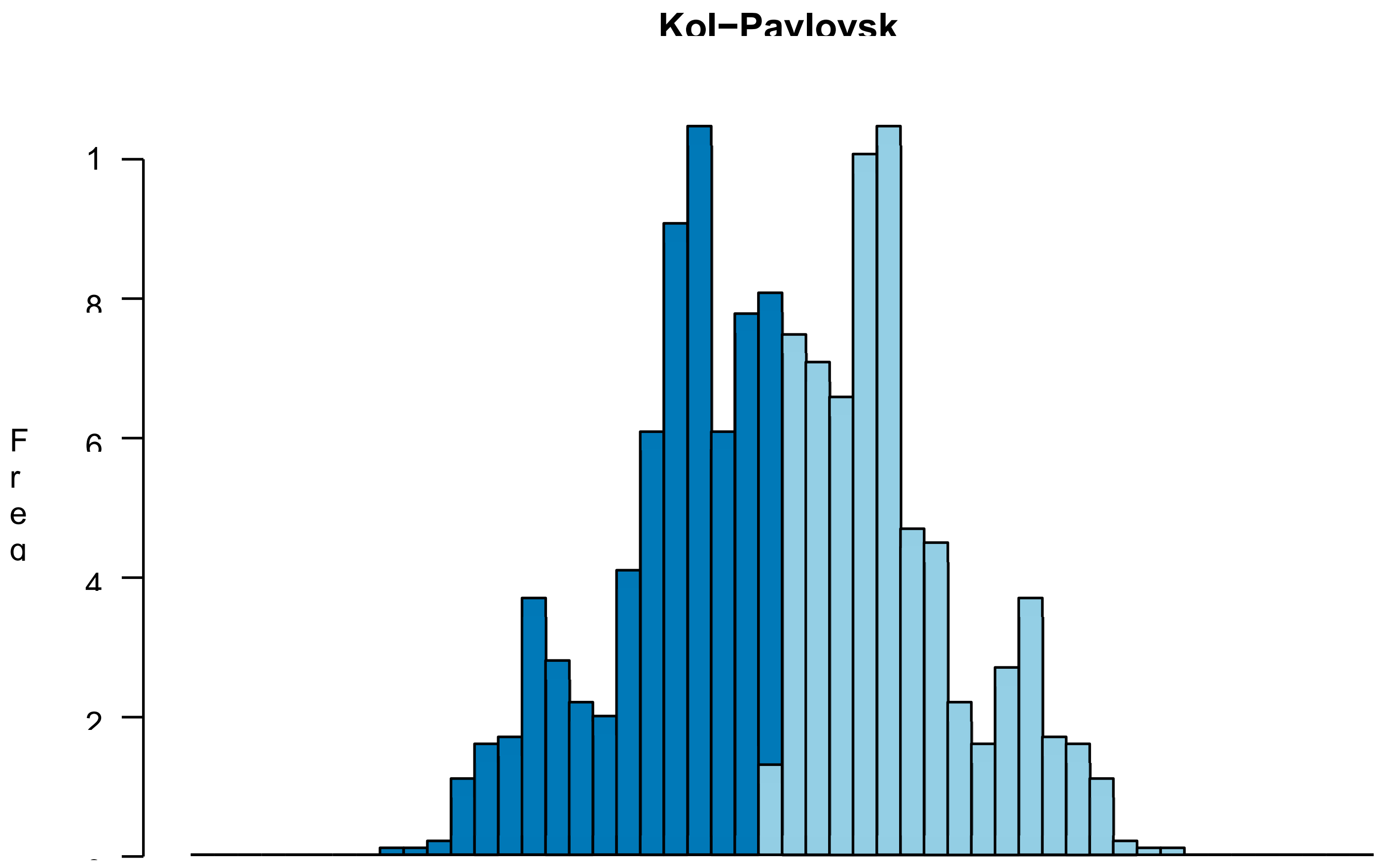
Kol−Pavlovsk
F
r
e
q
1
8
6
4
2

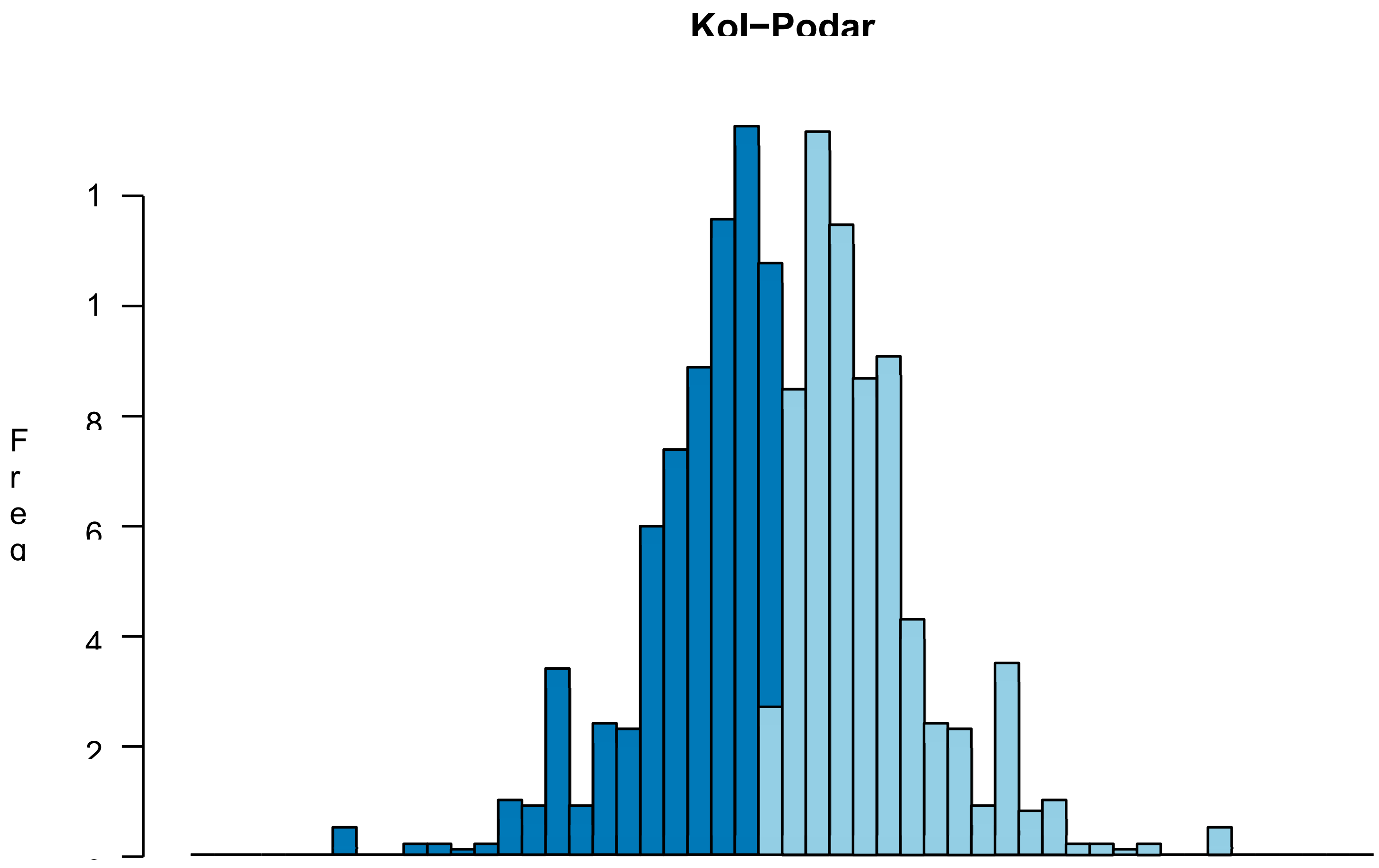

Kol−Podar
F
r
e
q
1
1
8
6
4
2

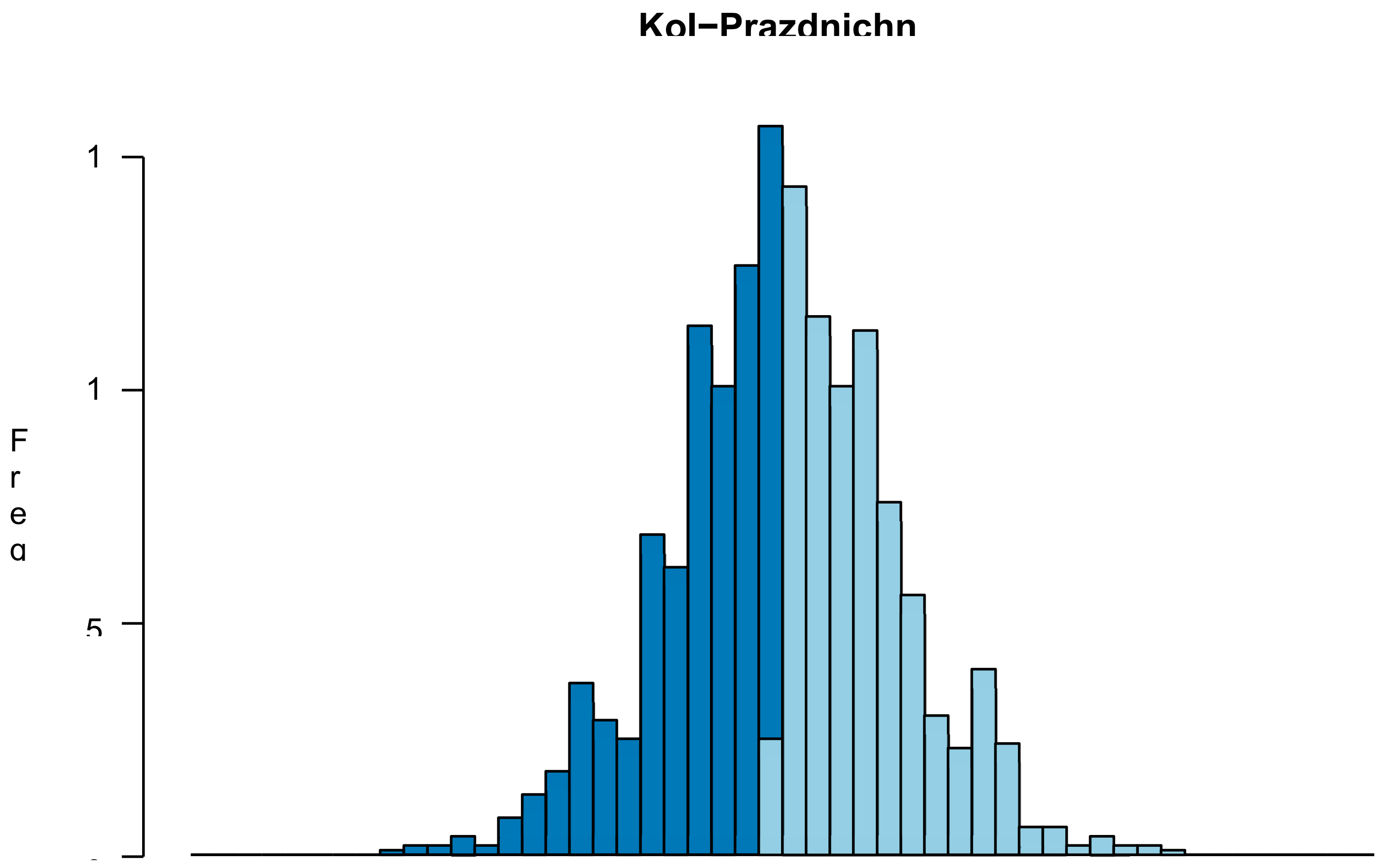
Kol−Prazdnichn
1
1
5
F
r
e
q

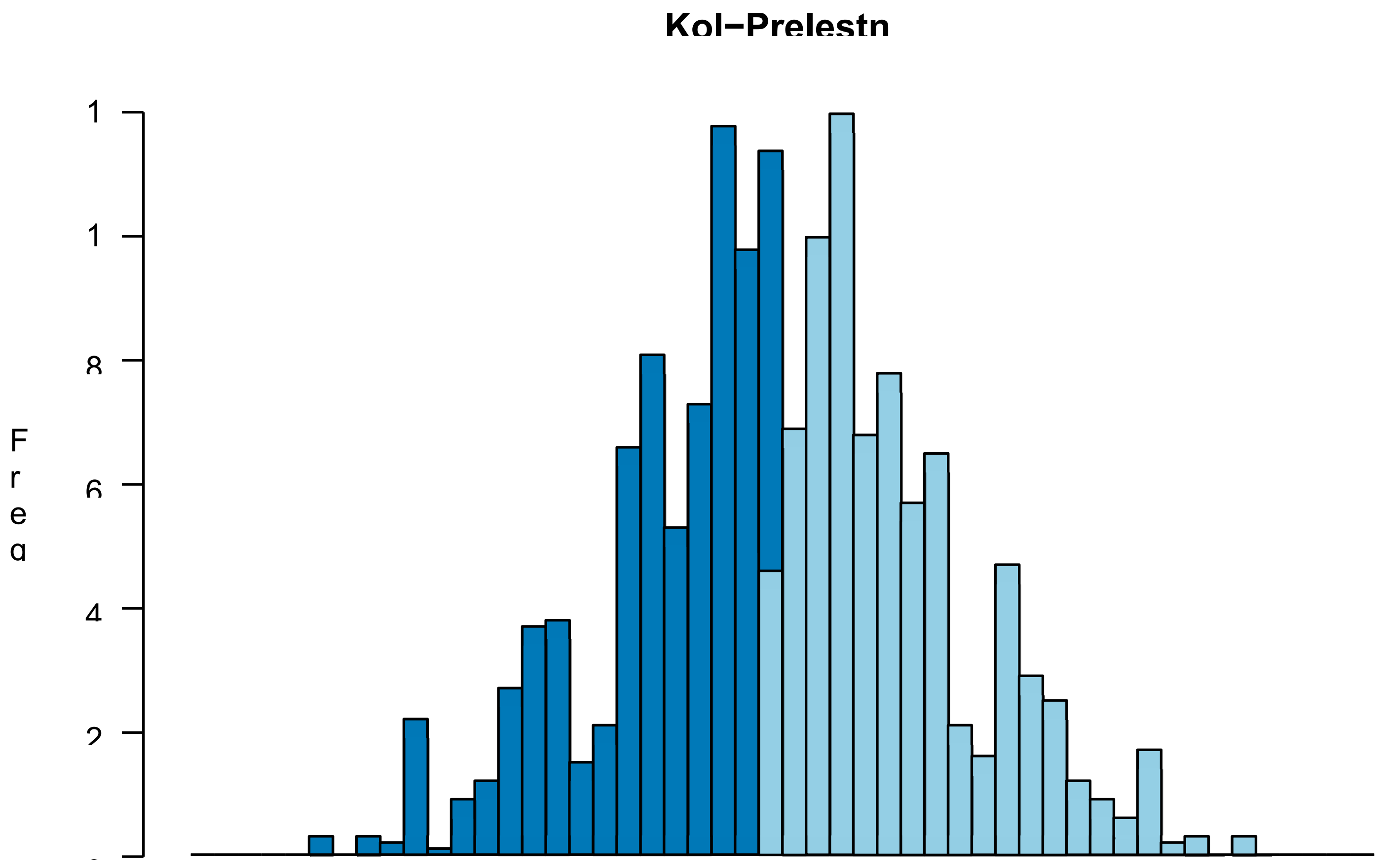

Kol−Prelestn
1
1
8
6
4
2
F
r
e
q

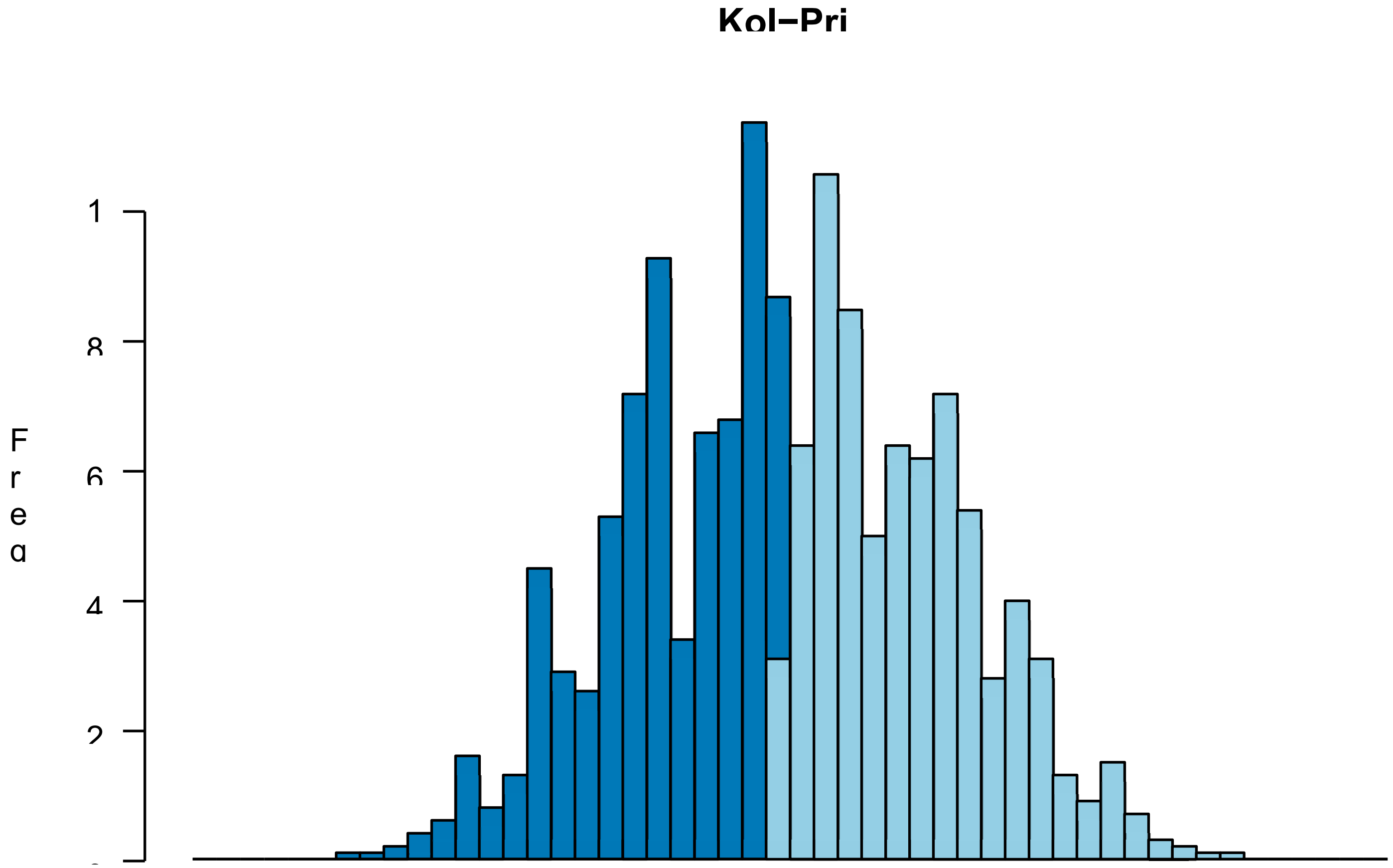
Kol−Pri
1
8
6
4
2
F
r
e
q

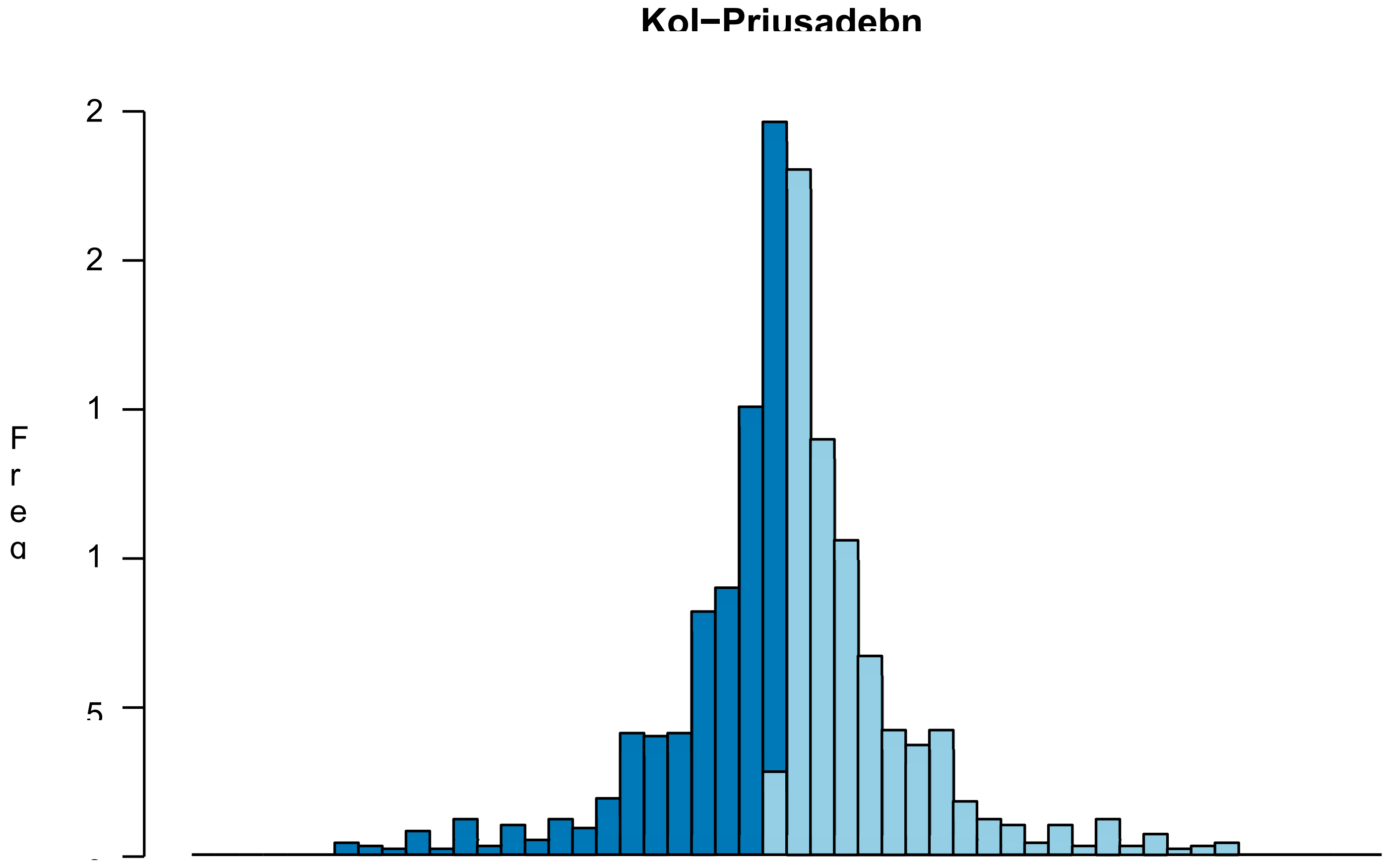

Kol−Priusadebn
F
r
e
q
2
2
1
1
5

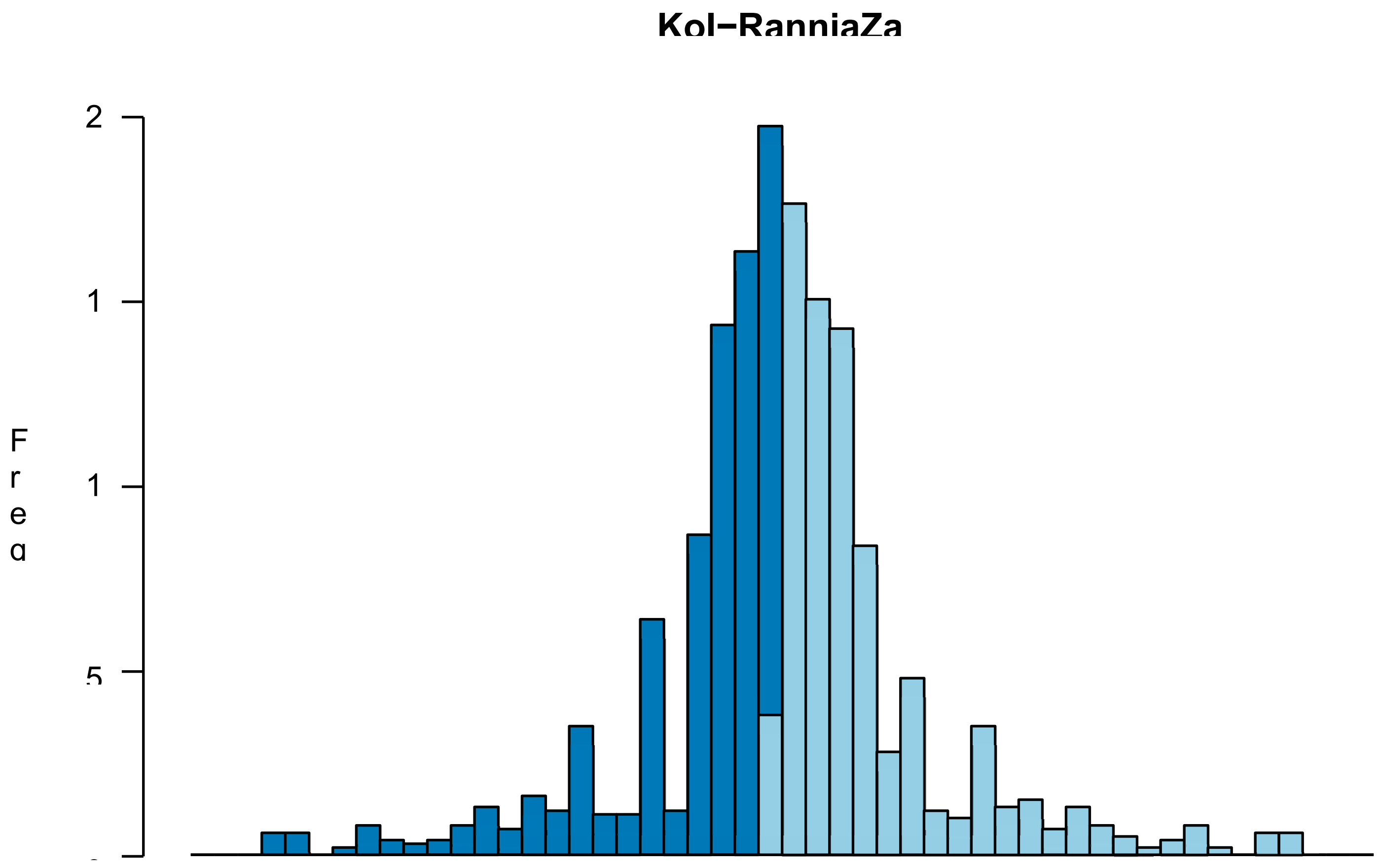
Kol−RanniaZa
F
r
e
q
2
1
1
5

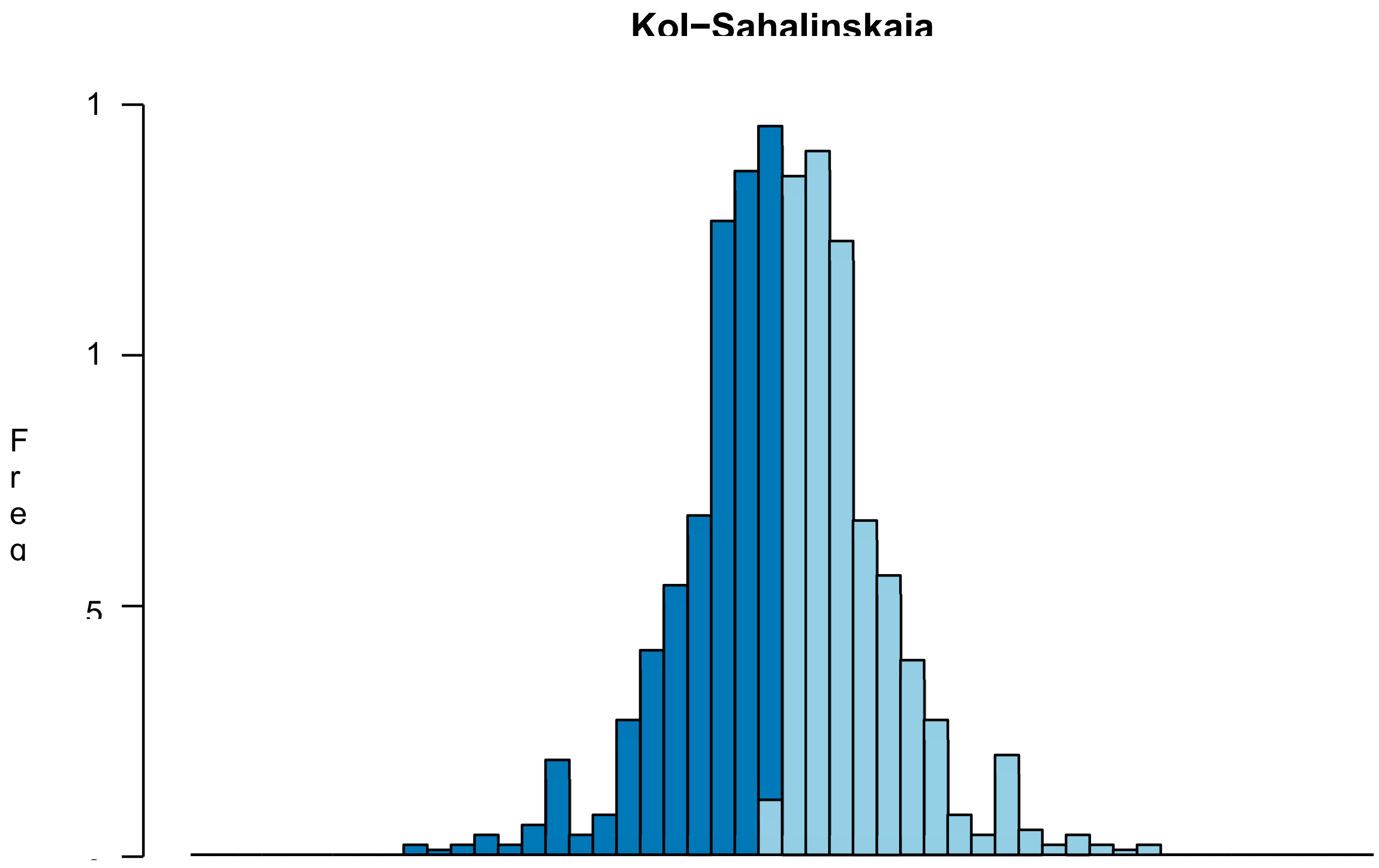
Kol−Sahalinskaia
F
r
e
q
1
1
5

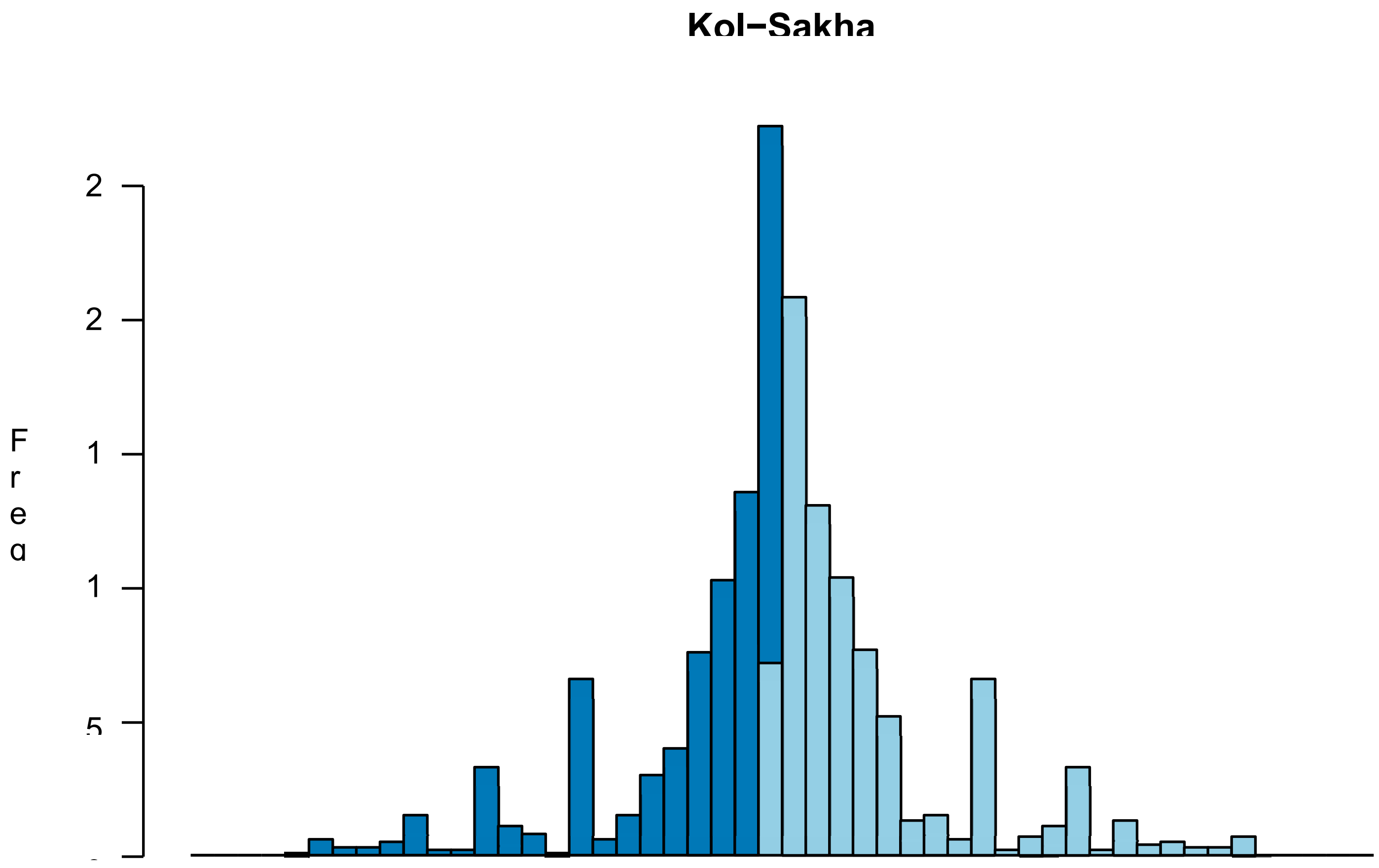
Kol−Sakha
F
r
e
q
2
2
1
1
5

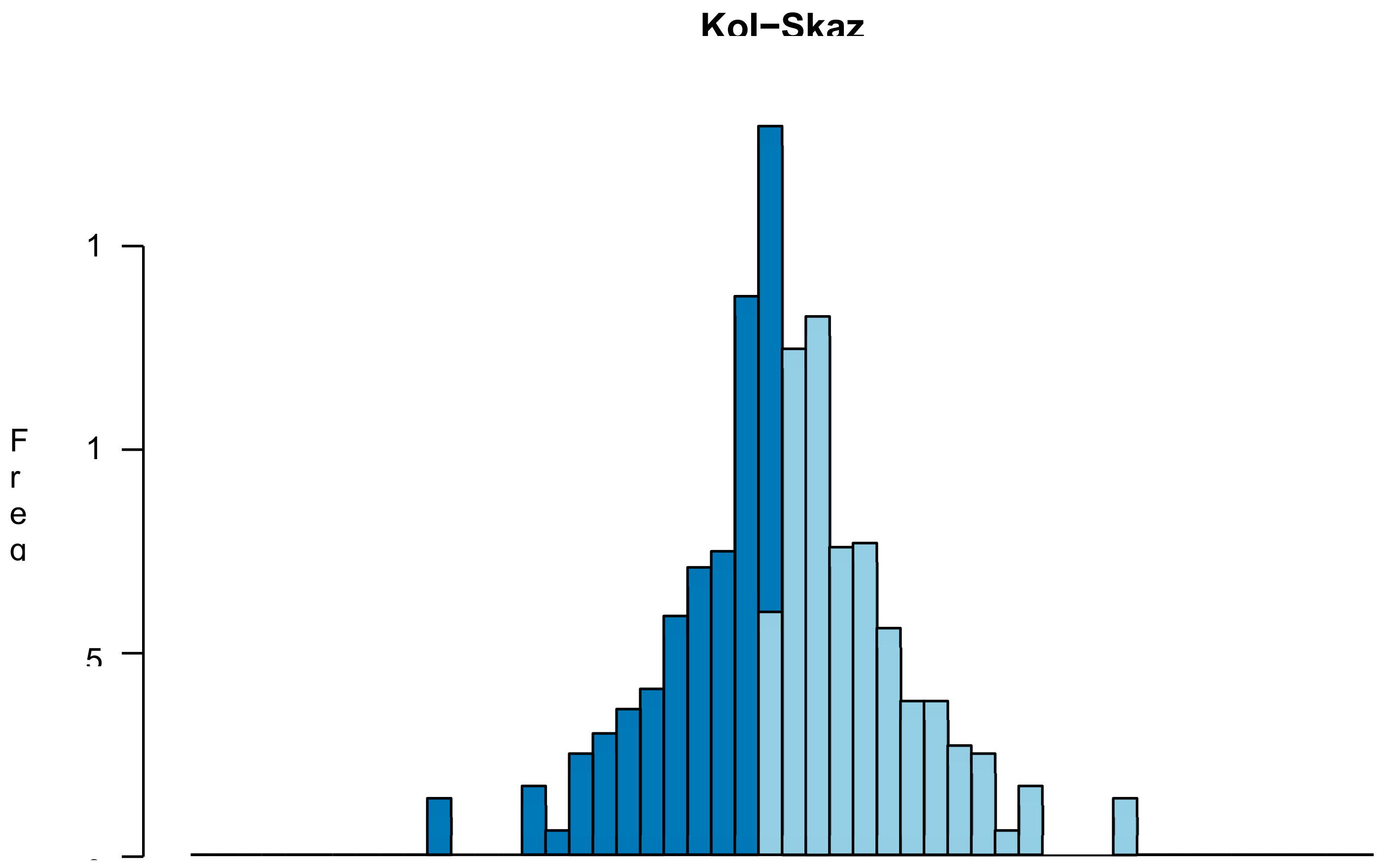

Kol−Skaz
F
r
e
q
1
1
5

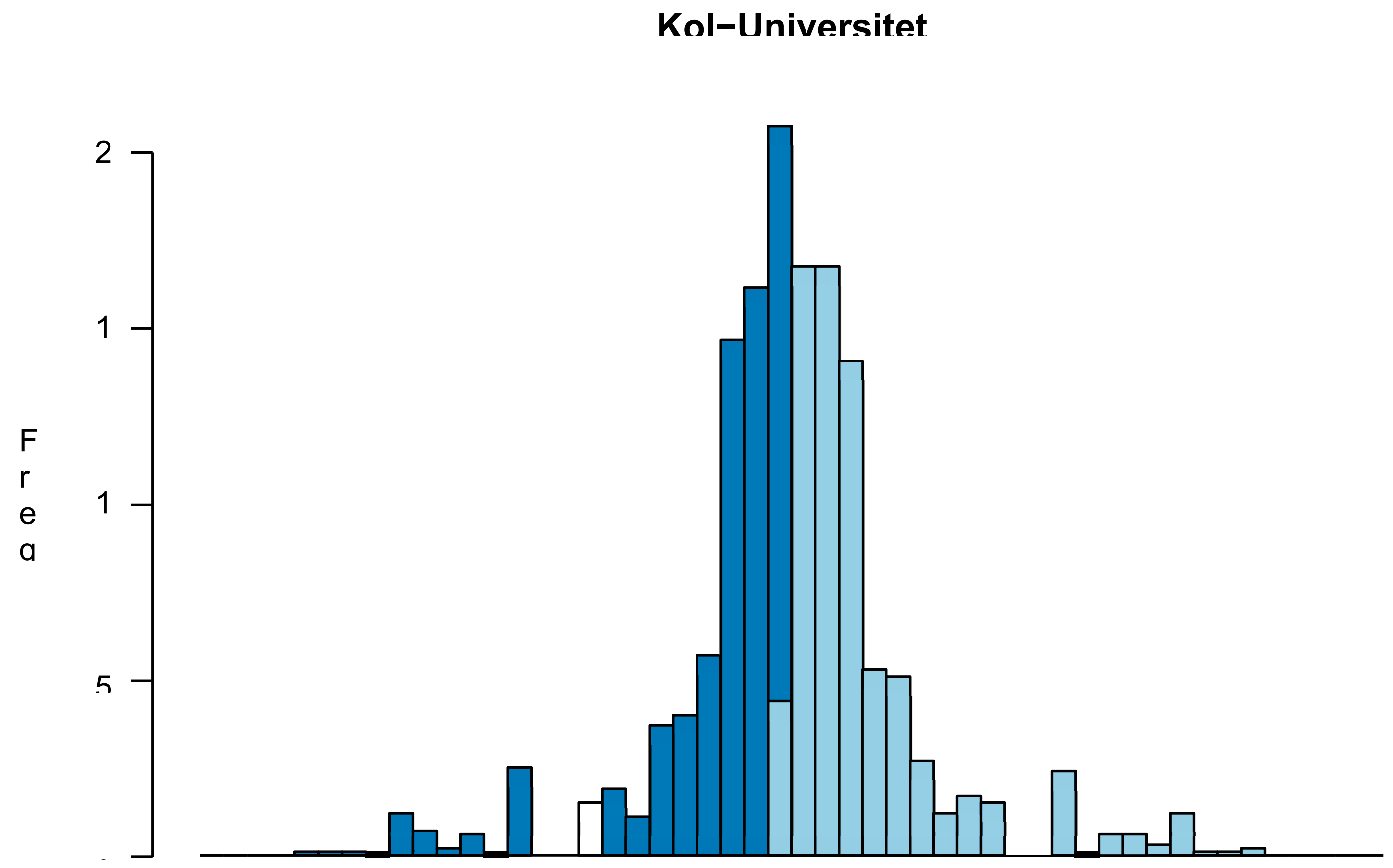
Kol−Universitet
F
r
e
q
2
1
1
5

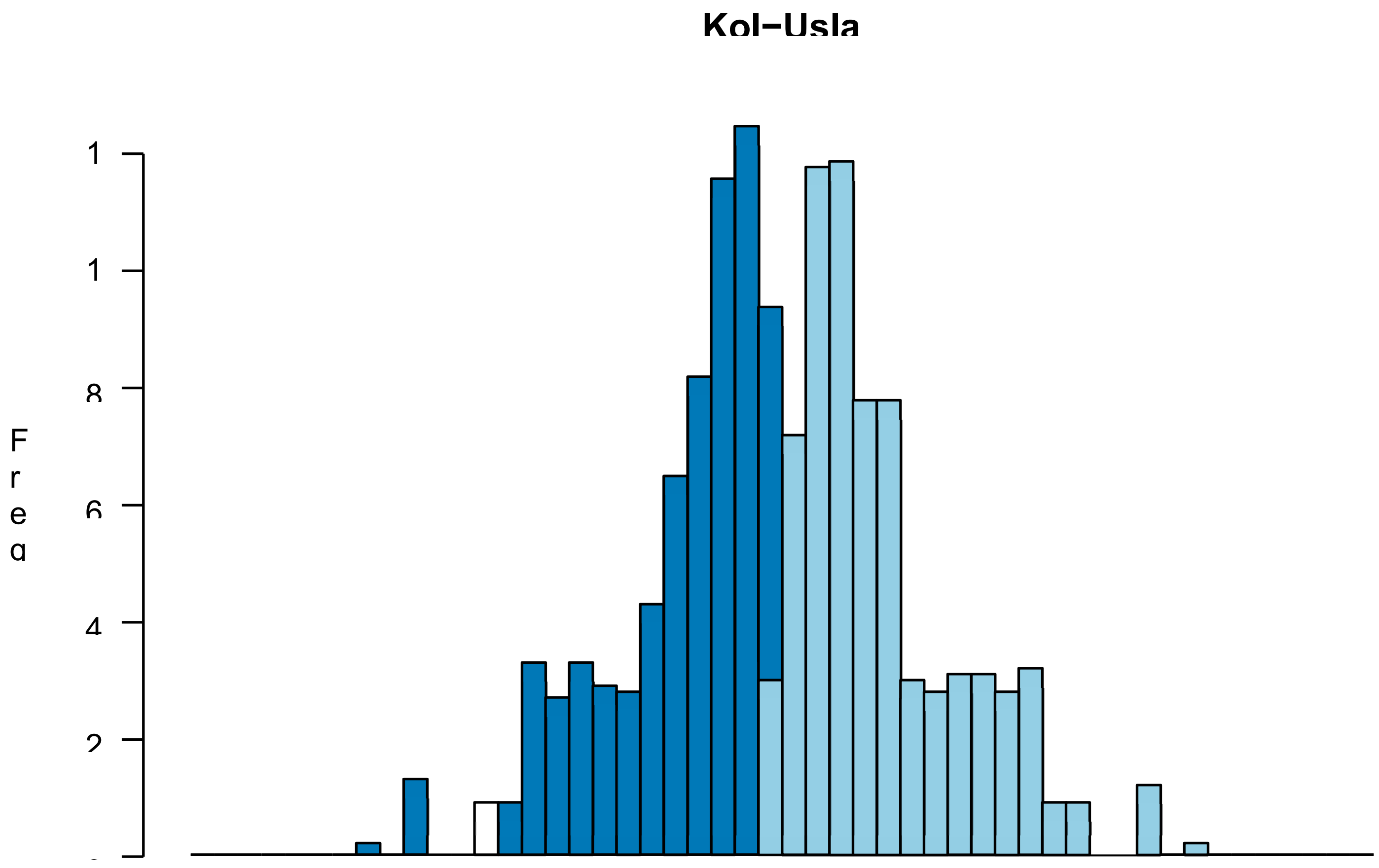
Kol-Usla
1
1
8
6
4
2
F
r
e
q

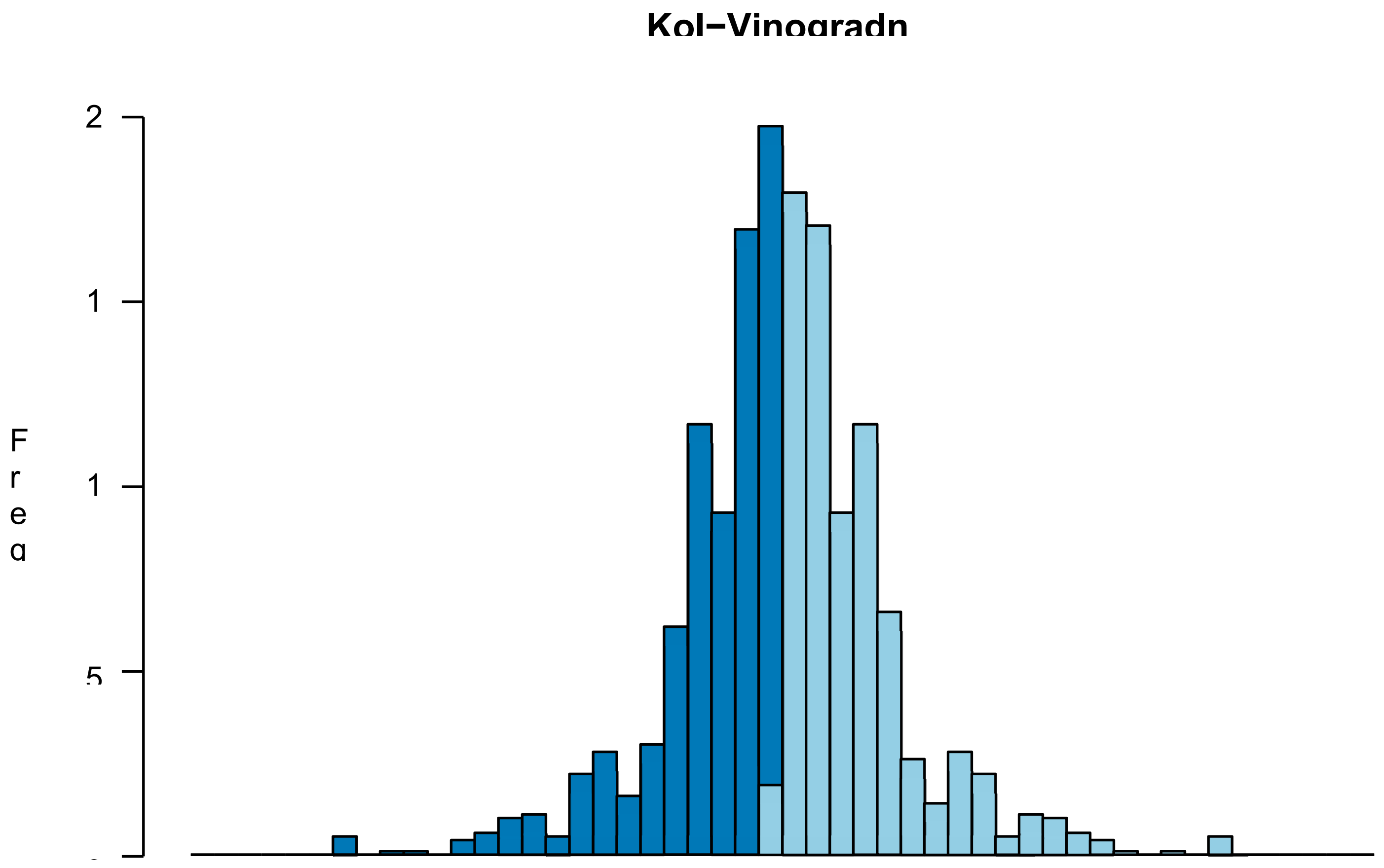

Kol−Vinogradn
2
1
F
r
e
q
1
5

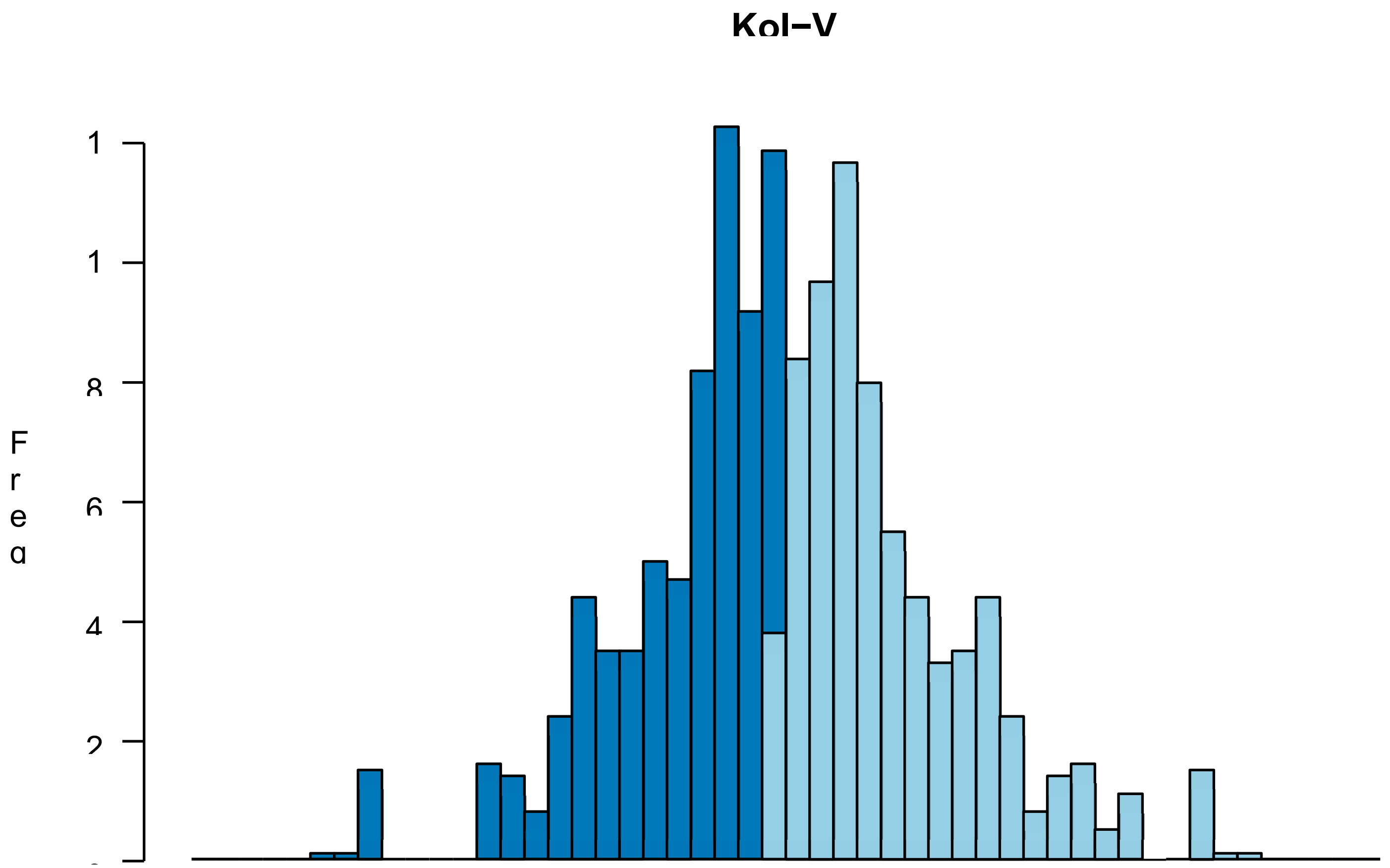

Kol−V
F
r
e
q
1
1
8
6
4
2

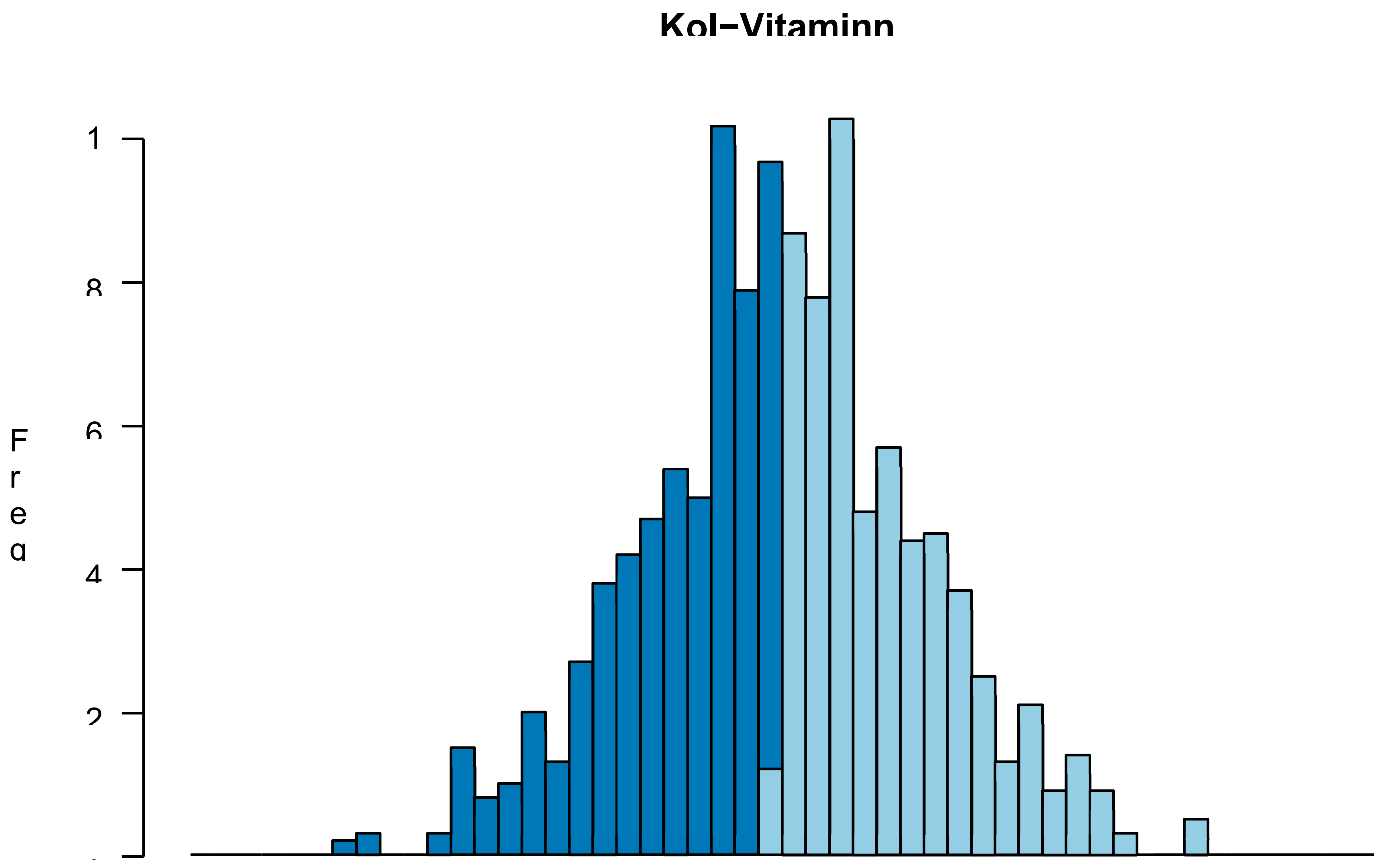

Kol−Vitaminn
F
r
e
q
1
8
6
4
2

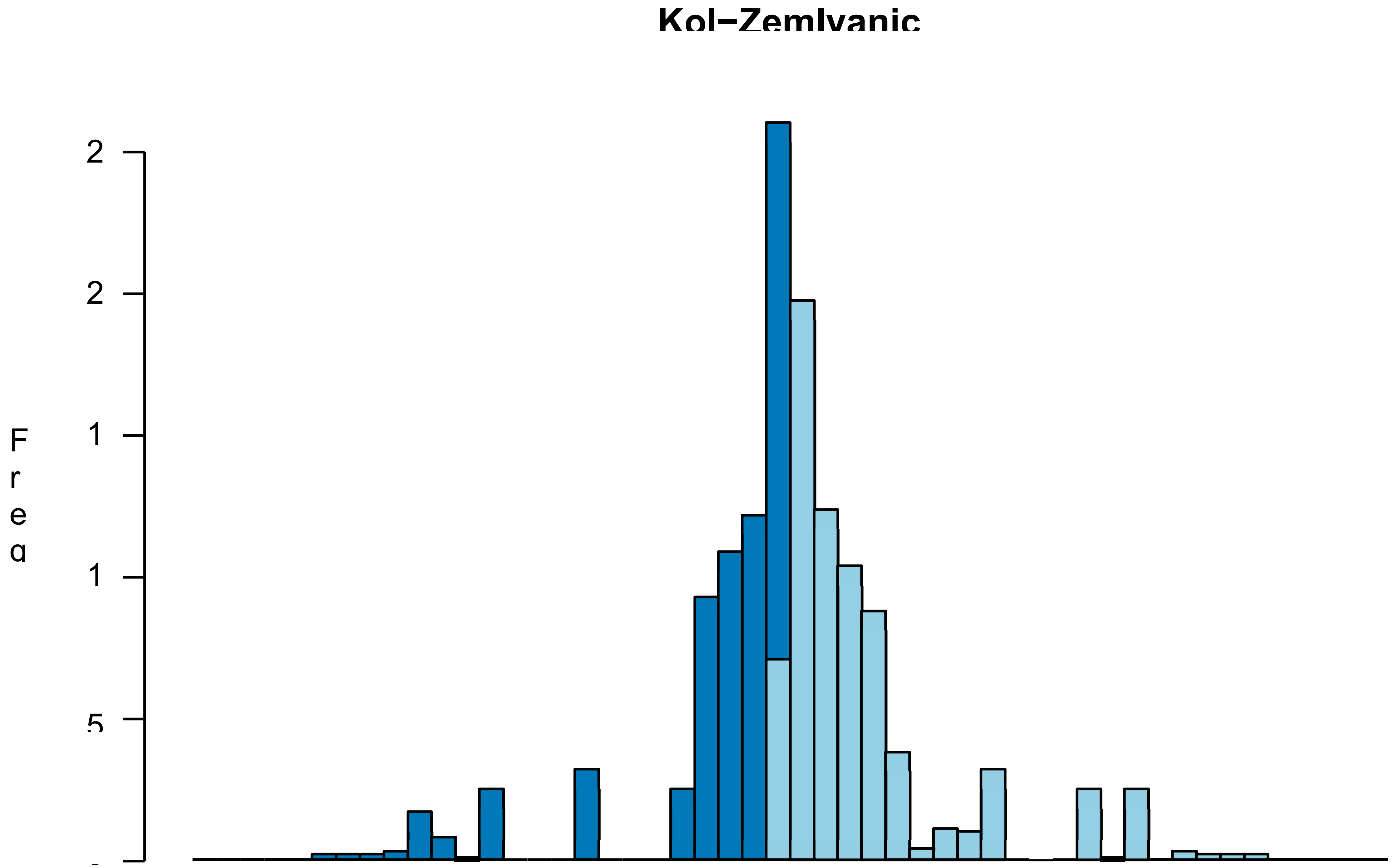
Kol−Zemlyanic
F
r
e
q
2
2
1
1
5

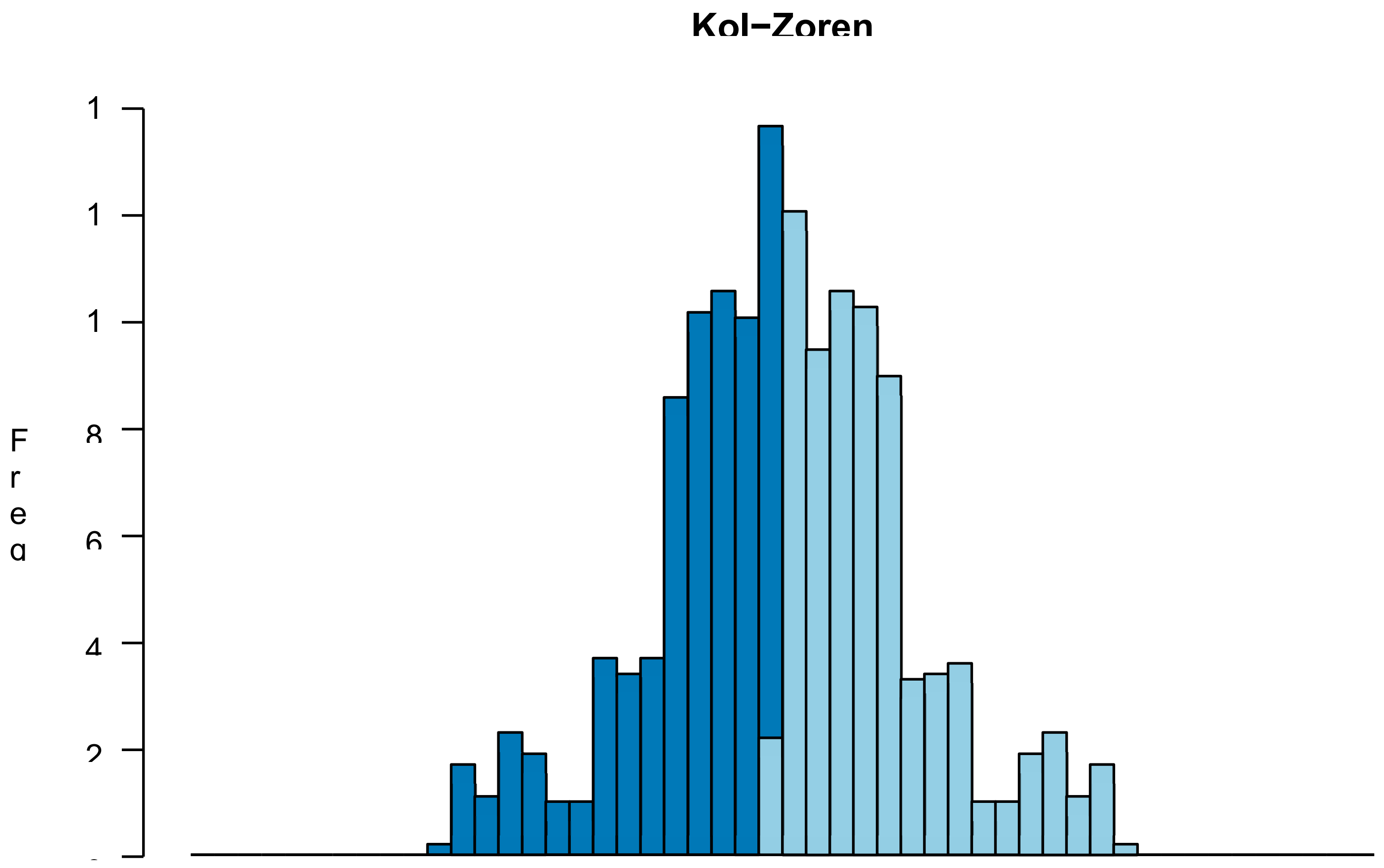

Kol–Zoren
1
1
1
8
6
4
2
F
r
e
q

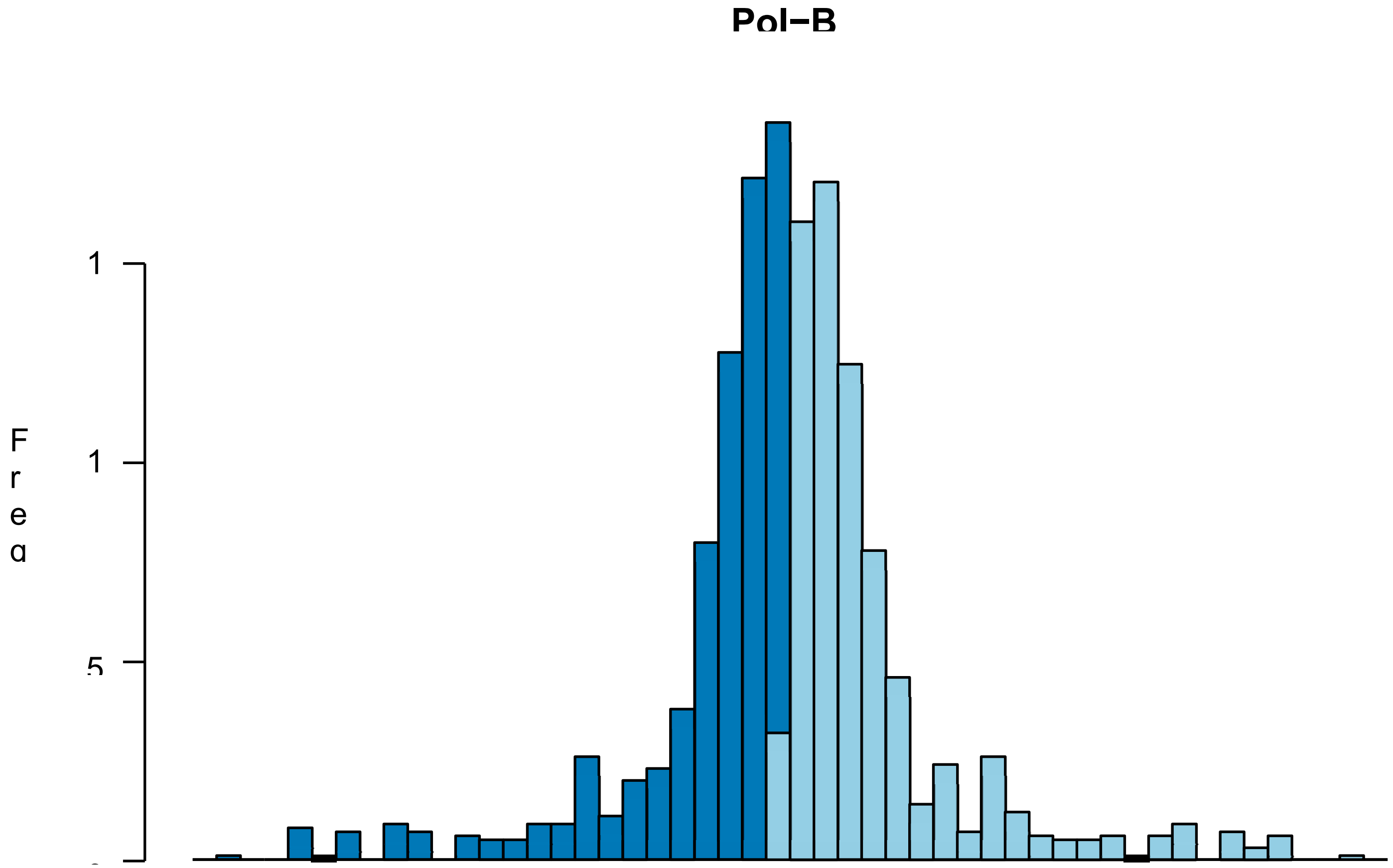

Pol−B
F
r
e
q
1
1
5

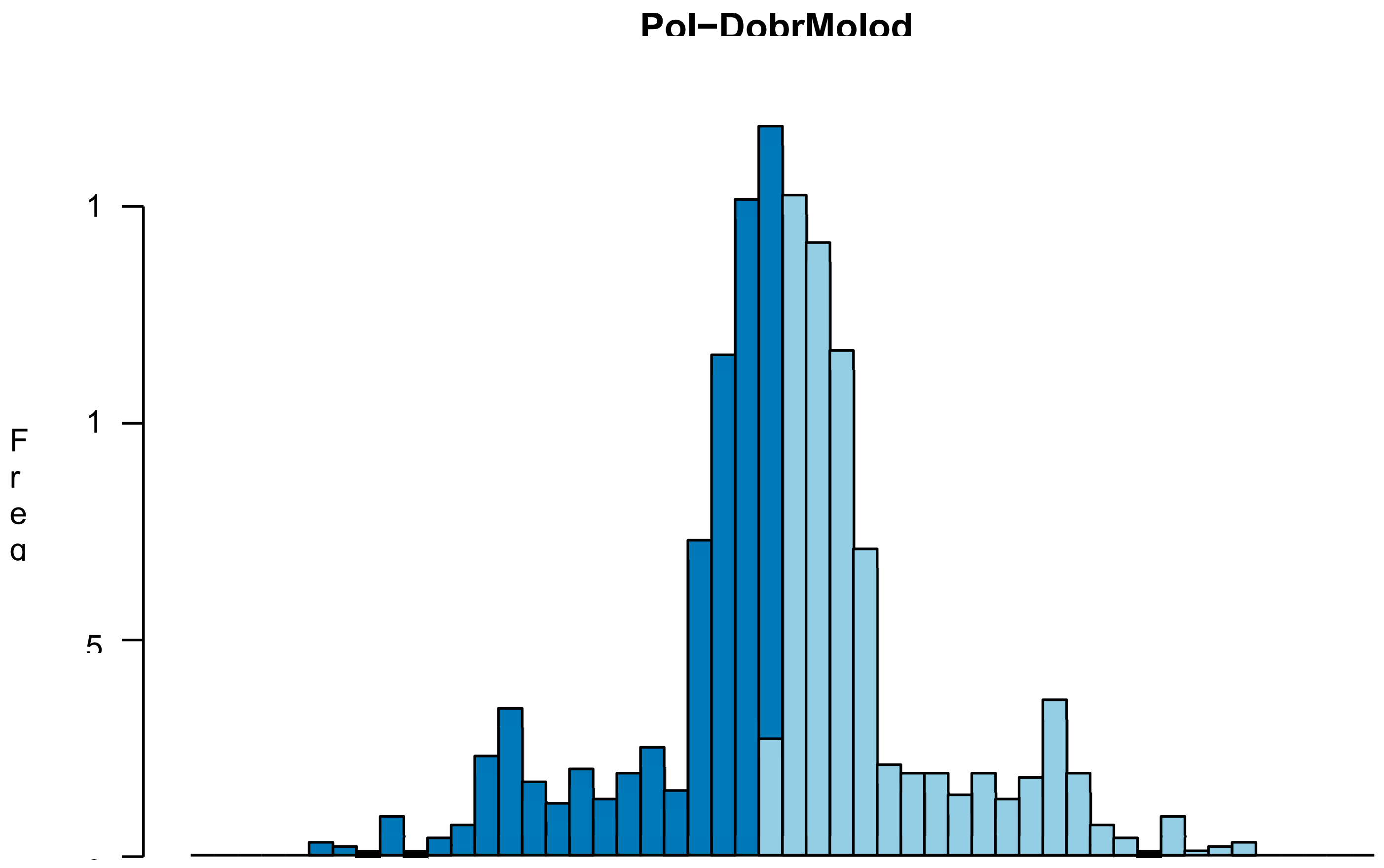

Pol−DobrMolod
F
r
e
q
1
1
5

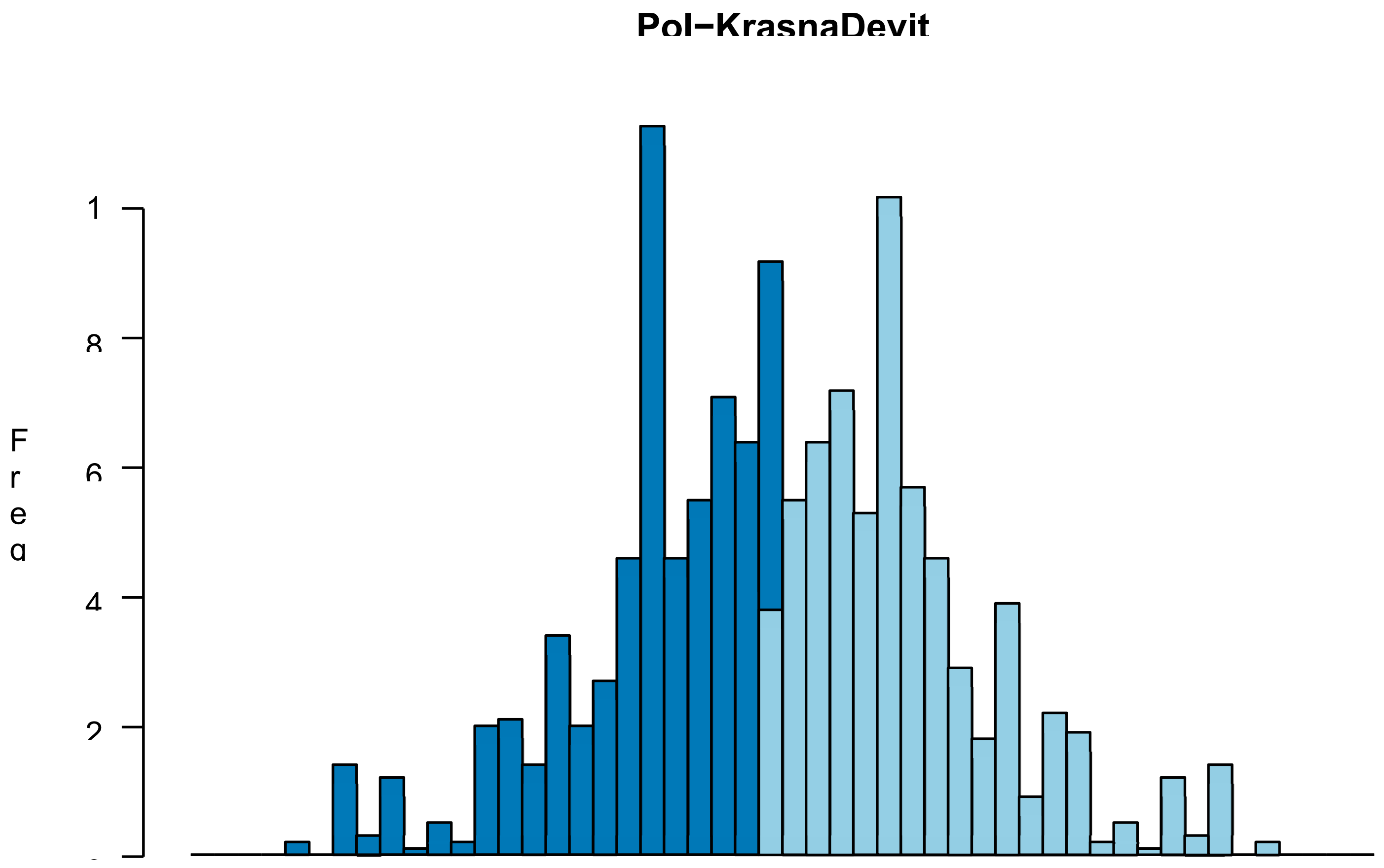

Pol−KrasnaDevit
Freq
1
8
6
4
2

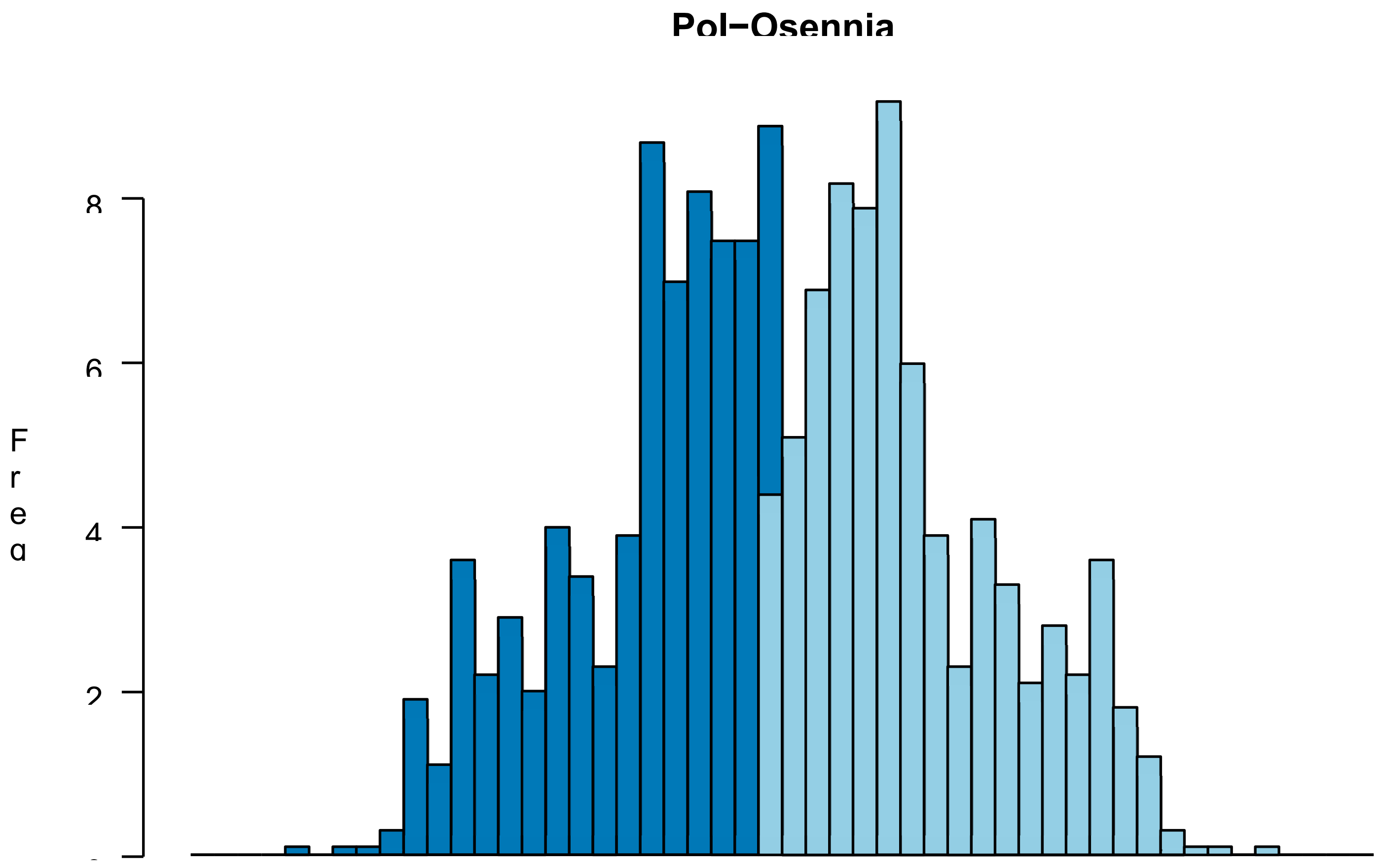

Pol−Osennia
8
6
4
2
F
r
e
q

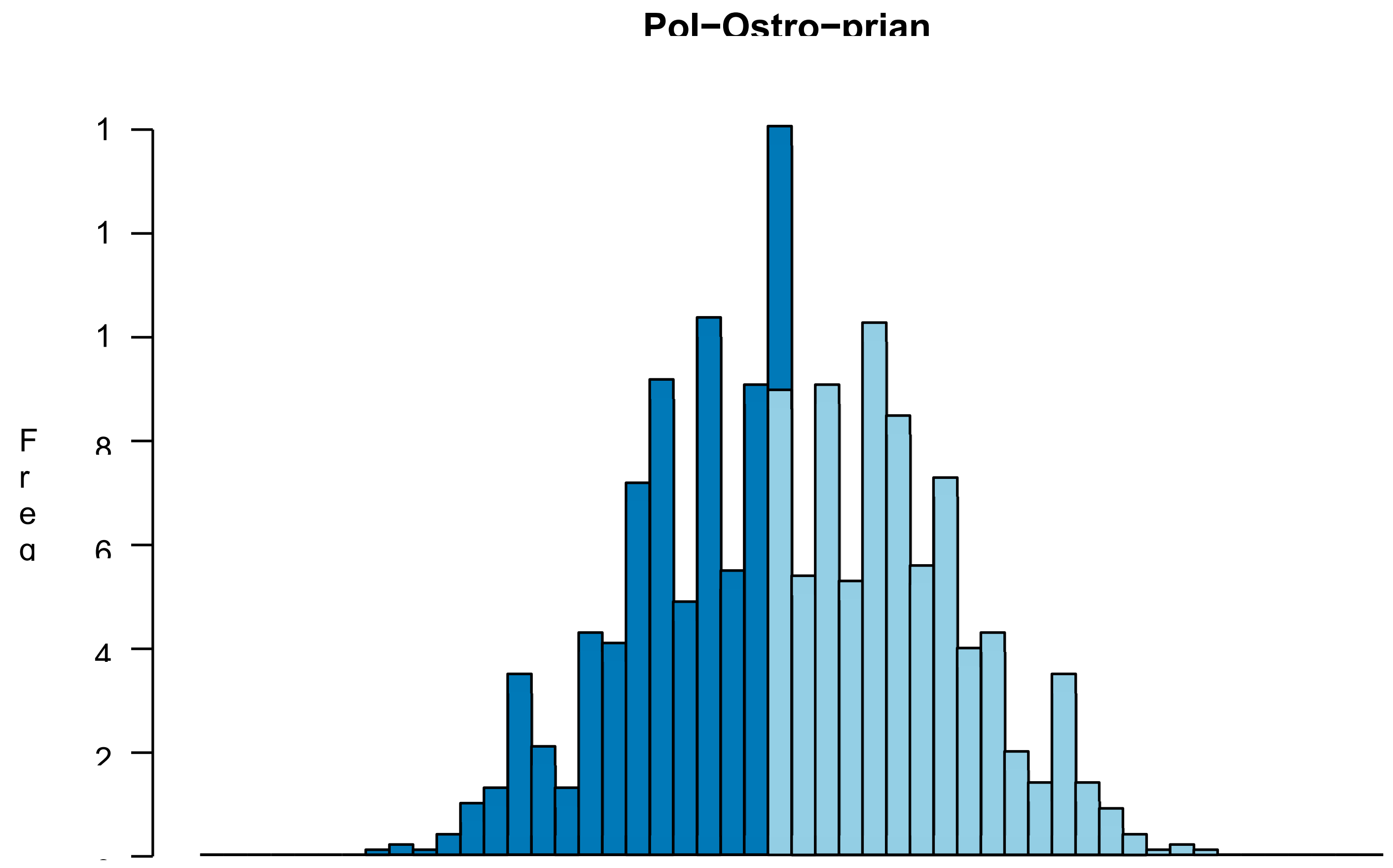
Pol−Ostro−prian

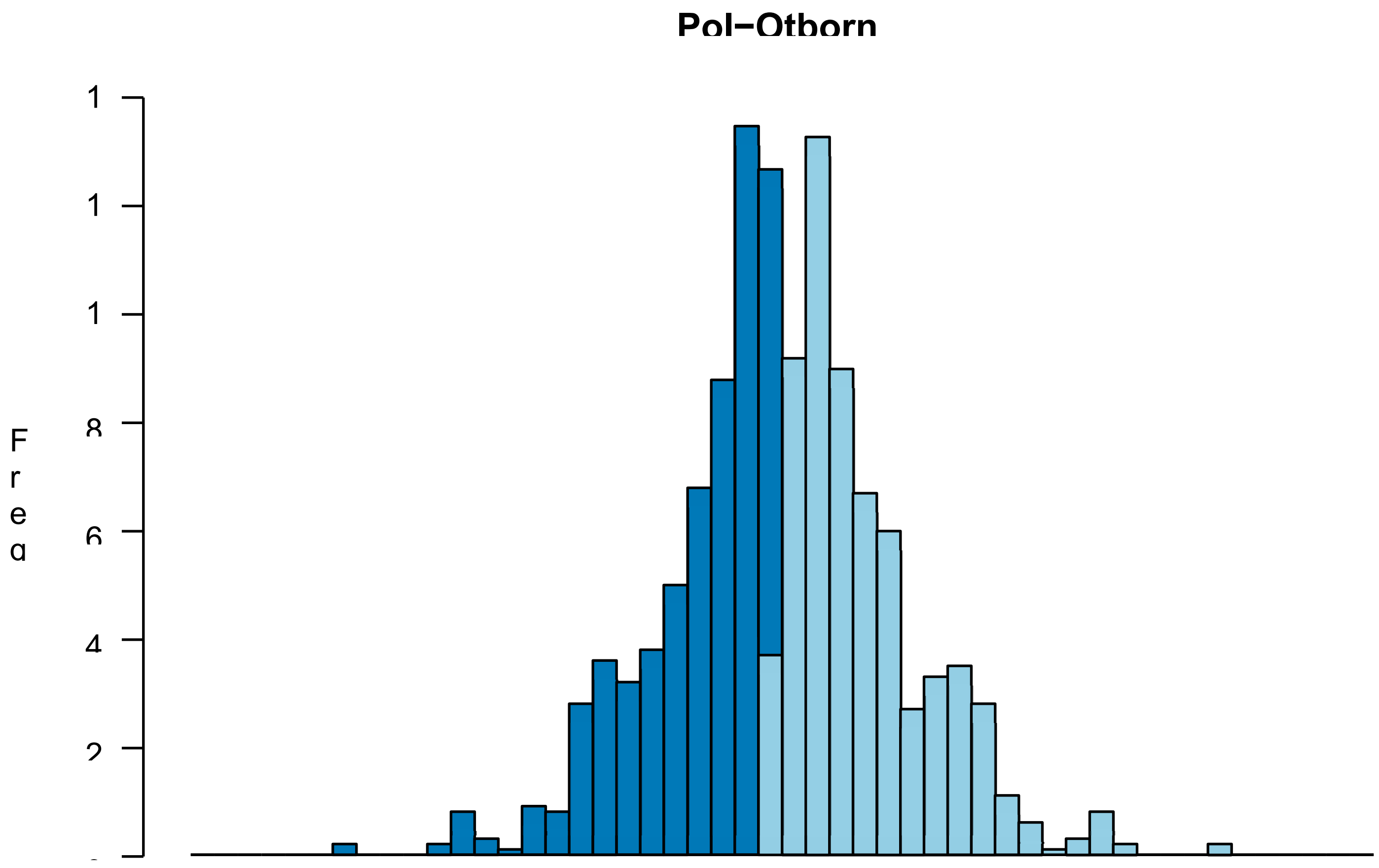

Pol−Otborn
F
r
e
q
1
1
1
8
6
4
2

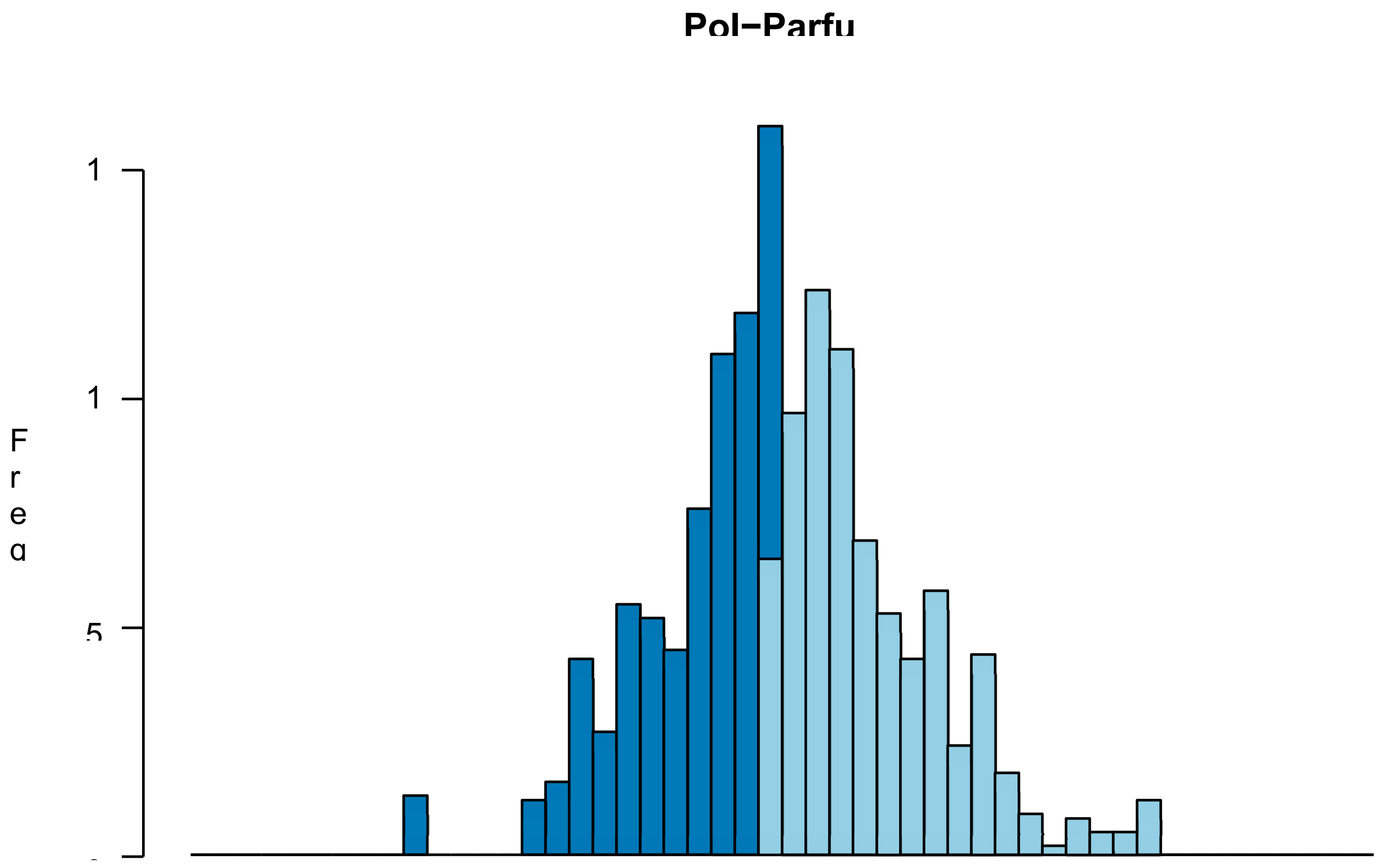

Pol−Parfu
1
1
5
F
r
e
q

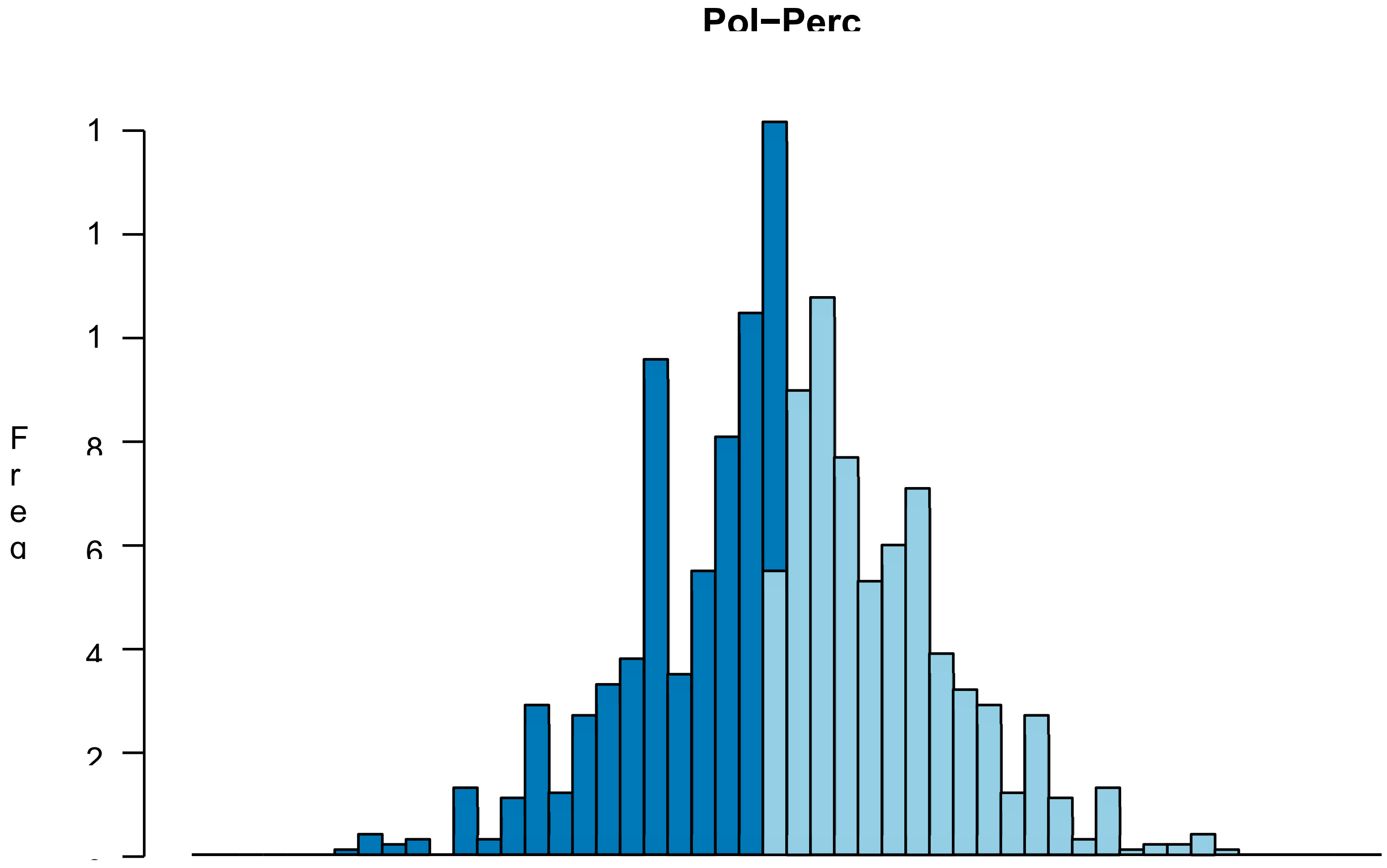
Pol−Perc
F
r
e
q
1
1
1
8
6
4
2

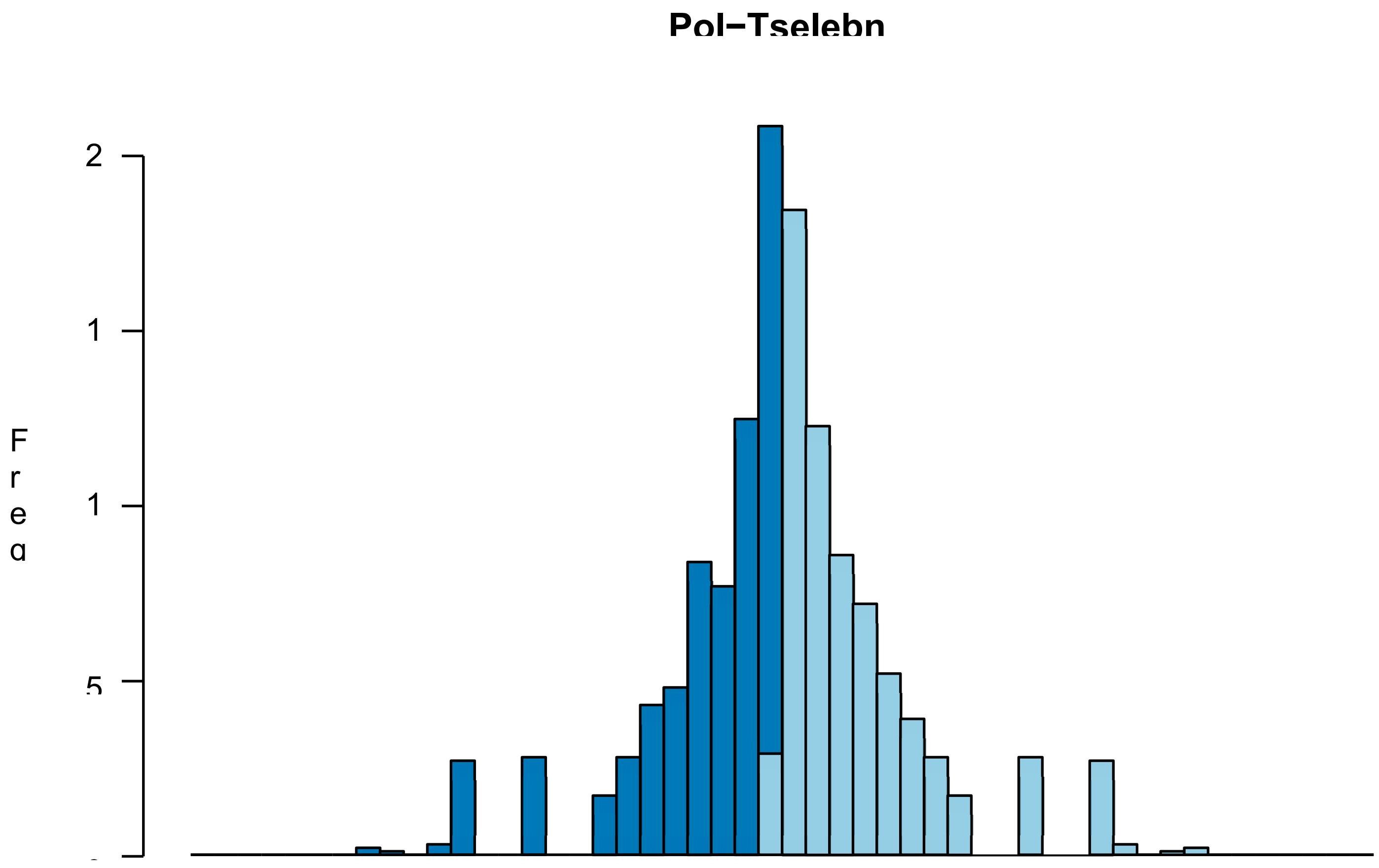

Pol−Tselebn
F
r
e
q
2
1
1
5

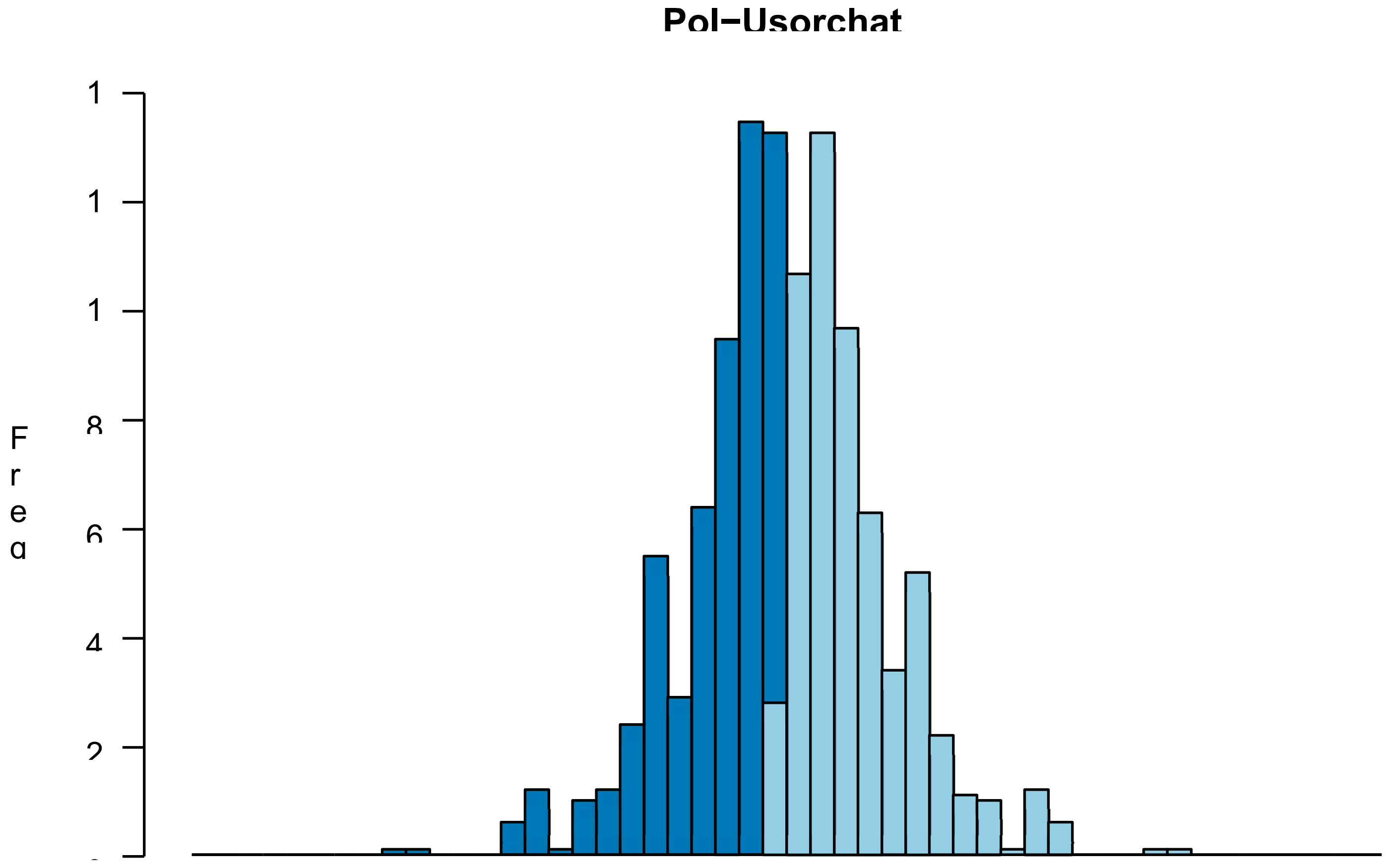
Pol−Usorchat
1
1
1
8
6
4
2
F
r
e
q

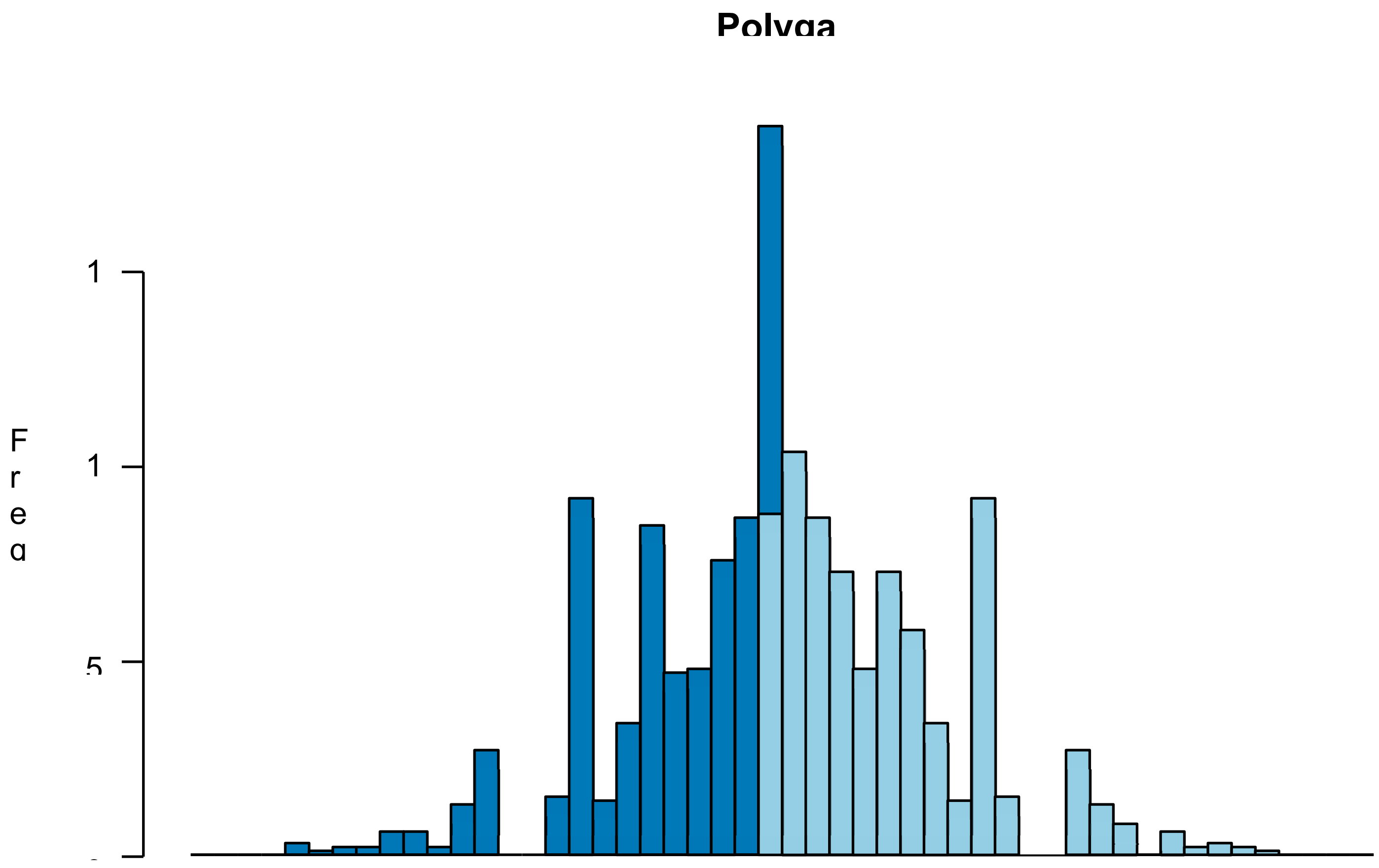
Polyga
F
r
e
q
1
1
5

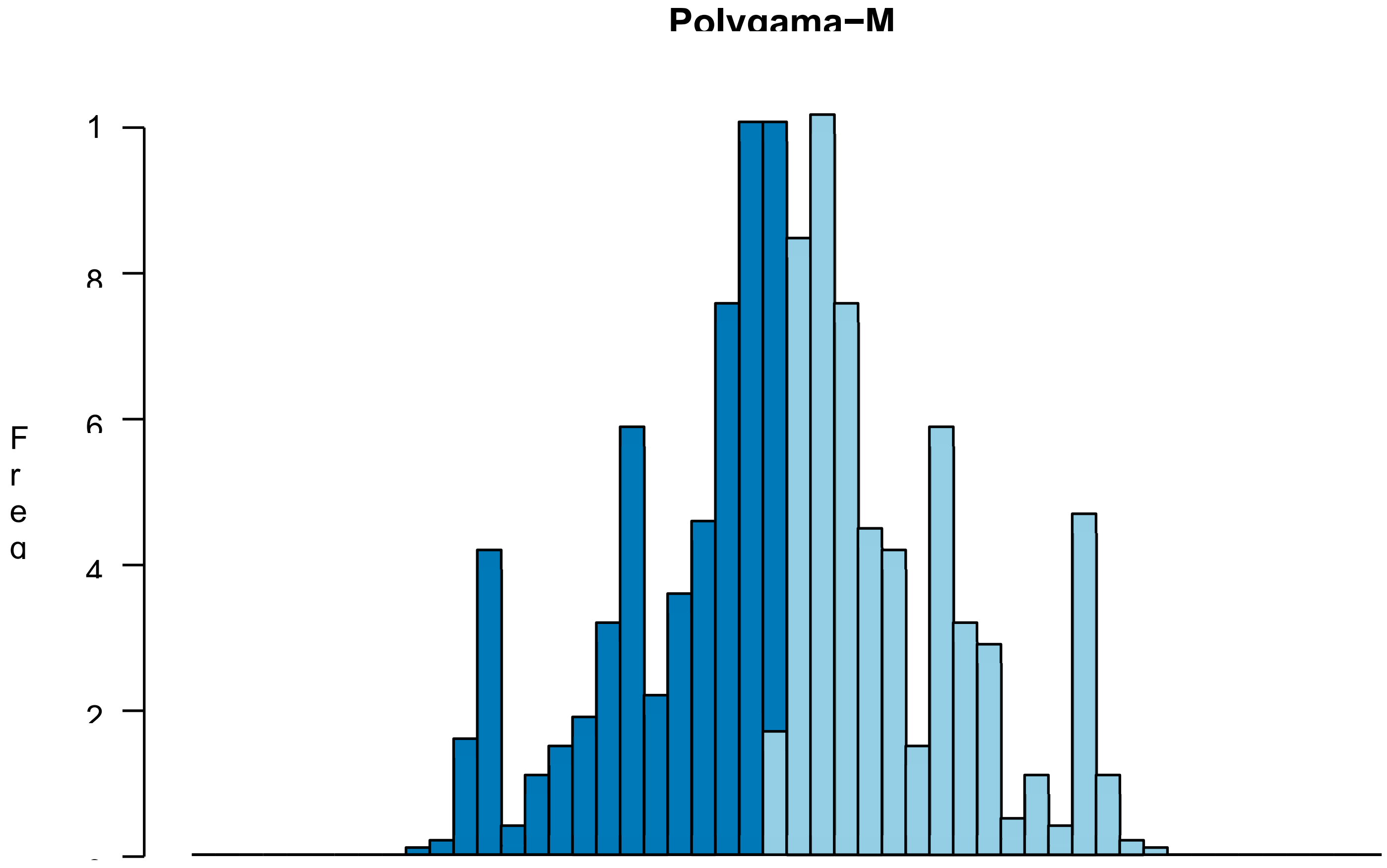
Polygama-M
1
8
6
4
2
F
r
e
q

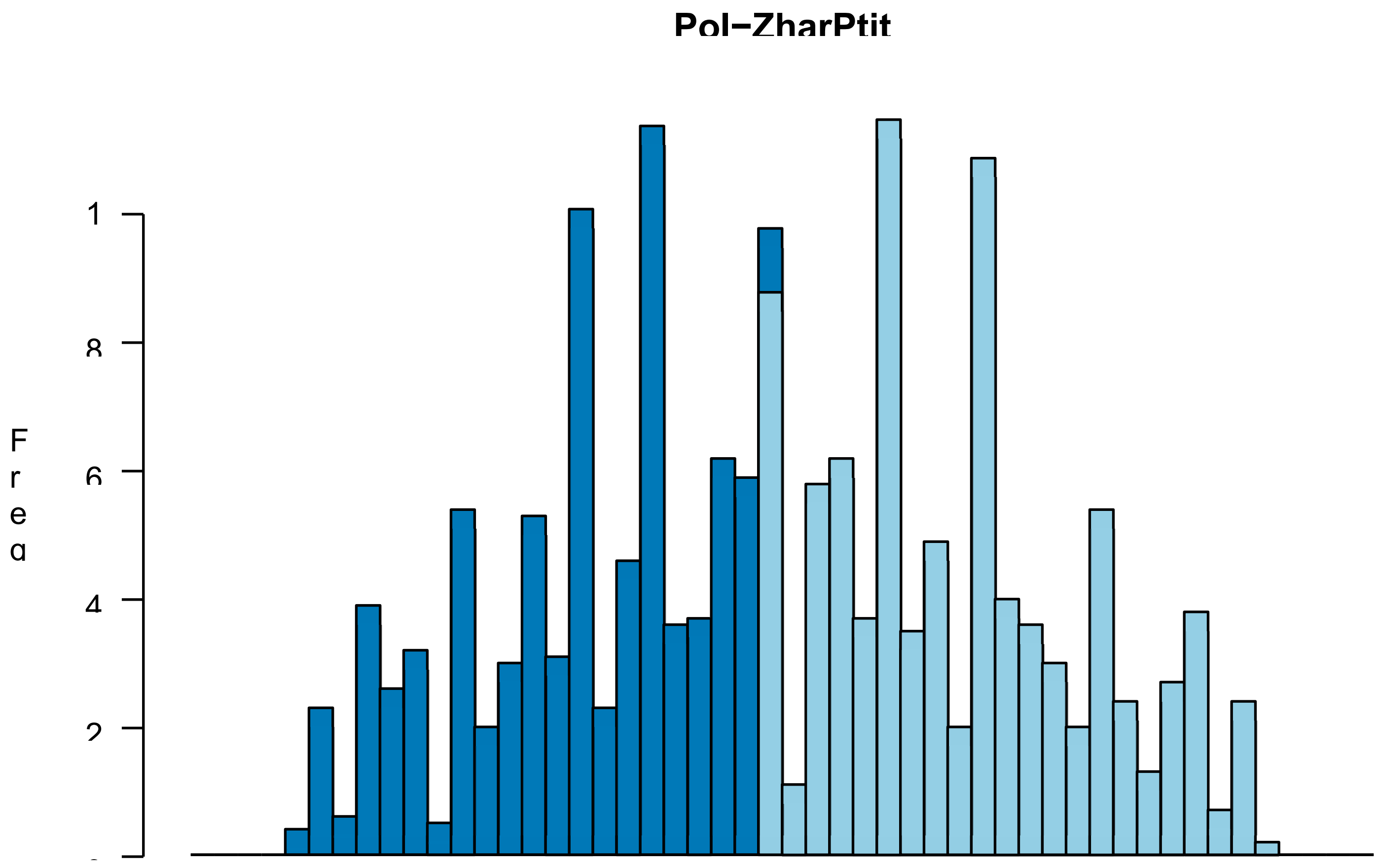

Pol−ZharPtit
F
r
e
q
1
8
6
4
2

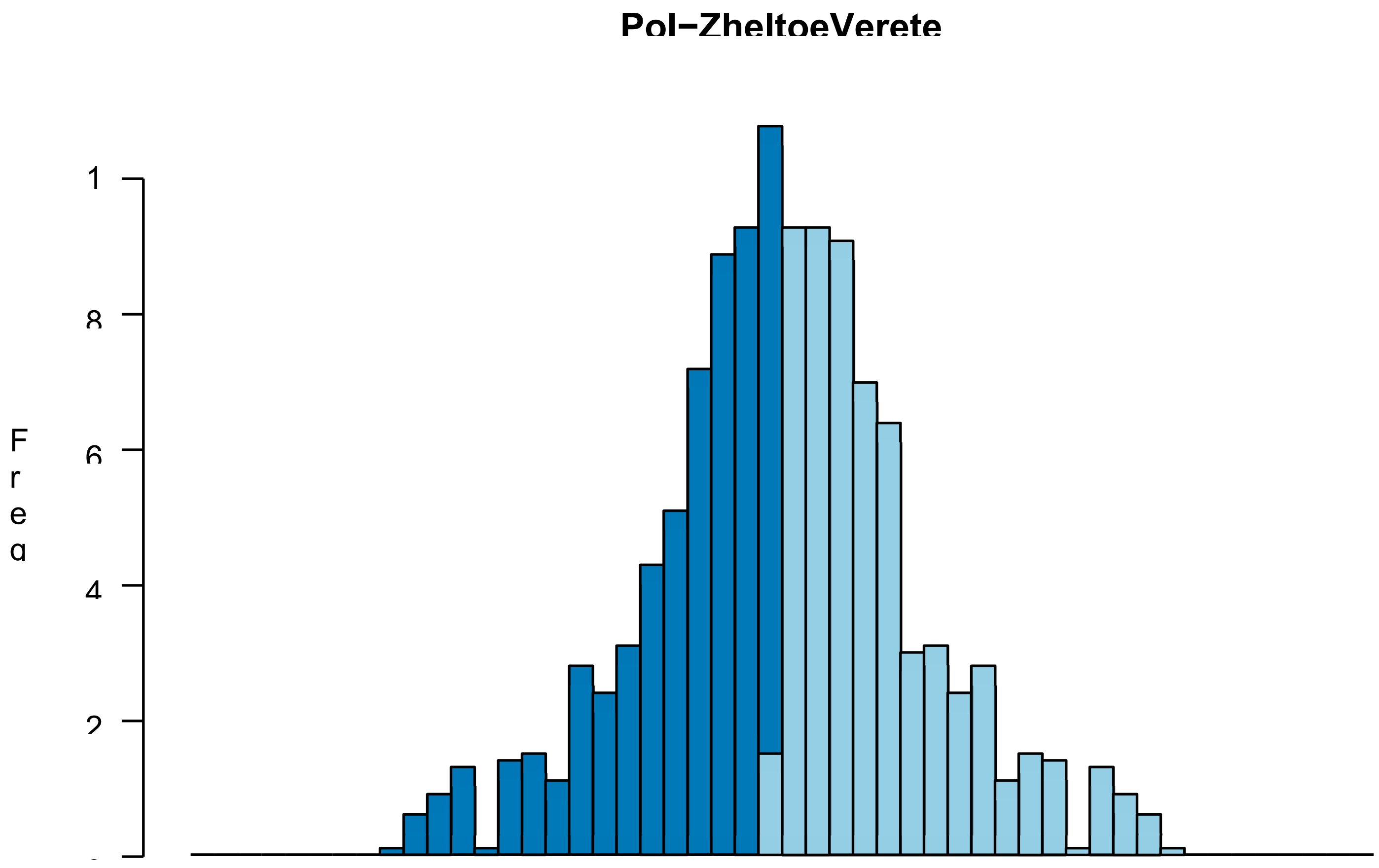
Pol−ZheltoeVerete
F
r
e
q
1
8
6
4
2

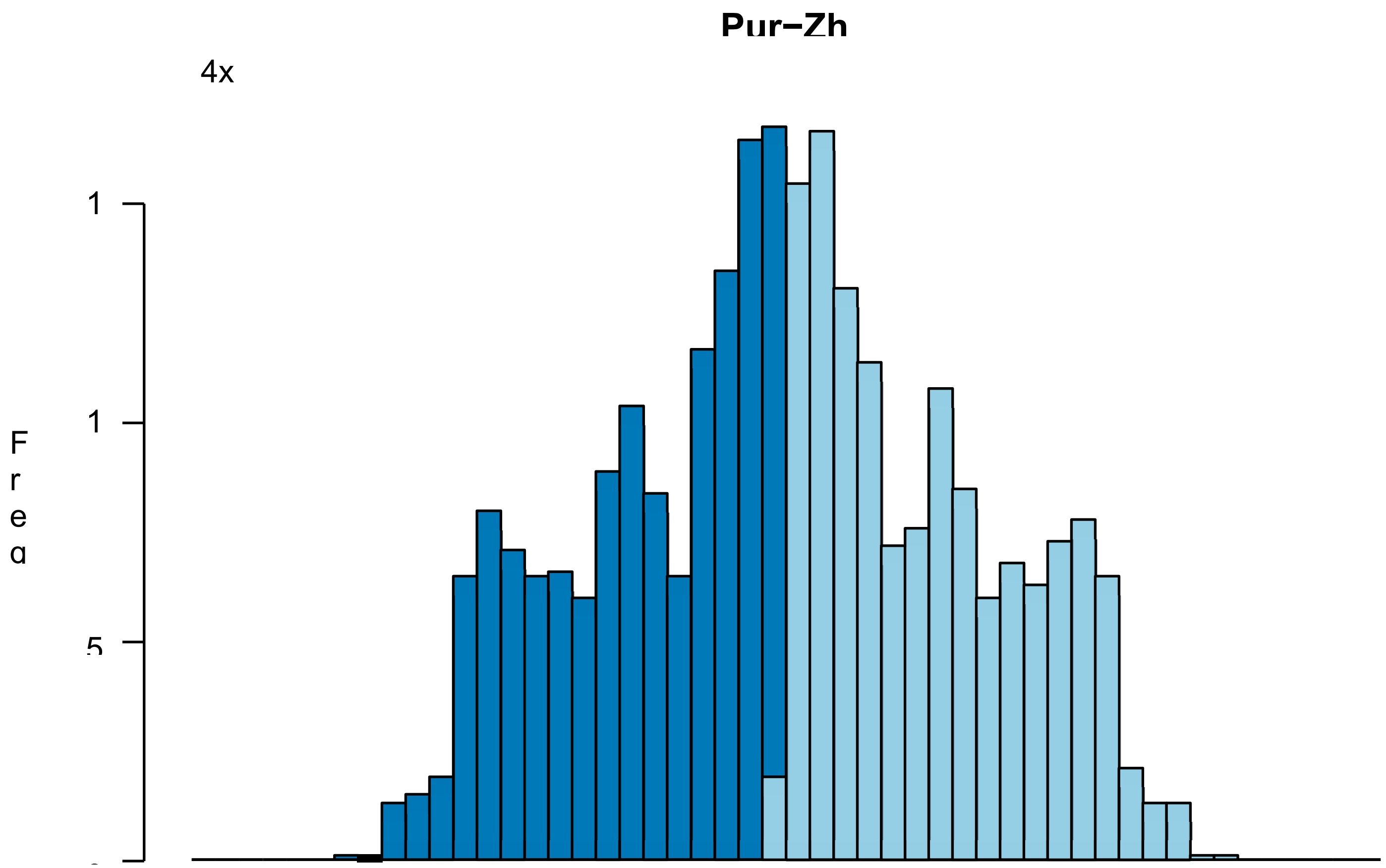
Pur–Zh
4x
1
1
5
F
r
e
q
