# Supplementary figures and images for: Genetic Diversity of the Collection of Far Eastern Actinidia spp. Revealed by RAD Sequencing Technology
Source: Plants (Basel). 2024 Dec 24;14(1):7. doi: 10.3390/plants14010007 (PMC11723124; doi:10.3390/plants14010007)

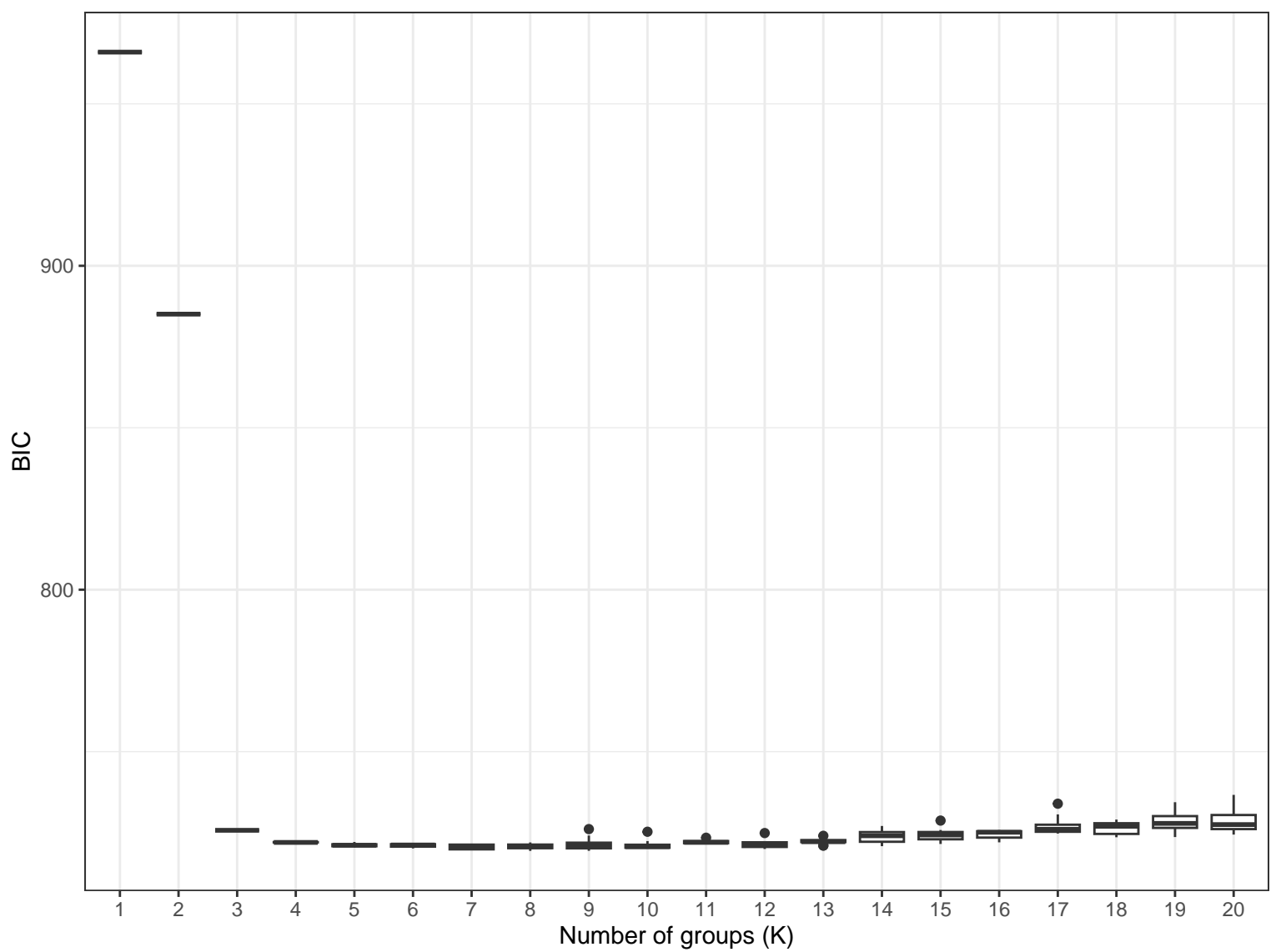

Supplement: Supplementary file 1 [file plants-14-00007-s001.zip › plants-3356746-supplementary/Supplementary/Figure S2.pdf]

# Arguta

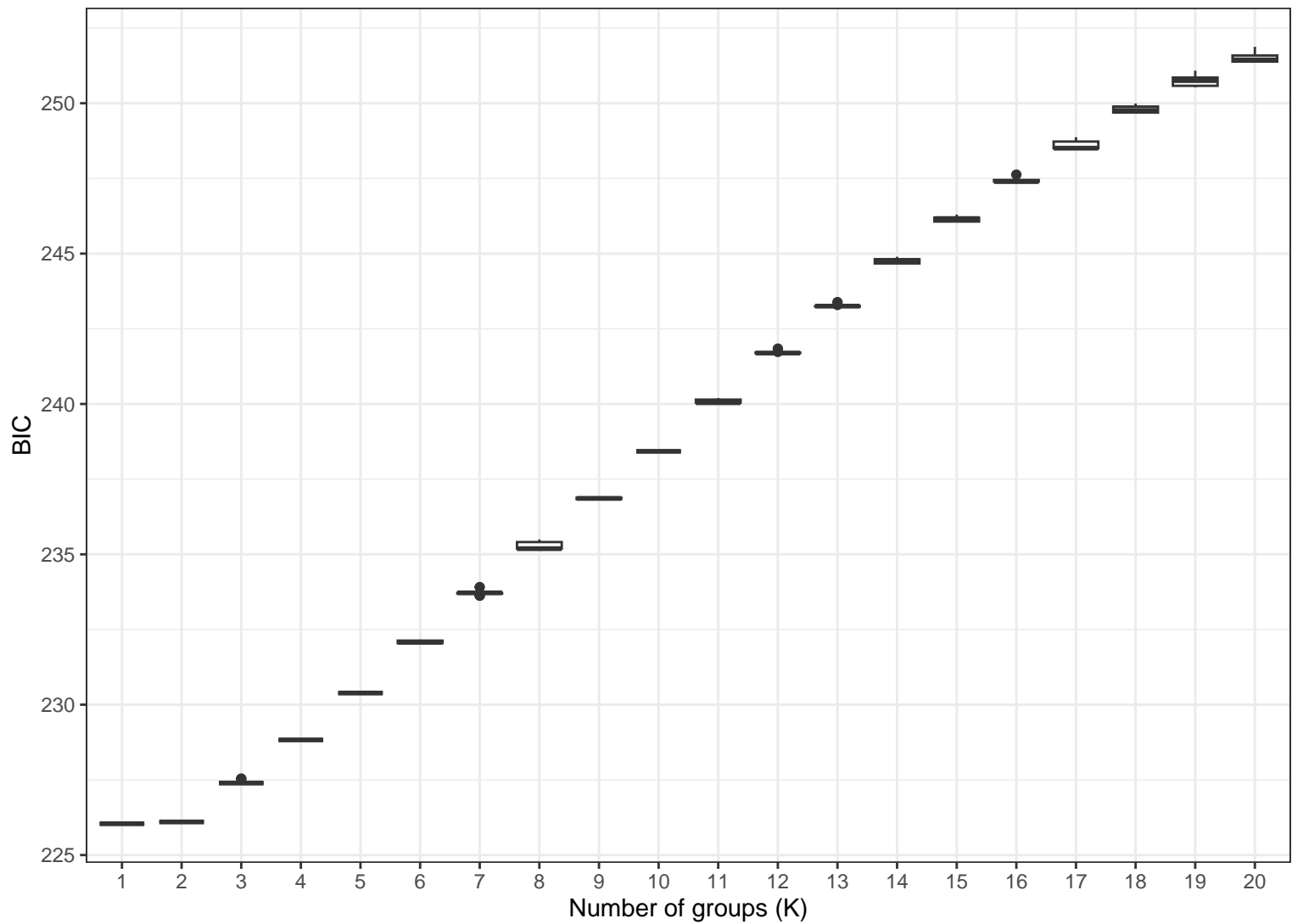

Supplement: Supplementary file 1 [file plants-14-00007-s001.zip › plants-3356746-supplementary/Supplementary/Figure S3.pdf]

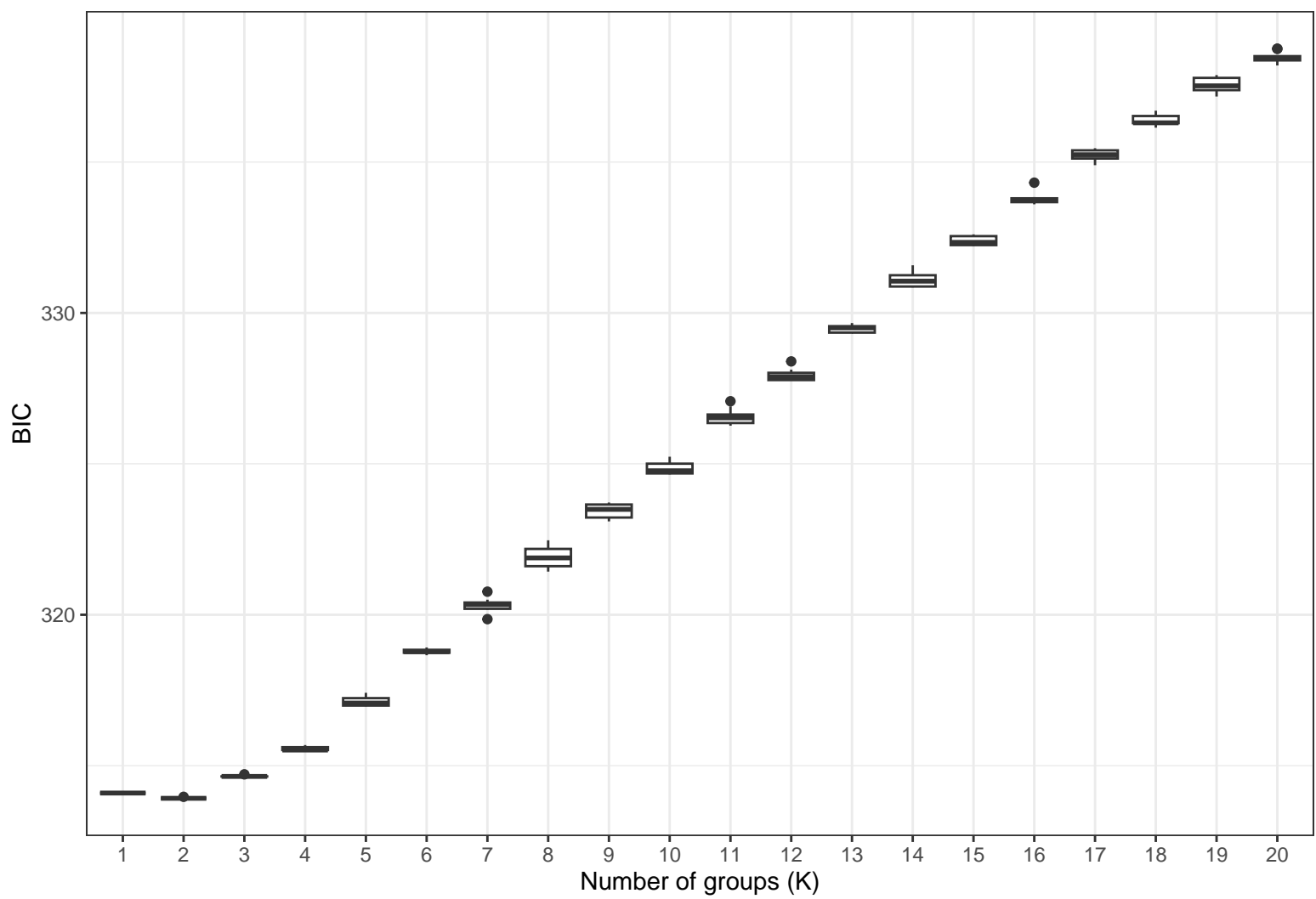

Supplement: Supplementary file 1 [file plants-14-00007-s001.zip › plants-3356746-supplementary/Supplementary/Figure S4.pdf]

# Polygama

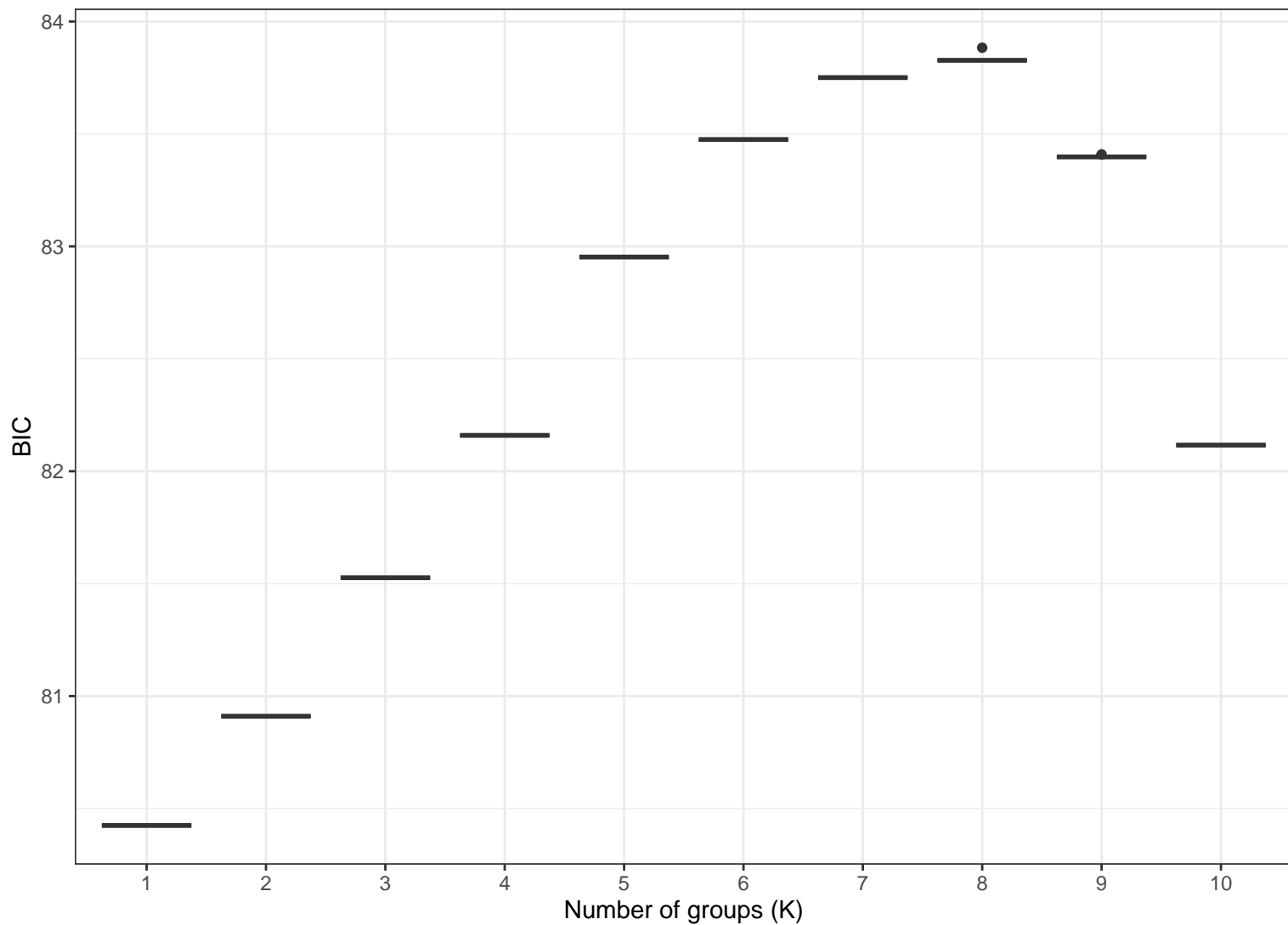

Supplement: Supplementary file 1 [file plants-14-00007-s001.zip › plants-3356746-supplementary/Supplementary/Figure S5.pdf]
